# Body Weight-Related Parameters in Pregnancies Complicated by Type 2 Diabetes Mellitus: A Systematic Review and Meta-Analysis with Maternal and Perinatal Outcome Mapping

**DOI:** 10.3390/jcm15135260

**Published:** 2026-07-06

**Authors:** Katarina Ivanovic, Andja Cirkovic, Stefan Dugalic, Milos Milincic, Maja Macura, Miroslava Gojnic Dugalic

**Affiliations:** 1 Clinic for Gynecology and Obstetrics, University Clinical Centre of Serbia, 11000 Belgrade, Serbia; stef.dugalic@gmail.com (S.D.); milosmilincic@gmail.com (M.M.); maja_macura@live.com (M.M.); miroslavagojnicdugalic@yahoo.com (M.G.D.); 2 Institute for Medical Statistics and Informatics, Faculty of Medicine, University of Belgrade, 11000 Belgrade, Serbia; 3 Faculty of Medicine, University of Belgrade, 11000 Belgrade, Serbia

**Keywords:** type 2 diabetes mellitus, pre-pregnancy body mass index, gestational weight gain, maternal obesity, maternal outcomes, neonatal outcomes, systematic review, meta-analysis

## Abstract

**Background:** Type 2 diabetes mellitus (T2DM) in pregnancy is increasingly recognized as a high-risk metabolic condition, frequently accompanied by overweight, obesity, insulin resistance, and adverse maternal and neonatal outcomes. This systematic review and meta-analysis aimed to evaluate body weight-related parameters in pregnancies complicated by T2DM compared with those in non-T2DM control groups. **Methods:** A systematic search of PubMed, Scopus, and Web of Science was conducted up to 19 August 2025. Original studies reporting body weight, body mass index (BMI), or gestational weight gain (GWG) in pregnant women with T2DM and different control groups were included. Data were synthesized using standardized mean differences (SMDs) with fixed or random-effects models. Maternal, metabolic, delivery, and neonatal outcomes were summarized descriptively. **Results:** Eighty-seven studies were included in the systematic review and seventy-two were included in the meta-analysis. Pregnant women with T2DM had significantly higher pre-pregnancy body weight and first-trimester body weight than normoglycemic and T1DM controls. Pre-pregnancy BMI was also significantly higher in T2DM pregnancies compared with normoglycemic, T1DM, and gestational diabetes controls. In contrast, GWG did not differ significantly between T2DM and normoglycemic or gestational diabetes pregnancies, while it was significantly lower in T2DM than in type 1 diabetes pregnancies. Adverse maternal and neonatal outcomes, including hypertensive disorders, preterm delivery, fetal growth abnormalities, macrosomia, congenital anomalies, and fetal/neonatal loss, were frequently reported across the included studies. **Conclusions:** Pregnancies complicated by T2DM are characterized by an unfavorable preconception anthropometric profile. The contrasting patterns across diabetes types suggest different periods for weight-related care: preconception weight optimization appears particularly relevant in T2DM, whereas the higher gestational weight gain observed in T1DM relative to T2DM supports individualized monitoring of gestational weight trajectories during pregnancy. These strategies should be incorporated into comprehensive preconception and antenatal care alongside glycemic optimization and assessment of diabetes-related complications.

## 1. Introduction

Type 2 diabetes mellitus (T2DM) in pregnancy represents an increasingly important clinical and public health challenge [1,2]. Although gestational diabetes mellitus (GDM) remains the most common form of impaired glucose metabolism in pregnancy, recent decades have been marked by a rising prevalence of pre-existing type 2 diabetes among women of reproductive age. This trend is largely associated with the global increase in the prevalence of overweight and obesity, sedentary lifestyle, changes in reproductive behavior, and delayed childbearing [2,3]. Unlike GDM, which usually develops during the second or third trimester of pregnancy and often resolves after delivery, T2DM is characterized by chronic insulin resistance and β-cell dysfunction that precede conception [4,5]. As a result, pregnant women with T2DM enter pregnancy with an already unfavorable metabolic environment, often accompanied by obesity, dyslipidemia, elevated arterial blood pressure, and other components of the metabolic syndrome [5,6]. Therefore, these pregnancies cannot be viewed solely and exclusively through the disturbance of glycemic status, but rather as a complex metabolic condition in which hyperglycemia, altered lipid metabolism, and chronic low-grade inflammation are intertwined. If overweight or obesity is also present, the degree of metabolic burden is further increased [6,7].

Body weight-related parameters are clinically relevant because previous studies have associated them with an unfavorable course of pregnancy, whereas the primary focus of the present study was placed on their distribution and differences between pregnant women with type 2 diabetes and appropriate control groups [8,9,10]. Among these factors, pre-pregnancy body weight, pre-pregnancy body mass index, and gestational weight gain occupy a particularly important place. These parameters are easily measurable, available in routine clinical practice, and potentially amenable to intervention through preconception counseling, nutritional management, and individualized monitoring during pregnancy [9,10,11]. Pre-pregnancy body mass index reflects the metabolic status of a woman before conception, whereas gestational weight gain represents a dynamic indicator of changes during pregnancy. General recommendations for gestational weight gain based on pre-pregnancy body mass index categories are primarily intended for the general population of pregnant women [11]. For women with pre-existing diabetes, particularly type 2 diabetes, sufficiently precise and universally accepted recommendations do not exist. This is important because, in this population, metabolic risk is not determined solely by body mass index category, but also by the presence of chronic insulin resistance, impaired glycemic regulation, and frequently associated cardio metabolic risk factors [5,6,7,12]. Therefore, optimal gestational weight gain in this population may not necessarily correspond to recommendations for metabolically healthy pregnant women with the same body mass index.

Previous studies examining body weight, body mass index, and gestational weight gain in pregnant women with type 2 diabetes differ in terms of design, control groups, definitions of diabetes, timing of measurements, and the way results are presented [8,12,13,14]. In some studies, women with type 2 diabetes were compared with normoglycemic pregnant women, while in others they were compared with women with type 1 diabetes or gestational diabetes [8,12,14]. The heterogeneity of control groups makes uniform interpretation of the available findings difficult. In addition, a substantial proportion of the literature does not clearly report diagnostic criteria for diabetes, the timing of body weight or body mass index measurement, or the method used to assess gestational weight gain. Furthermore, these findings are mainly derived from observational and retrospective studies, often with small sample sizes, different definitions of diabetes, non-uniform control groups, and heterogeneous reporting of results [12,13,14].

The aim of this study was to systematically review and quantitatively synthesize the available data on body weight-related parameters in pregnant women with type 2 diabetes mellitus, including body weight before pregnancy, body weight during pregnancy, pre-pregnancy body mass index, and gestational weight gain, and to compare these parameters with appropriate control groups, including normoglycemic pregnant women, pregnant women with type 1 diabetes, and pregnant women with gestational diabetes. In addition, metabolic parameters, conception and delivery characteristics, and maternal and neonatal perinatal outcomes reported in the included studies were extracted and descriptively summarized to provide a broader clinical context for the interpretation of body weight-related findings.

Although the coexistence of T2DM, overweight or obesity, and adverse pregnancy outcomes is well established, the available literature has not consistently distinguished the anthropometric profile of T2DM pregnancies from that of other clinically relevant pregnancy groups. The originality of the present study lies in the direct quantitative comparison of pre-pregnancy body weight, first-trimester body weight, pre-pregnancy BMI, and gestational weight gain between pregnancies complicated by T2DM and three distinct control groups: normoglycemic pregnancies, pregnancies complicated by T1DM, and pregnancies complicated by GDM. This comparative approach enables a more precise characterization of whether the weight-related burden associated with T2DM is primarily established before conception and in early pregnancy or is reflected in greater gestational weight gain. In addition, the structured mapping of metabolic, maternal, delivery, and neonatal outcomes provides a broader clinical context for the interpretation of the pooled anthropometric findings. Because these parameters are routinely available and potentially modifiable, their comparative synthesis may also help identify distinct periods for weight-related risk reduction across different diabetes phenotypes.

## 2. Materials and Methods

### 2.1. Design of the Study

This research was conducted as a systematic review with meta-analysis, initially registered in PROSPERO (CRD420251131584) and reported in accordance with PRISMA guidelines [15,16], with additional consideration of MOOSE recommendations for observational studies [17].

### 2.2. Inclusion and Exclusion Criteria

Original studies that evaluated body weight-related parameters (body weight, body mass index, and gestational-weight gain), prior to or any time during pregnancy, in pregnant women with T2DM and controls were included. According to the PICOS system, predefined inclusion criteria were as follows: (P) population: all pregnant women; (E) exposure: T2DM; (C) control: non-T2DM; (O) outcome: body weight-related parameters (body weight, body mass index, and gestational-weight gain); (S) study design: observational studies (cross-sectional, case-control, prospective and retrospective cohort, and nested case-control in cohort studies) and experimental studies. Exclusion criteria were also defined and consisted of the following: (1) language: other than English; (2) non-original articles: reviews (narrative, systematic), meta-analysis, case reports, case series, letter to editor, editorials, comments, correspondences, books, book chapters, abstracts, etc.; (3) wrong population: animals, cell lines, and non-pregnant women; (4) no control group or inappropriate control group; and (5) wrong outcome: other than body weight-related parameters.

### 2.3. Search Strategy

As recommended, two experienced researchers in conducting systematic reviews and meta-analyses (KI, AC) developed and performed the search. Three electronic databases were searched: PubMed, Web of Science (WoS), and SCOPUS until 19th August 2025. Applied search queries within each of databases are presented in Table 1. In addition, the reference lists of reviews and editorials identified through electronic search were manually screened to identify potentially relevant studies.

### 2.4. Article Screening and Selection

Title and abstract reading in the first step and full-text reading in the second step were performed by two independent reviewers (KI, MM). Any discrepancies were resolved through discussion at each stage, with the involvement of a third reviewer when necessary (AC/MGD). The initial phase of study selection was conducted using the Rayyan web-based application. Articles were advanced to full-text screening if they were considered potentially eligible or when the title and abstract did not provide sufficient information for exclusion.

### 2.5. Data Extraction, Risk of Bias, and Quality Assessment

Two reviewers (KI, MM) independently abstracted following data according to previously designed protocol: (1) study characteristics: first author, publication year, country, and study design; (2) characteristics of study groups: sample sizes, cases and controls characteristics, age, T2DM definition and criteria, reasons for exclusion (hypertension, cardiovascular disease, renal disease, thyroid disease, endocrine disease, respiratory disease, psychiatric illness or use of psychiatric medication, inflammatory disease, rheumatology disease, immunologic disease, benign/malignant tumor, alcohol/drug abuse, smoking, family history of T1DM, T2DM or GDM, multifetal pregnancy), and matching; (3) body weight-related parameters: body weight, body mass index, gestational weight gain, and nutritional status; (4) metabolic parameters: glycaemia, HbA1c, HOMA-IR, insulin, triglycerides, total cholesterol, LDL, HDL, VLDL, and Tg/HDL ratio; (5) neonatal outcomes: premature delivery, fetal/neonatal loss, congenital abnormalities, large for gestational age, macrosomia, injury, sepsis, NICU stay, respiratory distress syndrome, neonatal hypoglycemia, hypocalcemia, hyperbilirubinemia, and polycythemia; (6) maternal outcomes: miscarriage, PIH, PE, HELLP, polyhydramnion, oligohydramnion, and postpartum hemorrhage; (7) delivery characteristics: type of delivery, maternal age at delivery, gestational age at delivery; and (8) newborn characteristics: gender, neonatal weight, neonatal height, and Apgar score at the 1st and 5th minute. Unavailable articles and/or relevant data were retrieved from the authors. Each reviewer independently performed risk of bias and quality assessment of included articles using an adapted version of the Newcastle−Ottawa tool (NOS) for observational [18] and Jadad scale for experimental studies [19]. Results of quality assessment are given in Supplementary Materials Table S1.

### 2.6. Statistical Analysis

The primary outcome of this meta-analysis was assessing the difference in body weight-related parameters (body weight, body mass index, and gestational weight gain) between T2DM and non-T2DM pregnancies (normoglycemic, T1DM and GDM controls). Thus, standardized mean difference (SMD) was an adequate measure of the overall effect size across studies examining the difference in evaluated parameters between study groups. SMD represents the difference between group means expressed in units of the pooled standard deviation. Summary effect sizes were calculated using the DerSimonian–Laird random-effects model, while fixed-effect estimates were obtained using the inverse-variance method. Chi-square Q and I2 statistic were used to assess the heterogeneity and its scale was defined according to the Cochrane Handbook as low, moderate, and high if I2 was <30%, 30–60%, and >60%, respectively [20]. Each outcome was presented as one forest plot showing the SMD (box), 95% confidence interval of the SMD (lines), and weight (size of box) for each study, as well as the overall effect size presented as a diamond. Publication bias was assessed by funnel plots for each defined outcome (Supplementary Material Figures S1–S10). Statistical significance was defined as a *p*-value of≤ 0.05. Analyses were performed using Review Manager Version 5.4.

When data of relevant parameters were presented in figures only, GetDataGraph Digitizer version 2.26 was applied to read their values. In order to use data as arithmetic means and standard deviations, we applied next approximations: (1) median was used as a direct approximation of the mean, (2) arithmetic mean was calculated as (sd*z) where sd = se*√n if z score was available, (3) standard deviation was calculated as sd = IQR/1.35 if IQR was available, (4) standard deviation was calculated as sd = se*√n in case when standard error (se) was used to present variability, (5) standard deviation instead of range was applied and calculated as sd = (max − min)/4, (6) if 95% CI was used, sd was calculated as ((Upper limit of 95%CI − Lower limit of 95%CI)/3.92)*√n [21], and (7) if the 10th and 90th percentiles were reported, sd was approximated according to (90th percentile − 10th percentile)/2.56.

## 3. Results

**Figure 1:**
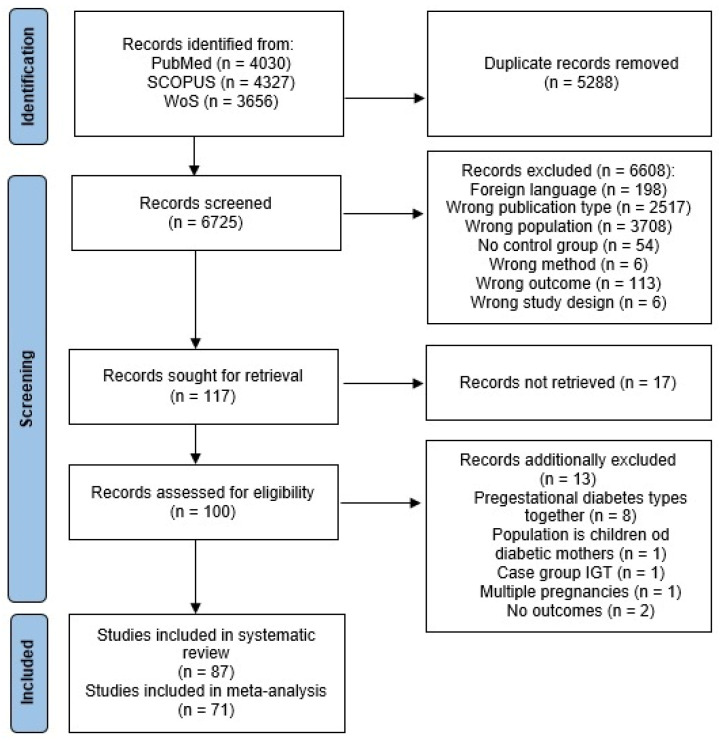
Flow diagram.

A total of 12,013 articles were identified through electronic database searches of PubMed, Scopus, and Web of Science. After removing 5288 duplicates, 6725 articles remained for title and abstract reading. Based on predefined exclusion criteria, 6608 articles were excluded, leaving 117 articles for full-text assessment. Of these, 17 articles could not be retrieved, and the remaining articles were assessed for eligibility based on the full-text review. Finally, 87 articles were included in the systematic review and 72 of them were included in the meta-analysis. A flow diagram showing the process of selection is presented in Figure 1.

### 3.1. Systematic Review

#### Study Characteristics

Studies were published between 1991 and 2025, with a total of 5,426,663 participants, 25,576 pregnant women with T2DM, and 5,401,185 without T2DM (5,148,078 normoglycemic evaluated in 36 studies, 53,607 with T1DM evaluated in 57 studies, 199,392 with GDM evaluated in 28 studies, 84 with overt DM evaluated in two studies, 60 with DIP evaluated in one study, and 24 with T1DM and MODY evaluated in one study). The smallest and the largest T2DM groups had 8 and 6060 cases, respectively. Normoglycemic, T1DM, GDM, overt DM, and MODY DM control groups had the following smallest and largest sample sizes: 8 and 2,340,547, respectively, 5 and 27,075, respectively, 21 and 130,980, respectively, 18 and 207, respectively, and 3 and 7, respectively. Studies were mostly originated from European countries 33/87 (6 from United Kingdom, 4 from Ireland, Spain and Italy, 2 from Romania, Sweden, Germany, and Denmark, and 1 from Poland, Austria, Netherlands, France, Finland, Greece, and Norway). There were 16/87 studies from Asia (6 from Japan, 3 from Russia, 2 from South Korea, and China, and 1 from India, Qatar, and United Arab Emirates), 13/87 from Australia and New Zealand, 11/87 from North America (United States of America and Canada), 9/87 from South America (4 from Brazil, 3 from Chile, and 2 from Mexico), and 5/87 from Africa (4 from South Africa and 1 from Tunis). Seventy-three included studies reported their design. Most were retrospective cohort studies (16/73), while other study designs were prospective cohorts (10/73), case-controls (4/73), and cross-sectional studies (2/73). However, 55% of included studies reported their design unclearly or ambiguously. Maternal age was reported in almost all included studies (80/87). The criteria for diagnosing diabetes mellitus and the applied definition were not reported in even 72% (63/87) and 54% (47/87) of all included studies, respectively. Within the studies that reported the applied definition for T2DM IADPSG criteria were commonly used (12 studies), then ADA in 11 studies, WHO in 9 studies. Carpenter-Coustan criteria, White classification, Australaisan Diabetes in Pregnant Society 1998 criteria, Japan Diabetes Society guideline were used in two studies each, while NICE, UK, ACOG, and Chinese Diabetes Society guidelines were applied in one study each. All cases were T2DM pregnant women, while controls were normoglycemic, T1DM, GDM, overt DM, or MODY DM pregnant women. All characteristics of included studies are provided in Supplementary Material Table S2.

Reasons for exclusion are listed in Supplementary Table S3. A commonly applied reason for exclusion in included studies was multiple pregnancy (35/87). Congenital malformations were exclusion criteria in 7/87 and smoking habit in 5/87 included studies. Other reasons were rarely applied: drug abuse in 3, PCOS in 2, HTA in 2, CVD, renal, and liver diseases in 1 study each. Conception and delivery characteristics are presented in Supplementary Table S2b. The type of conception was reported in 27/87 studies (spontaneous in 26 and IVF in 1). Type of delivery was reported in 62/87. Gestational age at delivery was recorded in 67/87 studies. Reported metabolic parameters in cases and controls are summarized in Supplementary Table S4. Among extracted glucose metabolism parameters, HbA1c was commonly evaluated and reported in included studies (66/87). Glycemia was analyzed in 14, insulin was analyzed in 8, and HOMA-IR index was analyzed in 5 included studies. Only six studies reported some of lipid metabolism parameters (TG, TC, LDL, HDL, or Tg/HDL ratio), while VLDL was not reported in any study. Finally, we collected data regarding neonatal and maternal adverse outcomes, which are summarized in Supplementary Tables S5a and S5b, respectively. Preterm delivery was the most frequently detected perinatal adverse outcome in T2DM group (in 64 out of 87 studies). Fetal/neonatal loss was reported approximately half as often as preterm delivery (in 32 out of 87 studies), as well as congenital malformations (in 34 out of 87 studies). LGA was present in 51/87, SGA was present in 41/87, and macrosomia was present in 40/87 T2DM study groups. IUFD was present in 20/87, injury was present in 11/87, and sepsis was present in 5/87 T2DM study groups. The most commonly reported maternal adverse outcomes were PE and PIH in 42 and 34 T2DM study groups, respectively (Supplementary Table S5b).

### 3.2. Meta-Analysis

Meta-analysis was performed for body weight, pre-pregnancy BMI, and GWG, comparing T2DM with normoglycemic, T1DM, and GDM pregnancies (Table 2).

**Figure 2:**
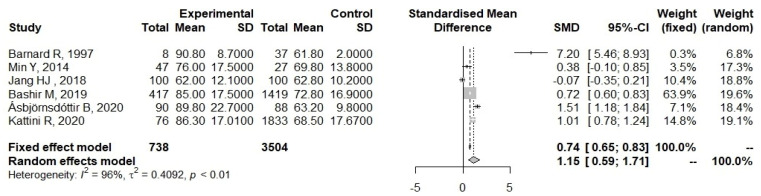
Body weight before pregnancy between T2DM and normoglycemic pregnant women. Studies included: Barnard et al. [22], Min et al. [23], Jang et al. [24], Bashir et al. [8], Ásbjörnsdóttir et al. [13], and Kattini et al. [25]. Squares represent the standardized mean difference (SMD) for individual studies, with the size of each square proportional to the study weight. Horizontal lines indicate 95% confidence intervals (95% CI). The diamond represents the pooled effect estimate, and the vertical line indicates no effect (SMD = 0).

**Figure 3:**
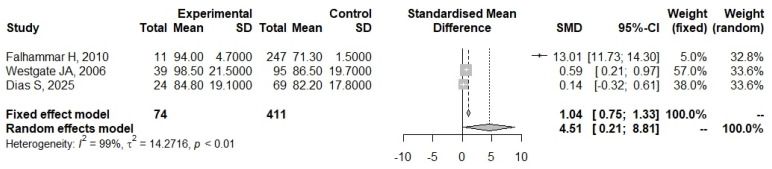
Body weight during the 1st trimester of pregnancy between T2DM and normoglycemic pregnant women. Studies included: Falhammar et al. [26], Westgate et al. [27], and Dias et al. [28]. Symbols are the same as in Figure 2.

**Figure 4:**
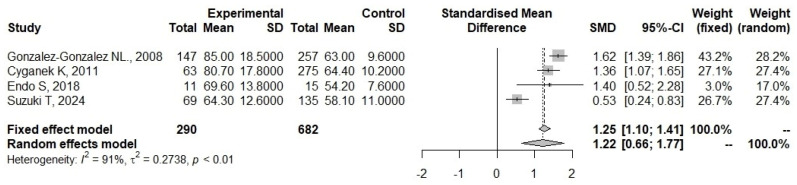
Body weight before pregnancy between T2DM and T1DM pregnant women. Studies included: Gonzalez-Gonzalez et al. [29], Cyganek et al. [30], Endo et al. [31], and Suzuki et al. [32]. Symbols are the same as in Figure 2.

**Figure 5:**
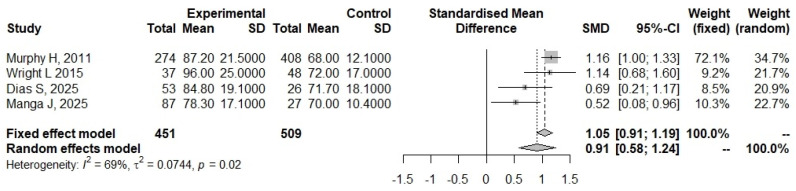
Body weight during the 1st trimester of pregnancy between T2DM and T1DM pregnant women. Studies included: Murphy et al. [33], Wright et al. [34], Dias et al. [28], and Manga et al. [35]. Symbols are the same as in Figure 2.

**Figure 6:**
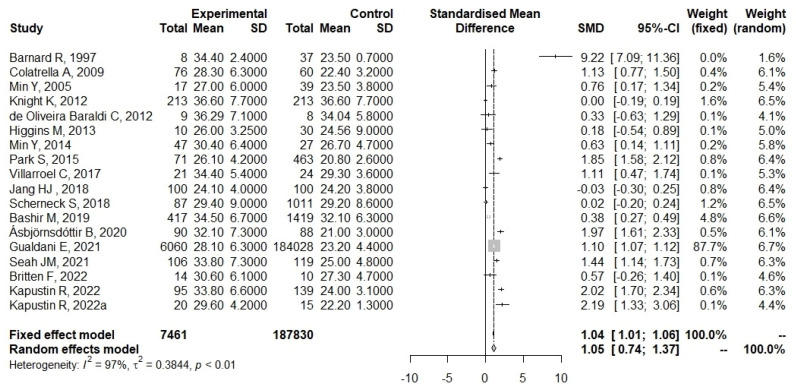
Pre-pregnancy BMI between T2DM and normoglycemic pregnant women. Studies included: Barnard et al. [22], Colatrella et al. [36], Min et al. [23], Knight et al. [37], de Oliveira Baraldi et al. [38], Higgins et al. [39], Min et al. [40], Park et al. [41], Villarroel et al. [42], Jang et al. [24], Scherneck et al. [43], Bashir et al. [8], Ásbjörnsdóttir et al. [13], Gualdani et al. [44], Seah et al. [45], Britten et al. [46], and Kapustin et al. [47,48,49]. Symbols are the same as in Figure 2.

**Figure 7:**
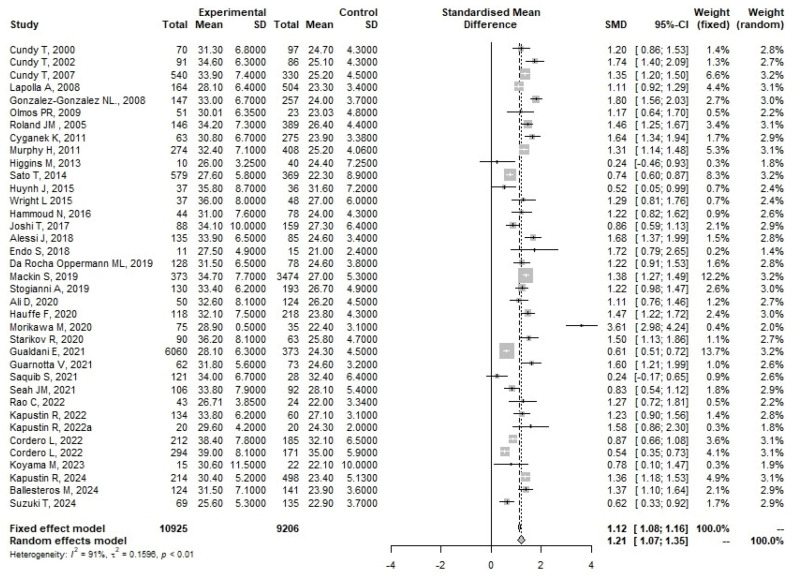
Pre-pregnancy BMI between T2DM and T1DM pregnant women. Studies included: Cundy et al. [50,51,52], Lapolla et al. [53], Gonzalez-Gonzalez et al. [29], Olmos et al. [54], Roland et al. [55], Cyganek et al. [30], Murphy et al. [33], Higgins et al. [39], Sato et al. [56], Huynh et al. [57], Wright et al. [34], Hammoud et al. [58], Joshi et al. [59], Alessi et al. [60], Endo et al. [31], Da Rocha Oppermann et al. [61], Mackin et al. [62], Stogianni et al. [63], Ali et al. [64], Hauffe et al. [65], Morikawa et al. [66], Starikov et al. [67], Gualdani et al. [44], Guarnotta et al. [68], Saquib et al. [69], Seah et al. [45], Rao et al. [70], Kapustin et al. [47,48,49], Cordero et al. [71,72], Koyama et al. [73], Ballesteros et al. [74], and Suzuki et al. [32]. Symbols and lines are defined as in Figure 2.

**Figure 8:**
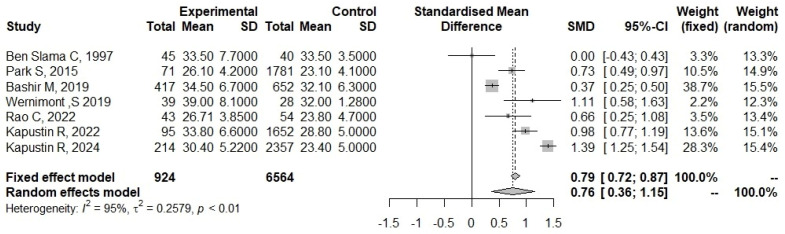
Pre-pregnancy BMI between T2DM and GDM pregnant women. Studies included: Ben Slama et al. [75], Park et al. [41], Bashir et al. [8], Wernimont et al. [76], Rao et al. [70], and Kapustin et al. [47,49]. Symbols and lines are defined as in Figure 2.

**Figure 9:**
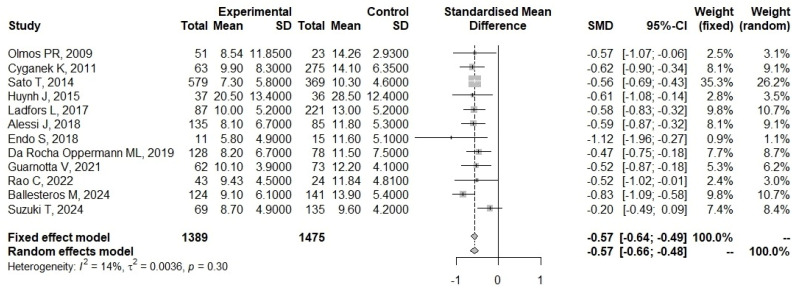
GWG between T2DM and T1DM pregnant women. Studies included: Olmos et al. [54], Cyganek et al. [30], Sato et al. [56], Huynh et al. [57], Ladfors et al. [77], Alessi et al. [60], Endo et al. [31], Da Rocha Oppermann et al. [61], Guarnotta et al. [68], Rao et al. [70], Ballesteros et al. [74], and Suzuki et al. [32]. Symbols and lines are defined as in Figure 2.

**Figure 10:**
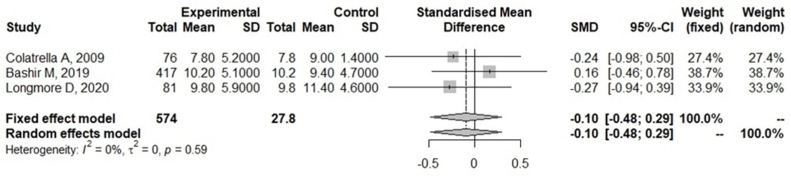
GWG between T2DM and normoglycemic pregnant women. Studies included: Colatrella et al. [36], Bashir et al. [8], and Longmore et al. [78]. Symbols and lines are defined as in Figure 2.

**Figure 11:**
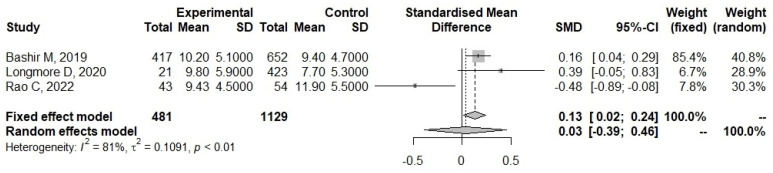
GWG between T2DM and GDM pregnant women. Studies included: Bashir et al. [8], Longmore et al. [78], and Rao et al. [70]. Symbols and lines are defined as in Figure 2.

Body weight measured before pregnancy and in the first trimester was significantly higher in T2DM than in normoglycemic pregnancies (SMD = 1.151, 95% CI SMD = 0.594–1.708, *p* < 0.001 and SMD = 4.508, 95% CI SMD = 0.208–8.809, *p* = 0.040, respectively) (Figure 2 and Figure 3) and T1DM pregnancies (SMD = 1.215, 95% CI SMD = 0.657–1.773, *p* < 0.001 and SMD = 0.913, 95% CI SMD = 0.583–1.242, *p* < 0.001) (Figure 4 and Figure 5). Pre-pregnancy BMI was also significantly higher in T2DM than in normoglycemic, T1DM, and GDM pregnancies (SMD = 1.055, 95% CI SMD = 0.741–1.369, *p* < 0.001, SMD = 1.211, 95% CI SMD = 1.070–1.353, *p* < 0.001, and SMD = 0.755, 95% CI SMD = 0.360–1.150, *p* < 0.001, respectively) (Figure 6, Figure 7 and Figure 8). Finally, GWG was significantly lower in T2DM than in T1DM (SMD = −0.566, 95% CI SMD = −0.645 to −0.487, *p* < 0.001), while no significant difference was observed between T2DM and normoglycemic or GDM pregnancies (SMD = −0.098, 95% CI SMD = −0.484–0.289, *p* = 0.621 and SMD = −0.035, 95% CI SMD = −0.386–0.456, *p* = 0.871, respectively) (Figure 9, Figure 10 and Figure 11).

## 4. Discussion

The results of our systematic review and meta-analysis indicate that pregnancies complicated by type 2 diabetes mellitus are characterized by significantly less favorable body weight-related parameters and body mass index before pregnancy and in early pregnancy compared with those in the corresponding control groups. Pre-pregnancy body weight, first-trimester body weight, and pre-pregnancy body mass index were significantly higher in pregnant women with type 2 diabetes than in normoglycemic pregnant women, but also higher than in pregnant women with type 1 diabetes. In addition, pre-pregnancy body mass index was significantly higher in comparison with pregnant women with gestational diabetes. In contrast, gestational weight gain did not differ significantly between pregnant women with type 2 diabetes and normoglycemic pregnant women, nor between pregnant women with type 2 diabetes and those with gestational diabetes. Moreover, gestational weight gain was significantly lower in pregnant women with type 2 diabetes compared with that in pregnant women with type 1 diabetes.

The results of this meta-analysis emphasize that metabolic differences in pregnant women with type 2 diabetes are established before pregnancy and are already present in the early weeks of gestation. Increased body weight before conception, higher first-trimester body weight, and increased pre-pregnancy body mass index reflect the underlying pathophysiology of type 2 diabetes, which is characterized by chronic insulin resistance, β-cell dysfunction, central adiposity, dyslipidemia, and frequently associated cardiometabolic risk factors [1,3,5]. Therefore, pregnancy complicated by type 2 diabetes does not begin from a metabolically favorable state, but from a pre-existing unfavorable metabolic environment, onto which the physiological metabolic changes of pregnancy are superimposed [6,7]. In this context, body weight and pre-pregnancy BMI should not be regarded merely as descriptive characteristics of the study population, but as important indicators of preconception metabolic risk.

The contribution of the present study should therefore be interpreted as a quantitative refinement and consolidation of established clinical knowledge rather than as the identification of a previously unknown association between T2DM and increased body weight. By separately analyzing several anthropometric parameters and clinically distinct control groups, the present meta-analysis demonstrates that the distinguishing weight-related characteristic of T2DM pregnancy is predominantly an unfavorable preconception and early-pregnancy anthropometric profile, whereas total gestational weight gain is not uniformly increased. This distinction is clinically relevant because it shifts the emphasis from antenatal weight gain alone toward metabolic and weight-related risk modification before conception.

Significantly higher body weight and higher pre-pregnancy BMI in pregnant women with type 2 diabetes compared with those in normoglycemic pregnant women were expected, given the close association between type 2 diabetes, overweight, and obesity [3,6]. In the present meta-analysis, higher pre-pregnancy body weight, first-trimester body weight, and pre-pregnancy BMI in women with type 2 diabetes compared with those in normoglycemic pregnant women were confirmed across several included studies [8,13,22,23,24,25,26,27,28,36,37,38,39,40,41,42,43,44,45,46,47,48,49]. These findings indicate that increased body weight is already present before conception and persists in early pregnancy, underscoring the importance of preconception assessment of metabolic risk.

Of particular importance is the finding that pre-pregnancy body weight, first-trimester body weight, and pre-pregnancy BMI were also higher in women with type 2 diabetes than in women with type 1 diabetes, a pattern consistently observed across multiple studies included in the quantitative synthesis [28,29,30,31,32,33,34,35,39,47,48,49,50,51,52,53,54,55,56,57,58,59,60,61,62,63,64,65,66,67,68,69,70,71,72,73,74]. This finding highlights the distinction between the two conditions: type 1 diabetes is primarily characterized by autoimmune β-cell destruction and absolute insulin deficiency, whereas type 2 diabetes most commonly develops in the context of insulin resistance, central adiposity, and metabolic syndrome [1,5]. Therefore, pregnant women with type 2 diabetes should not be viewed only as a population with pre-existing dysglycemia, but as a group with a broader cardiometabolic burden, which is relevant for preconception assessment and antenatal risk stratification.

The finding that pre-pregnancy BMI was higher in pregnant women with type 2 diabetes compared with that in pregnant women with gestational diabetes is also clinically important. Both groups are associated with insulin resistance, but they differ in the timing of onset and duration of the metabolic disorder [4,5]. In gestational diabetes, dysglycemia most commonly becomes evident only when the physiological insulin resistance of pregnancy exceeds the compensatory capacity of β-cells, whereas in type 2 diabetes, insulin resistance, β-cell dysfunction, and frequently associated obesity precede pregnancy. In the present meta-analysis, the higher pre-pregnancy BMI in women with type 2 diabetes compared with women with gestational diabetes was confirmed across studies directly comparing these groups [8,41,47,49,70,75,76]. Therefore, women with type 2 diabetes probably represent a more metabolically burdened phenotype than women with gestational diabetes, as reflected by their significantly higher pre-pregnancy BMI.

The results regarding gestational weight gain show a different pattern compared with pre-pregnancy body weight and BMI. Although pregnant women with type 2 diabetes enter pregnancy with a higher body weight and a more pronounced preconception metabolic burden, their total gestational weight gain was not significantly greater than that of normoglycemic pregnant women or pregnant women with gestational diabetes. In contrast, the difference in gestational weight gain between type 2 diabetes and type 1 diabetes suggests that weight dynamics during pregnancy depend not only on the presence of diabetes, but also on diabetes type, baseline BMI, and treatment model [8,30,31,32,36,54,56,57,60,61,68,70,74,77,78]. These findings should not be interpreted as evidence of similar metabolic risk between groups, but rather as an indication that gestational weight gain depends on a broader clinical context, including baseline BMI, timing of onset of dysglycemia, intensity of antenatal surveillance, lifestyle and dietary measures, and therapeutic approach.

In pregnant women with type 2 diabetes, the metabolic disorder precedes conception, but these patients are most often recognized as high-risk from the beginning of pregnancy and are included in regular glycemic monitoring, lifestyle and dietary counseling, and, when needed, appropriate treatment, including insulin, metformin, or a combined therapeutic approach. The interpretation of gestational weight gain is further complicated by heterogeneous therapeutic approaches across the included studies. Some women with type 2 diabetes were managed with lifestyle and dietary measures and/or oral antidiabetic agents, whereas many were treated with insulin or combination therapy during pregnancy [43,47,48,49,70,76,78]. In contrast, gestational diabetes is most commonly diagnosed later in pregnancy, and therapeutic interventions are generally introduced only after diagnosis [4]. Consequently, a similar total gestational weight gain between type 2 diabetes, gestational diabetes, and normoglycemic pregnancies may arise from different clinical circumstances and does not indicate metabolic equivalence between these groups.

The significantly lower gestational weight gain in pregnant women with type 2 diabetes compared with that in pregnant women with type 1 diabetes further supports the need to interpret weight gain in relation to the underlying metabolic phenotype. Pregnant women with type 2 diabetes most often enter pregnancy with a higher BMI, which is why clinical care more often aims for carefully controlled and sometimes more restricted gestational weight gain [11,13,14]. In contrast, in pregnant women with type 1 diabetes, particularly those receiving intensive insulin therapies, body weight changes may be influenced by different insulin therapy dynamics, adjustment of carbohydrate intake, and prevention of hypoglycemia [2,5]. Therefore, the difference in gestational weight gain between type 2 and type 1 diabetes pregnancies likely reflects the combined influence of baseline BMI, nutritional recommendations, therapeutic approach, and the underlying pathophysiology of the disease.

Taken together, these contrasting patterns identify distinct and potentially modifiable periods for weight-related care across diabetes phenotypes. In women with T2DM, the consistently higher pre-pregnancy body weight and BMI compared with the clinically relevant control groups place particular emphasis on weight optimization before conception. Conversely, the higher gestational weight gain observed in women with T1DM relative to those with T2DM supports careful and individualized monitoring of gestational weight trajectories during pregnancy. However, this finding should not be interpreted as evidence that gestational weight gain was necessarily excessive in women with T1DM, because the included studies did not uniformly classify weight gain according to pre-pregnancy BMI-specific recommendations or directly evaluate whether the between-group difference was associated with adverse outcomes.

These results indicate that pre-pregnancy BMI and gestational weight gain in pregnant women with type 2 diabetes should be interpreted together, rather than as separate parameters. High pre-pregnancy body weight reflects the underlying preconception metabolic risk, whereas gestational weight gain may be influenced by the intensity of antenatal surveillance, lifestyle and dietary measures, therapeutic approach, and degree of glycemic control. Therefore, the same amount of gestational weight gain may not have the same clinical significance in a normoglycemic pregnant woman with normal BMI and in a pregnant woman with type 2 diabetes and obesity. This interpretation is consistent with studies suggesting that controlled or more restricted gestational weight gain in obese pregnant women with type 2 diabetes may be associated with a more favorable perinatal profile, without clear evidence of an increased risk of small-for-gestational-age neonates [13,14].

The clinical implication of these findings is that body weight optimization in women with type 2 diabetes should begin before pregnancy. Since differences in body weight and BMI are already present before pregnancy and in the first trimester, interventions initiated only later in pregnancy are unlikely to fully neutralize the pre-existing metabolic burden. Preconception counseling should include assessment of BMI, nutritional status, quality of glycemic control, therapeutic regimen, and the presence of hypertension, dyslipidemia, and other cardio metabolic risk factors [2,5,8]. At the same time, control of gestational weight gain remains an important element of antenatal surveillance, although optimal values for pregnant women with type 2 diabetes are still not clearly defined, particularly in relation to pre-pregnancy BMI categories, therapeutic approach, and degree of glycemic control [11,13,14].

Importantly, preconception weight optimization should not be considered in isolation, but as one component of comprehensive preconception care for women with pre-existing T2DM. These patients should receive structured counseling regarding the increased maternal and fetal risks associated with diabetes in pregnancy. Priorities before conception should include optimization of glycemic control, assessment and appropriate management of hypertension, and review of glucose-lowering, antihypertensive, and other medications with respect to their suitability for pregnancy. Preconception assessment should also include screening for diabetic retinopathy and nephropathy, including evaluation of albuminuria and renal function, as well as assessment for peripheral or autonomic neuropathy where appropriate. Therefore, body weight management should be integrated into multidisciplinary preconception care involving both diabetes and obstetric care teams [79].

It is important to emphasize that, in this systematic review, metabolic parameters as well as maternal and neonatal perinatal outcomes were also collected and descriptively summarized. Among glucose metabolism parameters, HbA1c was the most frequently reported and was recorded in 66 of the 87 included studies, while glycemia, insulin, and the HOMA-IR index were analyzed much less frequently. Lipid metabolism parameters were reported in only a small number of studies, indicating that the role of lipids in the metabolic profile of pregnancies complicated by type 2 diabetes remains insufficiently investigated. In addition, the collected data indicate that preterm delivery, LGA, SGA, macrosomia, fetal/neonatal loss, and congenital anomalies were the most frequently recorded outcomes in type 2 diabetes groups, while preeclampsia and pregnancy-induced hypertension were the most commonly reported maternal complications. However, these outcomes were not quantitatively pooled in this meta-analysis; therefore, the findings on body weight, BMI, and gestational weight gain cannot be directly interpreted as evidence of their association with specific maternal or neonatal complications.

A strength of this study lies in the large number of included studies and the possibility of comparing pregnant women with type 2 diabetes with several relevant control groups, including normoglycemic pregnant women, pregnant women with type 1 diabetes, and pregnant women with gestational diabetes. This enabled the specific features of type 2 diabetes in pregnancy to be assessed in relation to populations that differ in pathophysiology, timing of onset of dysglycemia, and baseline metabolic profile. An additional value of the study is its focus on parameters that are easily available in clinical practice and can be incorporated into early metabolic risk assessment. Furthermore, the broader descriptive mapping of metabolic parameters, delivery characteristics, and maternal and neonatal outcomes across the included studies adds further value.

Nevertheless, this study has several limitations. The most important limitation is the heterogeneity of the included studies in terms of design, sample size, population, diagnostic criteria for diabetes, and characteristics of control groups, timing of measurements, and reporting of body weight-related parameters. In a substantial number of studies, diagnostic criteria for diabetes were not clearly reported, which may have affected the accuracy of group classification. In addition, the data were mostly derived from observational and retrospective studies, limiting the ability to draw causal conclusions. Further methodological uncertainty arises from inconsistent reporting, the need to recalculate or approximate some data, and the inability to reliably analyze findings according to BMI categories, type of therapy, degree of glycemic control, and duration of diabetes. These factors are potentially important for understanding the relationship between body weight, gestational weight gain, and the course of pregnancy, but they were not sufficiently uniformly reported in the available literature.

Future research should focus on prospective, methodologically standardized studies that monitor pre-pregnancy body weight, pre-pregnancy BMI, gestational weight gain, therapeutic approach, and glycemic control in women with type 2 diabetes. It is particularly important to analyze gestational weight gain in relation to baseline BMI and type of therapy, because the same amount of weight gain may have different implications in women with different metabolic profiles. Future studies should also enable quantitative assessment of the association between body weight-related parameters and maternal and neonatal outcomes, since these outcomes are of great importance as indicators of pregnancy success, frequently reported but inconsistently defined. Future studies should additionally investigate potential dose–response relationships between body weight-related parameters, and maternal and neonatal outcomes in women with T2DM to indicate the strength of the association, whereby greater pre-pregnancy weight, pre-pregnancy BMI, and gestational weight gain are associated with more frequent and/or more severe adverse pregnancy outcomes. Moreover, adequately powered prospective studies and randomized controlled trials are needed to determine whether structured preconception and antenatal lifestyle interventions, including individualized nutritional counseling, physical activity, and weight-management strategies, can safely improve maternal weight trajectories, glycemic control, and perinatal outcomes.

## 5. Conclusions

Pregnancies complicated by type 2 diabetes mellitus are characterized by a less favorable preconception anthropometric and metabolic profile compared with those in the corresponding control groups. In this meta-analysis, pregnant women with type 2 diabetes had significantly greater pre-pregnancy body weight, first-trimester body weight, and pre-pregnancy body mass index than normoglycemic pregnant women and those with type 1 diabetes, while pre-pregnancy body mass index was also higher than in women with gestational diabetes. In contrast, gestational weight gain followed a different pattern: it was not significantly increased compared with normoglycemic pregnancies or pregnancies complicated by gestational diabetes, and was lower than in pregnancies complicated by type 1 diabetes.

These findings indicate that the metabolic risk in women with type 2 diabetes is largely established before conception, whereas gestational weight gain reflects a more complex interplay between baseline BMI, therapeutic approach, glycemic control, and the intensity of antenatal surveillance. These contrasting patterns suggest different periods for weight-related intervention: preconception weight optimization is particularly relevant in women with T2DM, whereas the higher gestational weight gain observed in T1DM relative to T2DM highlights the need for careful and individualized monitoring of gestational weight trajectories during pregnancy. Therefore, preconception optimization of body weight, early assessment of metabolic risk, and individualized monitoring of gestational weight gain should be considered important components of care for women with type 2 diabetes who are planning pregnancy or are already pregnant. Preconception weight optimization should not be viewed as an isolated measure, but should be integrated into comprehensive preconception care that includes counseling regarding maternal and fetal risks, optimization of glycemic control, assessment and management of blood pressure, review of therapy, and screening for retinopathy, nephropathy, and neuropathy. Future prospective studies are needed to define more precise recommendations for optimal gestational weight gain in this high-risk population and to clarify its relationship with maternal and neonatal outcomes.

## Figures and Tables

**Table 1:** Search strategies.

Database	Search Query
PubMed	(Diabetes Mellitus, Type 2 OR Diabetes Mellitus, Adult-Onset OR Diabetes Mellitus, Type II OR Type 2 Diabetes OR Type 2 Diabetes Mellitus OR T2DM OR DM2) AND (Pre-pregnancy body weight OR Pre-pregnancy body weight OR Pre-gestational body weight OR Pre-gestational weight OR Pre-pregnancy weight OR Preconception* weight OR Maternal pre-pregnancy weight OR Maternal pre-gestational weight OR BMI OR Body mass index OR Quetelet Index OR Pre-pregnancy BMI OR Pre-pregnancy BMI OR Pre-gestational BMI OR Maternal pre-pregnancy BMI OR Maternal pre-gestational BMI OR Nutrition* level OR Nutrition* state OR Nutrition* status OR Nutrition* condition OR Nutrition* profile OR Body weight status OR Weight status OR BMI category OR BMI class* OR Adiposity status OR Anthropometric status OR Body composition OR Undernutrition* OR Malnutrition* OR Overnutrition* OR Nutritional deficiency OR Nutritional inadequacy OR Nutritional imbalance* OR Obesity status OR Obes* OR Overweight status OR Owerweight*) AND (Pregnan*)
SCOPUS	(TITLE-ABS-KEY( “Diabetes Mellitus, Type 2” ) OR TITLE-ABS-KEY( “Diabetes Mellitus, Adult-Onset” ) OR TITLE-ABS-KEY( “Diabetes Mellitus, Type II” ) OR TITLE-ABS-KEY( “Type 2 Diabetes” ) OR TITLE-ABS-KEY( “Type 2 Diabetes Mellitus” ) OR TITLE-ABS-KEY( “T2DM” ) OR TITLE-ABS-KEY( “DM2” )) AND (TITLE-ABS-KEY( “Pre-pregnancy body weight” ) OR TITLE-ABS-KEY( “Pre-pregnancy body weight” ) OR TITLE-ABS-KEY( “Pre-gestational body weight” ) OR TITLE-ABS-KEY( “Pre-gestational weight” ) OR TITLE-ABS-KEY( “Pre-pregnancy weight” ) OR TITLE-ABS-KEY( “Preconception* weight” ) OR TITLE-ABS-KEY( “Maternal pre-pregnancy weight” ) OR TITLE-ABS-KEY( “Maternal pre-gestational weight” ) OR TITLE-ABS-KEY( “BMI” ) OR TITLE-ABS-KEY( “Body mass index” ) OR TITLE-ABS-KEY( “Quetelet Index” ) OR TITLE-ABS-KEY( “Pre-pregnancy BMI” ) OR TITLE-ABS-KEY( “Pre-pregnancy BMI” ) OR TITLE-ABS-KEY( “Pre-gestational BMI” ) OR TITLE-ABS-KEY( “Maternal pre-pregnancy BMI” ) OR TITLE-ABS-KEY( “Maternal pre-gestational BMI” ) OR TITLE-ABS-KEY( “Nutrition* level” ) OR TITLE-ABS-KEY( “Nutrition* state” ) OR TITLE-ABS-KEY( “Nutrition* status” ) OR TITLE-ABS-KEY( “Nutrition* condition” ) OR TITLE-ABS-KEY( “Nutrition* profile” ) OR TITLE-ABS-KEY( “Body weight status” ) OR TITLE-ABS-KEY( “Weight status” ) OR TITLE-ABS-KEY( “BMI category” ) OR TITLE-ABS-KEY( “BMI class*” ) OR TITLE-ABS-KEY( “Adiposity status” ) OR TITLE-ABS-KEY( “Anthropometric status” ) OR TITLE-ABS-KEY( “Body composition” ) OR TITLE-ABS-KEY( “Undernutrition*” ) OR TITLE-ABS-KEY( “Malnutrition*” ) OR TITLE-ABS-KEY( “Overnutrition*”) OR TITLE-ABS-KEY( “Nutritional deficiency” ) OR TITLE-ABS-KEY( “Nutritional inadequacy” ) OR TITLE-ABS-KEY( “Nutritional imbalance*” ) OR TITLE-ABS-KEY( “Obesity status” ) OR TITLE-ABS-KEY( “Obes*” ) OR TITLE-ABS-KEY( “Overweight status” ) OR TITLE-ABS-KEY( “Owerweight*” )) AND (TITLE-ABS-KEY( “Pregnan*” ))
Web of Science	(TS=( Diabetes Mellitus, Type 2 ) OR TS=( Diabetes Mellitus, Adult-Onset ) OR TS=( Diabetes Mellitus, Type II ) OR TS=( Type 2 Diabetes ) OR TS=( Type 2 Diabetes Mellitus ) OR TS=( T2DM ) OR TS=( DM2 )) AND (TS=( Pre-pregnancy body weight ) OR TS=( Pre-pregnancy body weight ) OR TS=( Pre-gestational body weight ) OR TS=( Pre-gestational weight ) OR TS=( Pre-pregnancy weight ) OR TS=( Preconception* weight ) OR TS=( Maternal pre-pregnancy weight ) OR TS=( Maternal pre-gestational weight ) OR TS=( BMI ) OR TS=( Body mass index ) OR TS=( Quetelet Index ) OR TS=( Pre-pregnancy BMI ) OR TS=( Pre-pregnancy BMI ) OR TS=( Pre-gestational BMI ) OR TS=( Maternal pre-pregnancy BMI ) OR TS=( Maternal pre-gestational BMI ) OR TS=( Nutrition* level ) OR TS=( Nutrition* state ) OR TS=( Nutrition* status ) OR TS=( Nutrition* condition ) OR TS=( Nutrition* profile ) OR TS=( Body weight status ) OR TS=( Weight status ) OR TS=( BMI category ) OR TS=( BMI class* ) OR TS=( Adiposity status ) OR TS=( Anthropometric status ) OR TS=( Body composition ) OR TS=( Undernutrition* ) OR TS=( Malnutrition* ) OR TS=( Overnutrition*) OR TS=( Nutritional deficiency ) OR TS=( Nutritional inadequacy ) OR TS=( Nutritional imbalance* ) OR TS=( Obesity status ) OR TS=( Obes* ) OR TS=( Overweight status ) OR TS=( Owerweight* )) AND (TS=( Pregnan* ))

The asterisk (*) represents a truncation wildcard used in database searches to retrieve all words sharing the same root (e.g., Pregnant* retrieves pregnant, pregnancy; Obes* retrieves obesity, obese).*.

**Table 2:** Results of meta-analysis of body weight, pre-pregnancy BMI, and GWG between T2DM and normoglycemic, T1DM, and GDM pregnancies.

T2DM vs.	Normoglycemic Controls	T1DM	GDM
Pre-pregnancy body weight	↑	↑	NA
1st-trimester body weight	↑	↑	NA
Pre-pregnancy BMI	↑	↑	↑
GWG	Not sig.	↓	Not sig.

↑ indicates a significantly higher value in T2DM compared with the reference group; ↓ indicates a significantly lower value; NA, not available; Not sig., not statistically significant.

## Data Availability

All additional data are available as Supplementary Materials.

## Supplementary Materials

The following supporting information can be downloaded at: https://www.mdpi.com/article/10.3390/jcm15135260/s1, Table S1: Quality assessment of the included studies; Table S2a. Study and study group characteristics; Table S2b. Conception and delivery traits; Table S3: Reasons for exclusion reported in the included studies; Table S4: Metabolic parameters in cases and controls; Table S5a: Neonatal adverse outcomes; Table S5b: Maternal adverse outcomes; Table S6: PRISMA checklist [69]; Figures S1–S10: Funnel plots for the meta-analyses of body weight-related parameters comparing pregnancies complicated by type 2 diabetes mellitus with normoglycemic, type 1 diabetes mellitus, and gestational diabetes control groups.
