# Supplementary material for: Body Weight-Related Parameters in Pregnancies Complicated by Type 2 Diabetes Mellitus: A Systematic Review and Meta-Analysis with Maternal and Perinatal Outcome Mapping
Source: J Clin Med. 2026 Jul 6;15(13):5260. doi: 10.3390/jcm15135260 (PMC13362816; doi:10.3390/jcm15135260)
Supplement: Supplementary file 1 [file jcm-15-05260-s001.zip › Supplementary Table S2a. Study and study group characteristics.pdf]

Table 2b. Conception and delivery traits

Author, year<br>Country	Cases			Controls		
	Type of conception	Type of delivery	Gestational age at delivery	Type of conception	Type of delivery	Gestational age at delivery
Barnard R, 1997<br>Australia,	NR	NR	35.5 ± 1.7	NR	Predominantly term vaginal	NR
Ben Slama C, 1997<br>Tunis	spontaneous	NR	NR	spontaneous	NR	NR
Colatrella A, 2009<br>Italy	spontaneous	Cesarean section 71.4%<br>Vaginal 28.6%.	37.7 ± 2.7	spontaneous	Cesarean section 36.6%<br>Vaginal 63.4%	39.7 ± 2.7
Contreras-Soto J, 1991<br>Mexico	spontaneous	NR	NR	spontaneous	NR	NR
Cundy T, 2000<br>New Zealand	spontaneous	Vaginal 45%<br>Caesarean section 55%	37.1 ± 2.4	spontaneous	Vaginal 38%<br>Caesarean section 62%	36.4 ± 2.9
Cundy T, 2002<br>New Zealand	spontaneous	Caesarean section 63.4%<br>Vaginal: 36.6%	38–39 weeks	spontaneous	Caesarean section 71.1%<br>Vaginal 28.9%	22.2% ≤35 weeks
Cundy T, 2007<br>New Zealand	spontaneous	Caesarean section 53.2%<br>Vaginal 46.8%	NR	spontaneous	Caesarean section 56.2%<br>Vaginal 43.8%	NR
Falhammar H, 2010<br>Australia	NR	Vaginal 82%<br>Cesarean section 18%	39.1 ± 0.4	spontaneous	Vaginal 86%<br>Cesarean section 13%<br>Vacuum 1%	38.9 ± 0.1

Lapolla A, 2008<br>Italy	spontaneous	Cesarean section 69.3%<br>Vaginal 30.7 %	37.4	spontaneous	Cesarean section 73 %<br>Vaginal 27 %	37.2
Marin J 2010,<br>Romania	NR	Cesarean section 57%<br>Vaginal 43%	36.97 ± 2.6	NR	Cesarean section 19%<br>Vaginal 81%	38.55 ± 2.0
Min Y, 2005<br>UK	spontaneous	NR	37.6 ± 1.2	Spontaneous	Vaginal 100%	39.8 ± 1.3
Abrão Szylit N, 2007<br>Brasil	NR	Cesarean section 75.0%<br>Vaginal 22.9%<br>Forceps 2.1%	NR	NR	GDM<br>Cesarean section 64.0%<br>Vaginal 27.2%<br>Forceps 8.8%<br>T1DM<br>Cesarean section (100%)	NR
Gonzalez-Gonzalez<br>NL., 2008,<br>Spain	NR	Vaginal 41.2%<br>Forceps 16.0%<br>Cesarean section 42.8%	38 ± 2.2	NR	Vaginal 26.9%<br>Forceps 25.4%<br>Cesarean section 47.7%	NR
Olmos PR, 2009,<br>Chile	NR	Cesarean section 29.4%<br>Vaginal 70.6%	37.33 ± 0.23	NR	Cesarean section 26.1%<br>Vaginal 73.9%	NR
Roland JM, 2005<br>UK	NR	NR	NR	NR	NR	NR
Westgate JA, 2006,<br>New Zealand	NR	Elective Cesarean section<br>21%<br>Emergency Cesarean section<br>23%<br>Operative vaginal 10%<br>Spontaneous vaginal 46%	38.2 ± 1.2	NR	Elective Cesarean section<br>4%<br>Emergency Cesarean section<br>19%<br>Operative vaginal 4%<br>Spontaneous vaginal 72%	40.3 ± 1.4
Yang X, 2002<br>China	NR	Caesarean section 73.5%<br>Vaginal 26.5%	39.4 ± 1.57	NR	Caesarean section 65.9%<br>Vaginal 34.1%<br>Breech-specific Cesarean<br>section 3.64%	39.8 ± 1.54

Cyganek K, 2011 Poland	NR	Cesarean section 58.7% Vaginal 41.3%	38.1 (95%Ci 37.2-39.0)	NR	Caesarean section: 64.1% Vaginal 35.9%	38.2 (95% CI 37.9–38.5)
Handisurya A, 2011 Australia	IVF: 7.7% (as part of total T2DM group) Spontaneous: remainder	Vaginal 50.0% Cesarean section 50.0%	37.60 ± 2.72	NR	Vaginal 38.9% Cesarean section 61.0%	37.56 ± 2.14
Knight K, 2012 USA	NR	Cesarean section 56.8% Non-repeat primary Cesarean section 30.0% Operative vaginal 4.7%	38.4	NR	Cesarean section 38.5% Operative vaginal 5.6%	NR
Murphy H, 2011 UK	NR	Vaginal + assisted 48.6% Cesarean section 51.4%	38.1 (35.6–39.3)	NR	Vaginal + assisted 36.5% Cesarean section 63.5% Elective 29.7% Emergency 33.7%	37.4 (34.0–38.6)
de Oliveira Baraldi C, 2012 Brasil	NR	Caesarean section 77.7% Vaginal 23.3%	~38.3	NR	Caesarean section 75% Vaginal 25%	~39.7
Higgins M, 2013 Ireland	NR	Cesarean section 70% Vaginal 30%	38+6 (38+0 to 40+0)	NR	Cesarean section 73.3% Vaginal 26.6%	39+0 (37+6–41+0)
Min Y, 2014 United Kingdom	NR	Vaginal (spontaneous) 30.0% Assisted 3.3% Induced 10.0% Elective Cesarean section 33.3% Emergency Cesarean section 23.3%	37.1 (31.0–40.0)	NR	Vaginal spontaneous 63.0% Assisted 7.4% Induced 3.7% Elective Cesarean section 11.1% Emergency Cesarean section 14.8%	39.3 (36.0–42.0)
Sato T, 2014 Japan	NR	Primary cesarean section 28.4% Induced labor 33.3% Other delivery modes not individually specified.	37.7 ± 2.4	NR	Primary cesarean section 21.6% Induced labor 41.5%	37.6 ± 2.3
Hall D, 2015, South Africa	NR	Caesarean section 59.5% Vaginal: 40.5%	37 (25–39)	NR	NR	NR
Huynh J, 2015 USA	NR	NR	38.0 ± 1.9	NR	NR	37.5 ± 2.1

Owens L, 2015<br>Ireland	NR	Elective CS 39 4%<br>Emergency CS 18 2%<br>Vaginal + instrumental 51 5%	Only reported as proportion <37 weeks = 22%	NR	Elective CS 65 3%<br>Emergency CS 92 3%<br>Vaginal + instrumental 58 4%	28% delivered <37 weeks
Park S, 2015<br>South Korea	NR	CS 69.5%<br>Vaginal 30.5%	38.33 ± 1.77	NR	NR	39.04 ± 1.84
Wright L 2015<br>USA	NR	CS 60%<br>Vaginal 40%	37.9 ± 1.2	NR	CS 71%<br>Vaginal 29%	37.0 ± 3.1
Abell S, 2016<br>Australia	NR	CS 53.6%<br>Vaginal 46.4%	38.3 (37.1–39.1).	NR	CS 26.3%<br>Vaginal 73.7%	39.4 (38.4–40.4).
Cade WT , 2016<br>USA	NR	Vaginal 32%<br>CS 68%	37.0 ± 1.9	NR	Vaginal 42%<br>CS 58%	38.8 ± 1.5
Hammoud N, 2016<br>Netherlands	NR	CS: 59% in ODM1 vs 32% in ODM2.	38	NR	NR	37
Villarroel C, 2016,<br>Chile	NR	NR	NR	NR	NR	NR
Billionnet C , 2017<br>France	spontaneous	CS 50.6 %	NR	spontaneous	No diabetes: CS 19.6 %<br>GDM: CS27.8 % CS	NR
Cade WT ,2017<br>USA	spontaneous	Vaginal 36 %<br>CS 64 %	37 ± 2	spontaneous	Vaginal 42 %<br>CS 65 %	38±1
Cnattingius S, 2017<br>Sweden	NR	CS 42.6%<br>Instrumental 5.8%<br>Vaginal 51.6%	85.8% ≥ 37 weeks	spontaneous	Non-DM<br>Vaginal 82.5 %<br>Instrumental 7.0 %<br>CS 10.5 %<br>T1DM<br>CS 50.7%<br>Instrumental 9.8%<br>Vaginal 39.6%	Non-DM 95.1%<br>T1DM 77% ≥ 37 weeks

Joshi T, 2017<br>Australia	NR	Vaginal 36 %<br>CS 63%<br>Elective CS 25%<br>Emergency CS 38%	35.7 ± 3.7		Vaginal 25 %<br>CS 67%<br>Elective CS 28%<br>Emergency CS 39	Vaginal 25 %<br>CS 67%<br>Elective CS<br>28%<br>Emergency CS<br>39
Ladfors L, 2017<br>Sweden	NR	30 (35%) SC<br>57 (65%) vaginal	38.0 ± 2.2	NR	92 (42%) SC<br>129 (58%) vaginal	36.1 ± 2.9
Saikia DM , 2017<br>Assam	NR	NR	NR	NR	NR	NR
Villarroel C, 2017<br>Chile	spontaneous	NR	NR	sponatenus	NR	NR
Alessi J, 2018<br>Brazil	NR	CS 69%<br>Vaginal 31%	36.9 ± 3.4	NR	CS 70%<br>Vaginal 30%	35.8 ± 3.5
Endo S, 2018<br>Japan	NR	Urgent CS 27%	32–40	NR	T1DM<br>Urgent CS 33%	32–40
Jang HJ , 2018<br>South Korea	spontaneous	Vaginal 30%<br>Primary CS 35%<br>Repeat CS 34%.	38.7 ± 1.6	spontaneous	Vaginal 15%<br>Primary CS 18%<br>Repeat CS 24%.	38.7 ± 2.4
Maple-Brown LJ ,<br>2018<br>Australia	spontaneous	CS 67 % Indigenous<br>CS 39–50 % non-Indigenous	37.0–37.7	spontaneous	CS 47–57 % Indigenous<br>CS 38–48 % non-Indigenous	37.9–38.1<br>37.6–38.7
Scherneck S, 2018<br>Germany	spontaneous	NR	38.9 (38–40.1)	spontaneous	NR	39 (38–40.1)

Shimizu I, 2018<br>Japan	NR	CS 43.4 %<br>vaginal/induced 56.6 %	38.5 ± 1.7	NR	CS 57%<br>Vaginal/induced 43%)	38.5 ± 1.7
Agha-Jaffar R , 2019<br>United Kingdom	spontaneous	SVD 25 31.3 %<br>AVD 4 5 %<br>Elective CS 28 36.3 %<br>Emergency CS 22 27.5 %.	NR	spontaneous	SVD 46.3 %<br>AVD 16.3 %<br>Elective CS 16.3 %<br>Emergency CS 21.3 %	NR
Bashir M, 2019<br>Qatar	spontaneous	Vaginal 38 %<br>Cesarean 62.9 % (Primary CS 32.4 %, Emergency CS 30.5 %).	36.8 ± 2.9.	spontaneous<br>spontaneous	Vaginal 65 %<br>Cesarean 35 %<br>Vaginal 46 %<br>Cesarean 54 %<br>(Primary 29.4 %, Emergency 24.6 %).	38.8 ± 2.1<br>37.6 ± 2.7
Ásbjörnsdóttir B, 2019<br>Denmark	spontaneous	NR	10.1 ± 1.3	spontaneous	NR	9.0 ± 1.2 weeks.
Egan A, 2019<br>Republic of Ireland	spontaneous	CS 60.5% (elective 32.6%, emergency 27.9%)<br>vaginal 32.6%, instrumental 2.3%.	38.0 (37.3–40.0)	spontaneous	CS 70.1% (elective 37.4%, emergency 32.7%)<br>vaginal 15.0%<br>instrumental 8.4%.	37.2 (36.0–38.7)
Kong L, 2019<br>Finland	NR	NR	Median ≈ 39 weeks (10.1 % preterm < 37 w).	NR	NR	Median ≈ 40 weeks (5.3 % preterm).<br>Median ≈ 37 weeks (37.1 % preterm < 37 w)
Da Rocha Oppermann ML, 2019<br>Brazil	spontaneous	NR	36.1 ± 3.3	spontaneous	NR	35.5 ± 3.5
Mackin S, 2019<br>United Kingdom	spontaneous	Cesarean 58%<br>vaginal 42%	37.0 ± 2.1	spontaneous	Cesarean 67%<br>vaginal 33%	36.4 ± 2.6
Stogianni A, 2019<br>Greece	spontaneous	Cesarean section 74.5%<br>vaginal 25.5%	37.5 ± 2.3	spontaneous	Cesarean section 68.9%<br>vaginal 31.1%	37.1 ± 2.4

Wang X, 2019<br>USA	Spontaneous	NR	38.7 ± 1.9	Spontaneous	NR	38.9 ± 1.7<br>38.4 ± 2.1
Wernimont,S 2019<br>USA	NR	Cesarean section 54%<br>(primary 33%)<br>vaginal 46%.	36.9 ± 0.6.	NR	GDM A1<br>Cesarean section 46%,<br>vaginal 54%<br>GDM A2<br>Cesarean section 51%,<br>vaginal 49%	38.0 ± 2.1<br>37.5 ± 2.0
Ali D, 2020<br>Ireland	spontaneous	SVD 26%; Elective CS 20%; Emergency CS 18%; Assisted vaginal 8.1%	NR	spontaneous	SVD 23.4%; Elective CS 16%; Emergency CS 32%; Assisted vaginal 7.2%	NR
López-de-Andrés A, 2020<br>Spain	NR	Cesarean section 47.5%<br>Vaginal 52.5%	NR	NR	CS 21.6%, Vaginal 78.4%<br>nonDM<br>CS 56.9%, Vaginal 43.1%<br>T1DM<br>SC 28.9%, Vaginal 71.1%<br>GDM	NR
Ásbjörnsdóttir B, 2020<br>Denmark	spontaneous	47% vaginal, 53% cesarean	37.5 ± 1.6	sponatneous	NR	39.2 ± 1.3
Hauffe F, 2020<br>German	NR	55.9% SC	39 (38, 39)	NR	55.0% SC	38 (37, 40)
Kattini R, 2020<br>Canada	NR	Vaginal 59%<br>Cesarean section 41%	38.2 ± 1.8	NR	Vaginal 67%<br>Cesarean section 33%	38.5 ± 1.3
Longmore D, 2020<br>Australia	NR	2% Indigenous / 14% non-Indigenous Instrumental delivery<br>69% Indigenous / non-Indigenous 43% CS<br>29% Indigenous / non-Indigenous 43% vaginal	Indigenous 37.2 ± 1.4<br>Non-Indigenous 37.6 ± 1.3	NR	GDM: instrumental delivery 7% Indigenous / 10% non-Indigenous<br>SC Indigenous 48% / non-Indigenous 39%<br>Vaginal Indigenous 45% / non-Indigenous 51%<br>Normoglycemic:	GDM:<br>Indigenous 38.2 ± 1.2 / Non-Indigenous 38.7 ± 1.2<br>Normoglycemic<br>:<br>Indigenus 39.3 ±

					instrumental delivery 12% Indigenous / 7% non-Indigenous<br>SC Indigenous 27% / non-Indigenous 29%<br>Vaginal Indigenous 61% / non-Indigenous 64%	1.3 / Non-Indigenous 39.6 ± 1.2
Morikawa M, 2020<br>Japan	NR	Caesarean section: 59.6%<br>Vaginal delivery: 40.4%	37.2 ± 0.3 weeks	NR	Cesarean section: 68.9%<br>Vaginal delivery: 31.1%	36.7 ± 0.4
Starikov R, 2020<br>USA	NR	NR	(T2DM SGA): 37 nedelja (IQR 39–33)<br>(T2DM AGA): 39 nedelja (IQR 39–37)	NR	NR	(T1DM SGA): 36 (37–32)<br>(T1DM AGA): 37 (39–32)
Gualdani E, 2021<br>Italy	NR	Total CS: 48.2%<br><br>Emergency CS: 21.8%	NR	NR	T1DM<br>Total cesarean section: 59%<br>Emergency CS: 27.1%<br>no DM<br>Total CS: 33%<br>Emergency CS: 14.2%	NR
Guarnotta V, 2021<br>Italy	NR	NR	38.3 ± 1.3 weeks	NR	NR	37.1 ± 1.6 weeks
Martínez-Cruz C, 2021<br>Mexico	NR	Cesarean section: 63%<br><br>Vaginal delivery: 37%	33.9 ± 3.9 weeks	NR	T1DM<br>Cesarean: 74%<br>Vaginal: 26%<br>GDM<br>Cesarean: 76.2%<br>Vaginal: 23.8%<br>No DM<br>Cesarean: 71.9%<br>Vaginal: 28.1%	34.1 ± 4.0 weeks T1DM<br>35.1 ± 4.1 weeks GDM<br>No DM: 36.7 ± 3.8 weeks
McLean A, 2021<br>Australia	NR	NR	NR	NR	NR	NR
Saquib S, 2021<br>United Arab Emirates	NR	Vaginal 38%; Cesarean 62% (51% planned, 11% emergency)	36.9 ± 1.9 weeks	NR	Vaginal 36%; Cesarean 62% (42% planned, 17% emergency)	35.8 ± 2.2 weeks

Seah JM, 2021<br>Australia	NR	T2DM<br>Vaginal: 33% Cesarean elective: 46%<br>Cesarean emergency: 21%	35.6 ± 3.5	NR	NoDM<br>Vaginal: 59%<br>Cesarean elective: 28%<br>Cesarean emergency: 13%<br>T1DM<br>Vaginal: 32%<br>Cesarean elective: 42%<br>Cesarean emergency: 26%	38.1 ± 1.6<br>36.3 ± 2.8
Britten F, 2022,<br>Australia	NR	Vaginal: 50%; Cesarean: 50%	38.7 ± 1.1	NR	BMI matched<br>Vaginal: 70%); Cesarean: 30%<br>normal BMI<br>Vaginal: 50% Cesarean: 50%	39.4 ± 1.6<br>39.2 ± 0.9
Rao C, 2022<br>China	81.4% spontaneous	58.1% SC	38.0 (37–39)	91.7% spontaneous<br>77.8% spontaneous	45.8% SC<br>50.0% SC	38.0 (38–39)<br>38.0 (38–39)
Jacobsen D, 2022	NR	NR	38+4	NR	NR	39+0<br>38+2<br>38+6
Kapustin R, 2022<br>Russia	NR	NR	NR	NR	NR	NR
Kapustin R,<br>2022a<br>Russia	NR	NR	NR	NR	NR	NR
Cordero L, 2022<br>USA	NR	40% vaginal<br>35% primary SC<br>25% Repeat cesarean	37.9 ± 1.4	NR	37% vaginal<br>42% primary SC<br>21% Repeat cesarean	37.3 ± 1.6
Cordero L, 2022<br>USA	NR	38% vaginal<br>28% primary SC<br>34% Repeat cesarean	38 (37–39)	NR	35% vaginal<br>37% primary SC<br>29% Repeat cesarean	37 (36–38)

Malaza N, 2023<br>South Africa	NR	Normal vaginal delivery:<br>T2DM 28.1%,<br>Elective CS:<br>T2DM 31.6%,<br>Emergency CS:<br>T2DM 40.4%,	Term >37 nedelja:<br>45.6%	NR	Normal vaginal delivery:<br>noDM 47.1%<br>T1DM 30%,<br>GDM 40.6%<br>Elective CS:<br>T2DM 31.6%,<br>T1DM 20%,<br>noDM 20.6%<br>GDM 31.3%<br>Emergency CS:<br>T2DM 40.4%,<br>T1DM 50%,<br>GDM 28.1%<br>noDM 32.4%	Term >37<br>nedelja: 82.9%<br>Term >37<br>nedelja: 33.3%<br>Term >37<br>nedelja: 58.1%
Koyama M, 2023<br>Japan	NR	CS: 10/15 (66.7%)<br>VD: 5/15 (33.3%)	37 (30–41) nedelja	NR	CS: 11/22 (50.0%) ;<br>Vaginal: 11/22 (50.0%)	38 (37–40)<br>nedelja
Powers Carson J,<br>2024<br>USA	NR	NR	NR	NR	NR	NR
Kapustin R, 2024<br>Russia	IVF 3.3%	Elective Cesarean: 32.2%<br>Emergency Cesarean: 14.0%	37.1 ± 2.1 weeks	IVF 5.6%<br>IVF 2.8%	Elective Cesarean: 23.5%<br>Emergency Cesarean: 14.2%<br>Elective Cesarean: 43.6%<br>Emergency Cesarean: 24.7%	36.7 ± 2.3<br>weeks<br>38.2 ± 2.0<br>weeks
Ballesteros M, 2024<br>Spain	NR	Vaginal delivery (%)<br>50%<br>SC 50%	37.31 ± 3.13	NR	Vaginal delivery (%) 50.4%<br>SC 49.6%	37.78 ± 1.02
Grazia Dalfrà M,<br>2024<br>Italy	NR	NR	37.5 ± 2.5 nedelja	NR	NR	38.3 ± 1.17<br>nedelja
Suzuki T, 2024<br>Japan	NR	49.3% SC	38.5 ± 1.2	NR	44.4% SC	38.0 ± 1.1
Dias S, 2025<br>South Africa	NR	NR	NR	NR	NR	NR

Gherbon A, 2025<br>Romania	spontaneous	57% vaginal<br>43% SC	NR	spontaneous	vaginal<br>Cont 55.43%<br>GDM 45.52%<br>T1DM 70.27%<br>SC<br>Cont 44.57%<br>GDM 54.48%<br>T1DM 29.72%	NR
Manga J, 2025<br>South Africa	NR	NR	37 weeks	NR	NR	37 weeks<br>37 weeks<br>37 weeks
Zhou X, 2025<br>China	NR	NR	NR	NR	NR	NR

Abbreviations: CS – Cesarean section
