# Supplementary material for: Body Weight-Related Parameters in Pregnancies Complicated by Type 2 Diabetes Mellitus: A Systematic Review and Meta-Analysis with Maternal and Perinatal Outcome Mapping
Source: J Clin Med. 2026 Jul 6;15(13):5260. doi: 10.3390/jcm15135260 (PMC13362816; doi:10.3390/jcm15135260)
Supplement: Supplementary file 1 [file jcm-15-05260-s001.zip › Supplementary Table S2b. Conception and delivery traits.pdf]

Table S2a. Study and study group characteristics

**Study characteristics**	**Cases**						**Controls**		
Author, year<br>Country<br>Study design	n	Characteristics	Maternal age	DM criteria	DM definition	Therapy	n	Characteristics	Maternal age
Contreras-Soto J, 1991<br>Mexico<br>NR	118	T2DM middle-class, ethnically homogeneous cohort, managed in a combined medical–obstetric clinic.	NR	GDM: fasting plasma glucose >140 mg/dL on ≥2 occasions (diagnostic); insulin instituted if fasting >100 mg/dL and 2-h postprandial >130 mg/dL despite die	NR	Yes. All women with pre-existing NIDDM switched from oral agents to insulin at first antenatal visit; titrated to fasting <100 mg/dL, 2-h <130 mg/dL, negative urine glucose/ketones, and normalized glycosylated Hb within ~2 months. Diet: ~36 kcal/kg ideal weight (20–25% protein, 45–50% carbohydrate, 30–35% fat; meal fractionation specified)	90	GDM	NR
Barnard R, 1997<br>Australia,<br>NR	8	preexisting NIDD	28.5 ± 1.4	venous plasma glucose ≥ 7.8 mmol/L → positive screen	NR	Diet ± oral hypoglycemics	37	Normoglycemic controls	29.7 ± 0.8
Ben Slama C, 1997<br>Tunis<br>Not clear	45	T2DM pregnant women older than 35 years at time of conception	37.8 ± 3.8	NR	NR	Insulin and diet	40	GDM pregnant women older than 35 years	37.2 ± 4.2

Cundy T, 2000 New Zealand NR	70	T2DM	33.4 ± 5.5	Pre-existing diabetes diagnosed before conception; women classified clinically as T1DM (insulin-dependent) or T2DM (non–insulin-dependent or adult-onset).	WHO	Yes – 88% required insulin during pregnancy; 12% on diet alone	97	T1DM	29.4 ± 5.6
Cundy T, 2002 New Zealand NR	91	T2DM	34 ± 5	Type 1 DM: Ketosis-prone and/or insulin-dependent since diagnosis.<br><br>Type 2 DM: Not ketosis-prone, not insulin-dependent at diagnosis, typically adult-onset.	White classification	Yes – Insulin therapy in 97% of pregnancies (only 5 insulin pre-pregnancy).	86	T1DM	28 ± 6
Cundy T, 2007 New Zealand Not clear	540	T2DM	33.0 ± 5.1	Type 1 diabetes (T1DM): Insulin from diagnosis and/or islet autoimmunity.<br><br>Type 2 diabetes (T2DM): Not ketosis-prone; no prolonged insulin requirement.<br><br>“Newly recognized” diabetes: Diagnosed as GDM in pregnancy but persistent postpartum	WHO	Insulin in pregnancy 97%	330	T1DM	29.2 ± 5.2
Colatrella A, 2009 Italy Not clear	76	T2DM (>1 year) Italian Caucasian outpatients in singleton pregnancy	32.7 ± 4.6	Pregestational T2DM (duration >1 year) under specialty care. Autoantibody negativity (GAD65, ICA) noted for classification of normal-weight subgroup at enrolment;	Carpenter–Coustan criteria.	At conception: metformin 28/76; metformin+SU 17/76; SU 3/76; diet 22/76; insulin 6/76. All oral agents were switched to diet ± insulin at	60	Normoglycemic controls	31.0 ± 5.3

				microvascular disease largely absent (no nephropathy/neuropat hy; 2 with background retinopathy).		enrolment; insulin given in split regimens with intermediate-acting component.			
Falhammar H, 2010 Australia NR	11 from 1999 cohort 26 from 2005/2006 cohort	Diabetes in pregnancy (DIP) women. No T1DM.	33.1 ± 1.6 for 1999 cohort 32.6 ± 1.3 for 2005/2006 cohort	Random BGL (first visit; 18, 24, 28, 34 weeks) → if RBGL > 5.5 mmol/L, then 50 g OGCT → if OGCT > 7.8 mmol/L, then 75 g OGTT. Diabetes in pregnancy (DIP) encompassing T2DM (pre-existing or diagnosed ≤12 weeks), GDM (diagnosed >12 weeks), and (in this cohort) no T1DM identified	NR	Yes, 2/11 (18%) on insulin	247 from 1999 cohort<br><br>170 from 2005/2006 cohort	Non-DIP pregnant women	24.6 ± 0.3 for 1999 cohort 25.8 ± 0.5 for 2005/2006 cohort
Lapolla A, 2008 Italy Not clear	164	T2DM	33.2 ± 4.8	NR	NR	All insulin	504	T1DM	29.9 ± 4.8
Marin J 2010, Romania Not clear	11	T2DM	NR	NR	NR	NR	225 healthy pregnancies<br><br>62 T1DM<br><br>80 GDM	Healthy pregnancies without diabetes/obesity, T1DM GDM	27.66 ± 4.5 healthy pregnancie s NR NR
Min Y, 2005 UK NR	17	T2DM	32.1 ± 6.9	NR	NR	Yes → Insulin	39 nondiabetic<br><br>32 T1DM	Nondiabetic pregnant women T1DM	29.7 ± 5.1 nondiabetic 31.1 ± 4.8 T1DM
Abrão Szylit N, 2007 Brazil Cross-sectional study	48	Pregnant women who underwent ECO because of diabetes, for the identification of presence of fetal cardiac	34.3± NR	NR	NR	T2DM Insulin: 41 (85.4%) Diet: 7 (14.6%)	125 GDM 5 T1DM	Pregnant women who underwent ECO because of diabetes, for the identification of presence of fetal cardiac disorders.	33.7± NR 27.4± NR

		disorders.							
Gonzalez-Gonzalez NL, 2008, Spain Not clear	147	T2DM	34 (30–38)	NR	NR	NR	257	T1DM	29 (25–32)
Olmos PR, 2009, Chile NR	51	T2DM	32.86 ± 0.68	NR	NR	Yes — all on intensified insulin therapy (NPH + Regular); pregestational therapy: diet/oral agents in 50, insulin in 1	23	T1DM	28.33 ± 1.11
Roland JM, 2005 UK Not clear	146	T2DM	33.9 ± 5.2	NR	NR	Diet: 28.8% Insulin: 29.7% (4 also on OHA) Sulphonylureas: 15.2% Metformin: 35.2% Glitazones: 2.8% Acarbose: 1.4% Repaglinide: 1.4%	389	T1DM	29.8 ± 5.5
Westgate JA, 2006, New Zealand Not clear	39	T2DM	34.8 ± 5.2	NR	NR	Insulin therapy: Intensive regimens: multiple daily injections Insulin pumps used when daily dose >200 IU Oral agents: NO Diet alone: NO for T2DM Metformin: Not used in this study population.	95 normoglycemic controls<br>138 GDM	normal glucose tolerance<br>GDM	29.3 ± 6.4<br>33.0 ± 5.9

Cyganek K, 2011<br>Poland<br>NR	63	T2DM	33.1 (95%CI 31.9-34.4)	NR	WHO	Before pregnancy: 36/70 (51.4%) on oral glucose-lowering drugs 34/70 (48.6%) on diet only<br>During pregnancy: 66/70 (94.3%) switched to MDI insulin<br>4/70 remained on diet only<br>Most used human prandial insulin; insulin started at 13.8 ± 7.3 weeks of pregnancy.	275	T1DM	27.8 (95% CI 27.2–28.4)
Handisurya A, 2011<br>Austria<br>Not clear	66	T2DM	35.28 ± 5.20	NR	ADA	Insulin therapy: 7.7%<br>Oral antihyperglycemic drugs before pregnancy: many used, discontinued at pregnancy diagnosis (exact n not split by T2DM subgroup)<br>Diet alone: the remainder	75	T1DM	30.39 ± 5.91
Knight K, 2012<br>USA<br>Retrospective cohort study	213	T2DM	37.7 ± 6.1	NR	ADA	Insulin: 82.2%<br>Oral agents: 10.3%<br>No therapy: 7.5%	213	normal glucose tolerance	28.3 ± 5.6
Murphy H, 2011<br>UK<br>Prospective	274	T2DM	34 (26–40)	NR	NR	Diet only: 26.6%<br>Insulin: 27.7% at conception; 89.6% at	408	T1DM	30 (21–38)

cohort study						delivery<br>Metformin: 55.5%<br>Sulphonylureas: 5.8%			
de Oliveira Baraldi C, 2012<br>Brazil<br>NR	9	Obese T2DM	36 (32–38)	NR	NR	Metformin: 850 mg every 12h<br>Insulin	8	Normoglycemic obese PCOS pregnant women	26 (22–33)
Higgins M, 2013<br>Ireland<br>Prospective cohort study	10	T2DM	37 (32–40)	NR	NR	Yes – all T2DM women converted to subcutaneous insulin at 5–9 weeks.<br>Oral agents discontinued early pregnancy.<br>Diet or metformin not used.	30 without DM<br><br>40 T1DM	Without DM<br><br>T1DM	33 (19–42) without DM<br><br>34 (26–42) T1DM
Min Y, 2014<br>United Kingdom<br>RCT	47	T2DM	37 (27–45)	NR	NR	NR	27	Healthy women	29 (18–44)
Sato T, 2014<br>Japan<br>Not clear	579	T2DM	32.9 ± 5.0	NR	NR	All women received diet therapy ± insulin.	369	T1DM	30.9 ± 6.9
Hall D, 2015,<br>South Africa<br>Not clear	92	T2DM	33 (21–45)	NR	NR	Metformin 93.5%<br>Sulfonylurea 1.1%<br>Insulin 53.3% (prandial added in 10 women)	19	T1DM	27 (17–39)
Huynh J, 2015<br>USA<br>NR	37	T2DM	32.1 ± 5.6	NR	NR	Insulin use: 85.3%	36	T1DM	32.2 ± 6.1
Owens L, 2015<br>Ireland	108	T2DM	33.7 ± 4.8	NR	IADPSG	NR	215	T1DM	31.9 ± 5.6

case-control study									
Park S, 2015<br>South Korea<br>Not clear	71	Overt DM	33.7 ± 4.1	FPG ≥7.0 mmol/L OR HbA1c ≥6.5%	modified IADPSG	Insulin: 65/71 (91.3%)<br>Mean insulin dose: 51.1 ± 34.6 U/day<br>Diet/exercise: all patients received lifestyle counseling.<br>Metformin: Not used (not reported).	463<br><br>1781	normal pregnancy<br><br>GDM	32.5 ± 3.5 normal pregnancy<br><br>33.9±3.8 GDM
Wright L<br>2015<br>USA<br>Retrospective cohort study	37	T2DM	34 ± 5	NR	Carpenter and Coustan Criteria	Insulin	48	T1DM	28 ± 6
Abell S, 2016<br>Australia<br>Retrospective cohort study	124	T2DM	33.6 ± 5.3	NR	Australasian Diabetes in Pregnancy Society 1998	Insulin: 125 pregnancies<br>Insulin + metformin: 21<br>Metformin only: 3<br>Diet therapy not reported.	27075	T1DM	29.4 ± 5.4
Cade WT, 2016<br>USA<br>NR	28	Obese T2DM	31 ± 6	duration ≤10 years, HbA1c ≤8% for ≥3 months before pregnancy	White classification	Yes: insulin 100% NPH BID<br>Rapid-acting (Aspart/Humalog) 73%<br>Short-acting (Regular) 13%	26	obese non diabetic	25 ± 5
Hammoud N, 2016<br>Netherlands<br>NR	44	T2DM	34 ± 5	NR	NR	All mothers: insulin therapy only.	78	T1DM	33 ± 4
Villarroel C, 2016,<br>Chile<br>Not clear	21	T2DM	NR	NR	WHO 1999	Yes, treated as follows at Visit 1:<br>Insulin only: 28.6% (6/21)	24	Healthy pregnant women	NR

						Metformin only: 28.6% (6/21) No medication: 42.8% (9/21)			
Billionnet C, 2017 France Cross-sectional study	1907	Women with pre-existing type 2 diabetes identified by insulin or oral agent use before pregnancy and hospital diagnosis	33.5 ± 5.5	NR	NR	Yes 77 % on insulin	735 519 non-diabetic 57 629 GDM	Women without diabetes or with GDM identified from national databases	mean age 29.5 ± 5.3 (non-DM) 31.9 ± 5.5 (GDM)
Cade WT, 2017 USA Not clear	25	Obese women with pre-gestational T2DM (White class B or C) on insulin therapy; mean maternal age 30 ± 6 years.	30 ± 6	NR	ADA	Yes Insulin only	24 obese, without DM 23 non-obese, non-DM	Obese women without diabetes (BMI 30–45 kg/m²) Lean women without diabetes (BMI ≈ 22 kg/m²)	25 ± 5 years 23 ± 3 years
Cnattingius S, 2017 Sweden Not clear	711	Mothers with pre-gestational type 1 or type 2 DM; singleton pregnancies; infants without malformations	35±NR	NR	NR	NR	1 337 099 no DM 5 941 T1DM	Women without pre-existing diabetes (neither type 1 nor type 2), with singleton live births recorded in the Swedish Medical Birth Register during the same period	mean age 30 31 ± NR
Joshi T, 2017 Australia Not clear	88	T2DM pregnant women	33 ± 6	NR	NR	insulin 88 %, metformin 44 %	159	T1DM pregnant women	28.2 ±6
Ladfors L, 2017 Sweden Not clear	87	Pregnant women with pre-existing type 2 diabetes mellitus	34.7 ± 4.9	NR	NR	Yes ins, met	221	T1DM pregnant women	31.9 ± 5.0

Saikia DM, 2017 Assam Not clear	13	T2DM pregnant women	29.80 ± 4.12	NR	ADA	Yes, type NR	29	GDM pregnant women	28.43 ± 3.13
Villarroel C, 2017 Chile Not clear	21	T2DM pregnant women	33.0 (25.9–41.8)	NR	ADA	Yes, insulin and metformin	24 GDM 24 nonDM	GDM and non DM pregnant women	32.6 (20–43) 28.1 (17.3–42.1)
Alessi J, 2018 Brazil Not clear	135	NR	33.5 ± 5.5	NR	NR	7.5% oral, 38.8% insulin only, 53.7% combination oral + insulin.	85	T1DM pregnant womenNR	27 ± 5.7
Endo S, 2018 Japan Not clear	11	NR	38.0 ± 2.7	NR	Japan Diabetes Society guidelines	yes insulin + diet	15	T1DM pregnant women	34.0 ± 5.8
Jang HJ, 2018 South Korea Case–control study	100	T2DM pregnant women with mean duration of diabetes 3.7 ± 4.2 years	33.8 ± 4.5	NR	NR	yes insulin and/or oral agents	100 non diabetic	Healthy pregnant women without diabetes or other chronic disease; matched to T2DM patients by age, BMI, parity and gestational age at delivery.	33.7 ± 3.7
Maple-Brown LJ, 2018 Australia Prospective cohort study	230	Women with pre-existing T2DM, predominantly Indigenous;	32.3 ± 5.9 (non-Indigenous) / 31.1 ± 5.7 (Indigenous)	HbA1c ≥ 6.5 % (48 mmol/mol)	NR	Yes – Insulin, Metformin	60 DIP 830 GDM	Newly diagnosed T2DM in pregnancy (DIP); GDM diagnosed by OGTT (75 g);	31.4 ± 5.3 (non-Indigenous) / 29.8 ± 5.0 (Indigenous) 30.6 ± 5.2 (non-Indigenous) / 28.7 ± 5.0 (Indigenous)

Scherneck S, 2018 Germany Prospective cohort study	87	Pregnant women with pre-existing type 2 diabetes mellitus, treated with metformin (monotherapy or in combination with insulin).	32 (29–35)	NR	ADA	Yes median dose 1500 mg/day, IQR 1000–2000	1011	Pregnant women without diabetes or metformin exposure, recruited from the same Embryotox Pharmacovigilance database.	32 (28–35)
Shimizu I, 2018 Japan Not clear	89	Pregnant women with pregestational type 2 diabetes mellitus, all treated with insulin during pregnancy.	NR	FPG ≥ 7.0 mmol/L ili HbA1c ≥ 6.5%	JDS criteria	Yes – Insulin therapy (all participants)	47	Pregnant women with pregestational type 1 diabetes mellitus, all treated with insulin, included in the multicenter Japan Glycated Albumin Study Group.	NR
Agha-Jaffar R, 2019 United Kingdom Case–control study	80	80 pregnant women with previously diagnosed T2DM ≥ 3 months before conception	34.2 ± 5.1	FPG ≥ 7 mmol/L, HbA1c ≥ 6.5 %,	NICE guidelines	yes Diet, metformin and/or insulin (insulin 77 %)	80	Women with GDM diagnosed at 24–28 weeks; no previous DM;	33.7 ± 5.5
Bashir M, 2019 Qatar Retrospective cohort study	417	T2DM pregnant women managed at tertiary diabetes pregnancy clinic; mean duration of diabetes 8.6 ± 5.3 years	34.7 ± 6.9<br>32.2 ± 6.2	FPG ≥ 7.0 mmol/L, HbA1c ≥ 6.5 %, or 2-h plasma glucose ≥ 11.1 mmol/L on OGTT	ADA	Yes – 83 % insulin ± metformin; 17 % diet only	1419 nonDM<br><br>652 GDM	Normoglycemic women with normal 75 g OGTT and no history of diabetes<br><br>GDM pregnant women managed at a tertiary diabetes pregnancy clinic	29.6 ± 5.5
Ásbjörnsdóttir B, 2019 Denmark Retrospective cohort study	96	T2DM pregnant women followed at the Center for Pregnant Women	34 ± 5	FPG ≥ 7.0 mmol/L, HbA1c ≥ 6.5 %, or 2-h OGTT ≥ 11.1 mmol/L	ADA	yes Insulin in 19 % already at first antenatal visit; all transitioned	108	T1DM	31 ± 5

						to insulin therapy early in pregnancy. Before conception, 63 % used oral agents (metformin ± GLP-1 agonist), 24 % diet only.			
Egan A, 2019 Republic of Ireland Not clear	56	Women with pre-existing type 2 diabetes, enrolled in the same audit and time period, singleton pregnancies.	34.2 ± 5.8	fasting glucose ≥7.0 mmol/L or HbA1c ≥6.5%	ADA	Yes – diet 10.7%, metformin 42.9%, insulin 25%, insulin+metform in 21.4%.	122	Women with pre-existing type 1 diabetes, enrolled in the Irish national perinatal audit 2015–2017, singleton pregnancies, attending tertiary maternity centers.	30.8 ± 5.0
Kong L, 2019 Finland Not clear	3740	Pregnant women with non–insulin-treated type 2 diabetes, Pregnant women with pre-existing insulin-treated diabetes (mostly type 1) identified from Finnish Medical Birth Register and linked health registries, 2004–2014.	NR	NR	NR	Yes – oral antidiabetic drugs (mostly metformin); not insulin.	542735 nonDM<br><br>4000 T1DM	All remaining women without any form of diabetes during pregnancy.	NR
Da Rocha Oppermann ML, 2019 Brazil Retrospective cohort study	128	Pregnant women with type 2 diabetes mellitus prior to conception, attending the Diabetes and Pregnancy	32.1 ± 6.5	NR	NR	Yes – insulin therapy, some on combined regimens (insulin ± metformin).	78	Pregnant women with type 1 diabetes mellitus prior to conception, same institution.	28.5 ± 6.7

		Outpatient Clinic, Porto Alegre, Brazil.							
Mackin S, 2019 United Kingdom retrospective cohort study	373	Pregnant women with type 2diabetes included in the UK National Pregnancy in Diabetes (NPID) audit.	35.4 ± 5.5	NR	UK guidelines	Yes – insulin therapy (most); small subset on insulin + metformin).	3474	Pregnant women with type 1 diabetes (insulin-dependent) included in the UK National Pregnancy in Diabetes (NPID) audit.	30.4 ± 5.8
Stogianni A, 2019 Greece Not clear	130	Pregnant women with pre-existing type 2 diabetes.	35.9 ± 5.3	NR	NR	Yes – insulin (majority), insulin + metformin (minority).	193	Pregnant women with pre-existing type 1 diabetes (insulin-dependent).	31.8 ± 5.1
Wernimont ,S 2019 USA Prospective cohort study	39	T2DM who were enrolled in a perinatal diabetes program at the University of Iowa between December 2015 and June of 2018. All women were treated by the same care team.	33 ± 0.9	HgbA1c >6.5% in first trimester or established preexisting diagnosis	ADA/ACO G	Yes – 85% insulin, 10% glyburide, 3% metformin.	28 GDM A1<br>35 GDM A2	Women with gestational diabetes controlled with diet only. Women with gestational diabetes requiring insulin or oral agents.	31 ± 1.0<br>31 ± 6.0
Ali D, 2020 Ireland Not clear	50	Pregestational type 2 diabetes	35.5 ± 3.8	NR	NR	44% metformin + MDI, 28% insulin alone, 20% metformin only	124	Pregestational type 1 diabetes	33.8 ± 4.7

López-de-Andrés A, 2020 Spain Retrospective cohort study	4391	Women with pre-existing type 2 diabetes (ICD-9-CM 250.0×, 250.2×); mean age 34.2 ± 5.4 years; 10.5 % pre-existing hypertension, 10.8 % obesity, 1.4 % chronic renal disease, 5.7 % smokers; 18.4 % previous cesarean delivery.	34.2 ± 5.4	NR	NR	NR	2340547 nonDM<br><br>5561 T1DM<br><br>130980 GDM	Pregnant women without any diabetes diagnosis. Women with pre-existing type 1 diabetes identified by ICD-9-CM codes 250.1× and 250.3× in the Spanish National Hospital Discharge Database (SNHDD). Women diagnosed with gestational diabetes (ICD-9-CM 648.8×) according to the two-step screening test (50 g GCT → 100 g OGTT, ≥ 2 abnormal values ≥ 105/190/165/145 mg/dL).	31.1 ± 5.6<br>32.0 ± 5.2<br>33.7 ± 5.0
Ásbjörnsdóttir B, 2020 Denmark Prospective cohort study	90	Pregnant women with pre-existing type 2 diabetes, diagnosed prior to conception according to WHO criteria (HbA1c ≥48 mmol/mol / 6.5%). Singleton pregnancies only.	34 ± 5	HbA1c ≥48 mmol/mol / 6.5%	WHO	96% insulin ± metformin	88	Healthy pregnant women without diabetes, matched for singleton pregnancies and gestational age.	32 ± 4

Hauffe F, 2020 German Retrospective cohort study	118	T2DM	34 (30–38)	NR	NR	Insulin or metformin	218	T1DM	33 (28, 36)
Kattini R, 2020 Canada Not clear	76	T2DM	31.4 ± 5.5	NR	NR	Insulin: 63%	1833 no DM<br>164 GDM	No DM<br>GDM	27.5±6<br>24±5.65
Longmore D, 2020 Australia Not clear	Indigenous: 123 T2DM Non-Indigenous: 21 T2DM	Indigenous and non-Indigenous participants of the Pregnancy and Neonatal Diabetes Outcomes in Remote Australia (PANDORA) cohort	Indigenous 31.3 ± 5.6 Non-Indigenous 34.0 ± 5.6	Women were classified as having GDM based on OGTT results consistent with criteria used during recruitment. Pre-existing type 2 diabetes was determined if diagnosed prior to pregnancy and confirmed in medical records. Women with GDM were diagnosed at a median of 28 weeks' gestation by OGTT. Women who had OGTT or HbA1c results consistent with type 2 diabetes criteria outside of pregnancy were included in the GDM group for the primary analysis which is consistent with guidelines regarding diagnosis of GDM in pregnancy. For these women, diagnosis by OGTT occurred at a median of 27 weeks' gestation and diagnosis by HbA1c occurred at a	Australasian Diabetes in Pregnancy Society guidelines 1999, WHO, and IADPSG for GDM<br><br>NR for T2DM	Yes Insulin in Indigenous group 64% and non-Indigenous group 15%	Indigenous: 261/ Non-Indigenous: 423 GDM Indigenous: 111 / Non-Indigenous: 111 nonDM	Indigenous and non-Indigenous participants with GDM and without hyperglycemia in pregnancy of the Pregnancy and Neonatal Diabetes Outcomes in Remote Australia (PANDORA) cohort	Indigenous 29.2 ± 6.1 / Non-indigenous 31.5 ± 5.2 Indigenous 30.5 ± 5.1 / Non-indigenous 25.0 ± 4.7

				median of 18 weeks' gestation					
Morikawa M, 2020 Japan Retrospective cohort study	109 pregnant women with T2DM, including “overt diabetes in pregnancy” T2DM + HDP (n = 15) T2DM without HDP and CH („T2DM alone“) (n = 75) T2DM + CH (chronic hypertension) (n = 19)	Pregnant women ≥20 years with pre-existing or overt T2DM according to Japanese criteria; singleton pregnancies ≥22 gestational weeks; no fetal chromosomal abnormalities; some with hypertensive disorders of pregnancy (HDP) and/or chronic hypertension (CH).	33.5 ± 0.5	Fasting plasma glucose ≥126 mg/dL, and/or – 2-h plasma glucose ≥200 mg/dL during 75-g OGTT, and/or – Random plasma glucose ≥200 mg/dL plus HbA1c ≥6.5%. Overt DM in pregnancy diagnosed with HbA1c ≥6.5% at 8–12 GW and before delivery	NR	Yes – insulin only during pregnancy	45 T1DM – T1DM + HDP (n = 10) – T1DM without HDP (n = 35)	Pregnant women with pre-existing Type 1 diabetes, singleton pregnancies ≥22 gestational weeks, ≥20 years old, no fetal chromosomal abnormalities, all managed with insulin during pregnancy. Two subgroups exist	32.6 ± 0.8
Starikov R, 2020 USA Retrospective cohort study	129 T2DM 39 SGA placentas, 90 AGA placentas	T2DM pregnant women who received standardized care in a dedicated Diabetes in Pregnancy Program, with complete clinical data and placental histopathology available for analysis.	(T2DM SGA): 33 (IQR 37–27) (T2DM AGA): 33 (IQR 37–29)	NR	NR	Yes insulin	85 T1DM 22 SGA, 63 AGA	T1DM pregnant women managed within the same Diabetes in Pregnancy Program	(T1DM SGA): 29 (IQR 33–26) (T1DM AGA): 28 (IQR 32–25)
Gualdani E, 2021 Italy NR	6060	T2DM singleton preganat women aged 15–45 year, in Tuscany, Italy	33.8 ± 4.9	NR	NR	Antidiabetic therapy: Metformin: 54.6%	373 T1DM 184028 noDM	T1DM and no DM singleton pregnant women aged 15–45 year,	32.2 ± 4.8 31.4 ± 5.2

						Oral non-metformin: 6.4%<br>Insulin + oral drugs: 39.0%		in Tuscany, Italy	
Guarnotta V, 2021<br>Italy<br>Retrospective cohort study	62	T2DM	35.2 ± 5.5	NR	NR	Therapy: Yes – insulin (pregnancy); pre-pregnancy oral agents	73	T1DM	29.5 ± 4.5
Martínez-Cruz C, 2021<br>Mexico<br>Not clear	70	T2DM	32.1 ± 7.6	NR	ADA	Yes – insulin	95 T1DM<br>101 GDM<br>57 nonDM	T1DM<br>GDM<br>non DM	28.6 ± 6.2<br>T1DM<br>26.9 ± 6.3<br>GDM<br>26.4 ± 5.6<br>No DM
McLean A, 2021<br>Australia<br>Not clear	43	All pregnant women with T2DM diabetes who birthed in a regional hospital over 12 months were included	NR	NR	IADPSG	Insulin: Yes – 90%<br><br>Metformin: Yes - 10%<br><br>Diet only: No	232	All pregnant women with GDM diabetes who birthed in a regional hospital over 12 months were included	NR
Saquib S, 2021<br>United Arab Emirates<br>Not clear	121	T2DM pregnant women delivering in a tertiary hospital	<25 years: 3%<br>25–35 years: 40%<br>>35 years: 56%<br>36 years (mean)	fasting ≥126 mg/dL<br>2-h postprandial ≥200 mg/dL	IADPSG	Yes – insulin	28	T1DM pregnant women delivering in a tertiary hospital	<25 years: 10%<br>25–35 years: 57%<br>>35 years: 32%<br>31.5 years (mean)
Seah JM, 2021<br>Australia<br>Not clear	106	T2DM pregnant women who delivered in a tertiary obstetric medical centre, over a 10-year period identified via hospital	34.4 ± 5.1	NR	NR	Insulin: Yes (majority, ali % NR)	119 noDM<br>92 T1DM	Healthy and T1DM pregnant women	29.1 ± 4.8<br>31.1 ± 5.7

Britten F, 2022, Australia Prospective cohort study	14	T2DM.	35.1 ± 5.2	NR	NR	Yes metformin i/ili insulin, % NR	10 BMI-Matched 12 Normal BMI range non DM	Two control groups with age- and parity-matched nondiabetic pregnant women: BMI matched (n = 18) or normal-range BMI (n = 18)	33.1 ± 5.6 34.0 ± 4.1
Rao C, 2022 China Prospective cohort study	43	T2DM pregnant women with HIP requiring insulin therapy	36.02 ± 4.95	NR	Chinese Diabetes Society guidelines	Yes insulin	24 T1DM+MODY 54 GDM	T1DM, MODY, and GDM pregnant women with HIP requiring insulin therapy	32.48 ± 3.25 35.41 ± 3.49
Jacobsen D, 2022 Norway NR	16	T2DM singleton pregnant women without history of hypertension or other inflammatory diseases (e.g., autoimmunity or cancer) prior to deliveryntrols).	34.8 (29.2–37.6)	NR	WHO	Yes T2DM- Insulin ± metformin	269 nonDM n = 64 T1DM n = 35 GDM	Non DM, T1DM, and GDM singleton pregnant women without history of hypertension or other inflammatory diseases (e.g., autoimmunity or cancer) prior to deliveryntrols).	33.8 (30.7–36.4) 32.4 (28.5–36.1) 35.2 (31.9–39.5)
Kapustin R, 2022 Russia Retrospective cohort study	229	T2DM pregnant women over 10 years on diet or insulin	T2DM insulin: 34.0 ± 5.2 T2DM diet: 33.6 ± 5.3	NR	NR	Yes Diet: n=95 Insulin: n=134	506 T1DM 2387 GDM 139 Non DM	T1DM, GDM, and non DM pregnant women over 10 years	T1DM CSII 28,7 ± 4,6 T1DM MDII 28,4 ± 4,8 GDM Diet 31,2 ± 5,0 GDM Insulin 31,3 ± 5,1 Non DM 30,8 ± 4,8
Kapustin R 2022a, Russia,	35	T2DM preceding singleton	33.4 (31.1–35.6) – diet 33.9 (31.6–	NR	NR	Yes Diet: n=15 Insulin: n=20	20 T1DM in preconception planning	T1DM (two groups: T1DM patients who	29.7 (27.6–31.8) T1DM not

Prospective cohort study		pregnant women aged 18 years or more	36.1) – insulin				group<br><br>20 T1DM in preconception non-planning group<br><br>15 healthy controls	were not involved in preconception planning, HbA1c level > 6.5% and T1DM patients who were involved in preconception planning no later than six months before conception, HbA1c level < 6.5%, and healthy preceding singleton pregnant women aged 18 years or more	planned 28.7 (26.5–30.8) T1DM planned 28.9 (26.3–31.5) healthy control
Cordero L, 2022 USA Retrospective cohort study	212	T2DM pregnant women	33.5 ± 5.3	NR	NR	insulin ± metformin (no % given)	185	T1DM	29.8 ± 5.5
Cordero L, 2022 USA Retrospective cohort study	294	T2DM pregnant women	33 (29–37)	NR	NR	Yes insulin metformin	171	T1DM	30 (25–34)
Malaza N, 2023 South Africa Not clear	65	T2DM pregnant women	35 (30–37)	T2DM fasting plasma glucose level ≥7.0 mmol/L, random plasma glucose or 2-hour plasma glucose ≥11.1 mmol/L on the OGTT; or glycated haemoglobin (HbA1c) ≥6.5%).<br><br>TIDM if diagnosed prior to pregnancy or if first	IADPSG	Yes at enrollment (2nd trimester) T2DM: metformin 53.3% T1DM: insulin 76.9% GDM: diet 28.7% at delivery T2DM: metformin+insul	66 noDM 13 T1DM 39 GDM	T1DM, GDM, and non DM pregnant women	31 (27–36) 29 (27–32) 35 (32–38)

				diagnosed in pregnancy and was confirmed by the presence of positive antibodies or the occurrence of diabetic ketoacidosis.<br><br>GDM -fasting plasma glucose level 5.1 - 6.9 mmol/L or 1-hour plasma glucose ≥10 mmol/L or 2-hour plasma glucose 8.5 - 11.0 mmol/L after a 2-hour 75-g oral glucose tolerance test		in 67.2%<br>T1DM: insulin 81.8%<br>GDM: metformin 39.4%			
Koyama M, 2023<br>Japan<br>Not clear	15	T2DM pregnant women	36 (28–41)	NR	IADPSG	Yes, diet	22	T1DM pregnant women	NR
Powers Carson J, 2024<br>USA<br>Case-control study	14	T2DM pregnant women with BMI ≥25 kg/m2	30.5 (19–39)	Hb A1c ≥6.5%, fasting plasma glucose ≥126 mg/dL (7.0 mmol/L), 1 h glucose challenge test ≥185 mg/dL (10.3 mmol/L), or oral glucose tolerance test 2-step method (1 and 3 h samples)	IADPSG	NR	21 GDM<br>28 nonDM	GDM and non DM pregnant women with BMI ≥25 kg/m2	30.0 (22–42)<br>28.0 (17–38)
Kapustin R, 2024<br>Russia<br>Retrospective cohort study	214	T2DM pregnant women	32.1 ± 5.4	NR	WHO	Yes, insulin and metformin	498 T1DM<br>2357 GDM	T1DM and GDM pregnant women	29.6 ± 5.5<br>30.7 ± 5.5
Ballesteros M, 2024<br>Spain<br>Prospective cohort study	124	Pregnant women with T2DM	34.94 ± 5.2	NR	NR	NR	141	T1DM pregnant women	31.8 ± 4.6

Grazia Dalfrà M, 2024 Italy Not clear	68	T2DM pregnant women	34.7 ± 5.3	NR	IADPSG	NR	18	Pregnant women with overt DM	34.2 ± 6.6
Suzuki T, 2024 Japan Not clear	69	T2DM Japanese pregnant women	34.8 ± 4.5	NR	NR	Yes insulin all	135	T1DM Japanese pregnant women	32.7 ± 4.0
Dias S, 2025 South Africa Not clear	53 Preexisting T2DM 24 Newly diagnosed T2DM in pregnancy	Preexisting and newly diagnosed T2DM pregnant women aged between 18 and 40 years, self-reported black African, less than 28 weeks gestation, and human immunodeficiency virus (HIV) negative	36.0 (30.0−37.0) 33.5 (30.0−36.0)	NR	IADPSG 2010 i WHO 2013	NR	69 Normoglycemia (controls) 26 T1DM 58 GDM	T1DM, GDM, and normoglycemic pregnant women aged between 18 and 40 years, self-reported black African, less than 28 weeks gestation, and human immunodeficiency virus (HIV) negative	31.0 (27.0–36.6) Controls 29.0 (27.0–32.0) T1DM 35.5 (32.0–38.0) GDM
Gherbon A, 2025 Romania Not clear	107	T2DM singleton pregnancies from spontaneous conception admitted to the hospital during the study period with sufficient recorded data, who accept to participate in the study, and the absence a mental illness	33.18 ± 6.24	Fasting: 92 mg/dL (5.1 mmol/L), 1h: 180 mg/dL (10.0 mmol/L), or 2h: 153 mg/dL (8.5 mmol/L)	NR	93 (86.91%) insulin 14 (13.09%) diet	958 controls 279 GDM 74 T1DM	T1DM, GDM, and normoglycemic controls in singleton pregnancies from spontaneous conception admitted to the hospital during the study period with sufficient recorded data, who accept to participate in the study, and the absence a mental illness.	29.64 ± 5.91 31.78 ± 5.60 31.08 ± 6.14

Manga J, 2025<br>South Africa<br>Not clear	87	T2DM pregnant women	35.3 ± 4.3	HbA1c ≥ 6.5% or random glucose ≥ 11.1 mmol/L confirmed by repeat testing.	IADPSG	Yes<br>20–25% insulin, rest diet	27 T1DM<br>118 GDM<br>66 overt DM	T1DM, GDM, and overt DM pregnant women	27.8 ± 6.2<br>35.1 ± 5.3<br>34.3 ± 5.3
Zhou X, 2025<br>China<br>Not clear	221	Pregnant women aged ≥ 18 years with continuous glucose monitoring	NR	NR	IADPSG	NR	T1DM: 26<br>GDMA1: 140<br>GDMA2: 182	Pregnant women aged ≥ 18 years with continuous glucose monitoring	NR
