# Supplementary material for: Body Weight-Related Parameters in Pregnancies Complicated by Type 2 Diabetes Mellitus: A Systematic Review and Meta-Analysis with Maternal and Perinatal Outcome Mapping
Source: J Clin Med. 2026 Jul 6;15(13):5260. doi: 10.3390/jcm15135260 (PMC13362816; doi:10.3390/jcm15135260)
Supplement: Supplementary file 1 [file jcm-15-05260-s001.zip › Supplementary Table S3. Reasons for exclusion reported in the included studies.pdf]

Table S3. Reasons for exclusion

Author, year Country	Nulliparity	PCOS	HTA	CVD	Smoking	Renal diseases	Liver disease	T1DM Family history	T2DM Family history	GDM Family history	Other comorbid ity	Other
Barnard R, 1997 Australia,	NR	NR	No	NR	NR	NR	NR	NR	NR	NR	NR	Multifetal pregnancies; women failing consent; gestation
Ben Slama C, 1997 Tunis	NR	NR	No	NR	NR	No	NR	NR	NR	NR	NR	NR
Colatrella A, 2009 Italy	No	NR	No	NR	No	NR	NR	NR	NR	NR	NR	Multifetal pregnancy;
Contreras-Soto J, 1991 Mexico	NR	NR	NR	NR	NR	NR	NR	NR	NR	NR	NR	NR
Cundy T, 2000 New Zealand	No	NR	No	NR	NR	No	NR	NR	NR	NR	NR	Multiple pregnancies
Cundy T, 2002 New Zealand	No	NR	No	NR	No	No	NR	NR	NR	NR	NR	Twin pregnancies and uncertain diabetes type excluded.
Cundy T, 2007 New Zealand	No	NR	NR	NR	No	NR	NR	NR	NR	NR	NR	Twins Spontaneous miscarriages before 20 weeks and terminations for nonmedical

												reasons were not included
Falhammar H, 2010 Australia	NR	NR	NR	NR	NR	NR	NR	NR	NR	NR	NR	NR
Lapolla A, 2008 Italy	NR	NR	NR	NR	NR	NR	NR	NR	NR	NR	NR	NR
Marin J 2010, Romania	No	NR	No	NR	Yes	NR	NR	NR	NR	NR	NR	Inflammatory diseases, lupus, immunologic diseases, benign/malignant tumors, alcohol/drug abuse, smoking, rheumatoid arthritis
Min Y, 2005 UK	NR	NR	Yes	Yes	NR	Yes	Yes	NR	NR	NR	NR	Twin pregnancy, preexisting high blood pressure, prior PE, chronic diseases, illegal drug use, underage < 16 y, cesarean section
Abrão Szylit N, 2007 Brazil	NR	NR	NR	NR	NR	NR	NR	NR	NR	NR	NR	multiple pregnancy criteria, inconclusive ECO, missing chart and/or delivery did not occur at the hospital and there was no

												confirmation of the NB cardiologic follow-up.
Gonzalez-Gonzalez NL., 2008, Spain	NR	NR	NR	NR	NR	NR	NR	NR	NR	NR	NR	multiple pregnancy, GDM
Olmos PR, 2009, Chile	NR	NR	NR	NR	NR	NR	NR	NR	NR	NR	NR	NR
Roland JM , 2005 UK	NR	NR	NR	NR	NR	NR	NR	NR	NR	NR	NR	NR
Westgate JA, 2006, New Zealand	NR	NR	No	NR	No	NR	NR	NR	NR	NR	NR	Miscarriage, moved out of area, twin pregnancy, delivery <32 weeks, intrauterine fetal death, termination for fetal abnormality, cord blood not collected or hemolyzed, delay in sample collection >20 minutes or freezing delay >60 minutes, antenatal glucocorticoids within 24h before birth
Yang X, 2002 China	NR	NR	NR	NR	NR	NR	NR	NR	NR	NR	NR	Age < 18, multiple gestations, ABO incompatibility

												(titer >1:30), pre-pregnancy diabetes, long-term medical treatment affecting glucose metabolism
Cyganek K, 2011 Poland	NR	NR	NR	NR	NR	NR	NR	NR	NR	NR	NR	NR
Handisurya A, 2011 Austria	NR	NR	NR	NR	NR	NR	NR	NR	NR	NR	NR	Multiple pregnancies, terminations for social indications, secondary diabetes, cortisone-induce)
Knight K, 2012 USA	No	NR	No	NR	No	NR	NR	NR	NR	NR	thyroid disease	NR
Murphy H, 2011 UK	NR	NR	NR	NR	No	NR	NR	NR	NR	NR	NR	NR
de Oliveira Baraldi C, 2012 Brazil	NR	NR	NR	NR	NR	NR	NR	NR	NR	NR	NR	NR
Higgins M, 2013 Ireland	No	NR	NR	NR	NR	NR	NR	NR	NR	NR	NR	NR
Min Y, 2014 United Kingdom	No	NR	NR	NR	NR	NR	NR	NR	NR	NR	NR	NR
Sato T, 2014 Japan	NR	NR	NR	NR	NR	NR	NR	NR	NR	NR	NR	NR

Hall D, 2015, South Africa	NR	NR	No	NR	NR	No	NR	NR	NR	NR	No	NR
Huynh J, 2015 USA	NR	NR	NR	NR	NR	NR	NR	NR	NR	NR	NR	Missing placental data, missing race, multiple gestations, repeated pregnancies.
Owens L, 2015 Ireland	NR	NR	NR	NR	NR	NR	NR	NR	NR	NR	NR	NR
Park S, 2015 Korea	NR	NR	NR	NR	NR	NR	NR	NR	NR	NR	NR	NR
Wright L 2015 USA	NR	NR	NR	NR	NR	NR	NR	NR	NR	NR	NR	NR
Abell S, 2016 Australia	NR	NR	NR	NR	NR	NR	NR	NR	NR	NR	NR	T1DM, GDM, multiple pregnancy
Cade WT , 2016 USA	NR	NR	NR	NR	NR	NR	NR	NR	NR	NR	NR	NR
Hammoud N, 2016 Netherlands	NR	NR	NR	NR	NR	NR	NR	NR	NR	NR	NR	NR
Villarroel C, 2016, Chile	NR	Yes	NR	NR	NR	NR	NR	NR	NR	NR	NR	NR
Billionnet C , 2017 France	No	NR	NR	NR	NR	NR	NR	NR	NR	NR	NR	NR

Cade WT, 2017 USA	NR	NR	NR	NR	No	NR	NR	NR	NR	NR	No	Multiple gestation, drugs, medication use
Cnattingius S, 2017 Sweden	No	NR	No	NR	No	NR	NR	NR	NR	NR	NR	Major congenital malformations, GDM, unclear DM diagnosis
Joshi T, 2017 Australia	No	NR	NR	NR	No	No (nephropathy)	NR	NR	NR	NR	NR	NR
Ladfors L, 2017 Sweden	No	NR	NR	NR	No	NR	NR	NR	NR	NR	NR	Multiple gestations, miscarriages, IUFD, major fetal anomalies or syndromes
Saikia DM , 2017 Assam	No	NR	Yes	NR	NR	NR	NR	NR	NR	NR	Yes	lipid metabolism disorders; BMI > 35; BMI < 18; endocrine disorders; eclampsia; preeclampsia
Villarroel C, 2017 Chile	NR	Yes	NR	NR	NR	NR	NR	NR	NR	NR	Obesity	fetal malformations, preterm <34w, PCOS, endocrine disorders, steroids, ovulation induction
Alessi J, 2018 Brazil	No	NR	No	NR	No	No	NR	NR	No	NR	thyroid disease	Multiple pregnancies

Endo S, 2018 Japan	No	NR	No	NR	NR	No	NR	NR	NR	NR	NR	multiple pregnancy, steroid-induced diabetes
Jang HJ , 2018 South Korea	No	NR	NR	NR	NR	NR	NR	NR	No	NR	NR	GDM, multiple pregnancy, congenital anomalies, and chronic systemic diseases
Maple-Brown LJ , 2018 Australia	No	NR	No	NR	No	NR	NR	NR	NR	NR	obesity	T1DM and < 20 wk gestation
Scherneck S, 2018 Germany	No	No	NR	NR	No	NR	NR	NR	NR	NR	obesity	No
Shimizu I, 2018 Japan	NR	NR	NR	NR	NR	NR	NR	NR	NR	NR	NR	Women with GDM, multiple pregnancies, or incomplete glycemic data, miscarriages were excluded.
Agha-Jaffar R , 2019 United Kingdom	No	NR	No	NR	NR	NR	NR	NR	NR	No	NR	NR
Bashir M, 2019 Qatar	NR	NR	No	NR	NR	NR	NR	NR	NR	NR	Hypothyr oidism	multiple pregnancies, congenital anomalies, and deliveries < 24 weeks
Ásbjörnsdóttir B, 2019 Denmark	No	NR	NR	NR	No	NR	NR	NR	NR	NR	Obesity	Women with gestational diabetes, multiple gestations, or missing dietary data were

												excluded.
Egan A, 2019 Republic of Ireland	No	NR	No	NR	No	NR	NR	NR	NR	NR	retinopathy	Women with MODY (n=6) and post-transplant DM (n=1) excluded; multiple gestations and incomplete data excluded.
Kong L, 2019 Finland	NR	NR	NR	NR	No	NR	NR	NR	NR	NR	NR	Multiple pregnancies, congenital anomalies, and missing registry data excluded; only singleton live births analyzed.
Da Rocha Oppermann ML, 2019 Brasil	No	NR	No	NR	NR	NR	NR	NR	NR	NR	obesity,	Multiple gestation, congenital anomalies, missing data, gestational diabetes (excluded).
Mackin S, 2019 United Kingdom	No	NR	NR	NR	No	NR	NR	NR	NR	NR	NR	Multiple pregnancies, missing clinical data, congenital anomalies, or <24 gestational weeks.
Stogianni A, 2019 Greece	No	NR	No	NR	No	NR	NR	NR	NR	No	obesity, metabolic syndrom	Multiple pregnancies, missing data, gestational

												diabetes, fetal anomalies.
Wang X, 2019 USA	No	NR	No	NR	No	NR	NR	NR	NR	NR	obesity, PE, DKA, bicornuate uterus, consanguinity reported as contributory factors	multiple pregnancy, missing maternal data, gestation <28 or >44 weeks.
Wernimont ,S 2019 USA	No	NR	NR	NR	NR	NR	NR	NR	NR	NR	NR	Type 1 DM, delivery outside study site, missing glucose data
Ali D, 2020 Ireland	No	NR	No	NR	NR	NR	NR	NR	NR	NR	Retinopathy; Hypothyroidism	NR
López-de-Andrés A, 2020 Spain	NR	NR	No	NR	No	No	NR	NR	NR	NR	obesity	NR
Ásbjörnsdóttir B, 2020 Denmark	No	NR	No	NR	No	NR	NR	NR	NR	NR	thyroid disease, dyslipidemia	Multiple gestation, type 1 diabetes, fetal malformations, psychiatric illness or use of psychiatric medication, non-consent
Hauffe F, 2020 German	No	NR	No (chronic hypertension)	NR	No	No (nephropathy)	NR	NR	NR	NR	retinopathy	NR

Kattini R, 2020 Canada	NR	NR	NR	NR	NR	NR	NR	NR	NR	NR	NR	NR
Longmore D, 2020 Australia	No	NR	NR	NR	No	NR	NR	NR	NR	NR	NR	NR
Morikawa M, 2020 Japan	NR	NR	No	NR	NR	No (diabetic nephropathy)	NR	NR	NR	NR	NR	multiple pregnancy, fetal chromosomal abnormality, steroid-induced DM
Starikov R, 2020 USA	NR	NR	NR	NR	NR	NR	NR	NR	NR	NR	NR	NR
Gualdani E, 2021 Italy	No	NR	NR	NR	No	NR	NR	NR	NR	NR	NR	NR
Guarnotta V, 2021 Italy	NR	NR	NR	NR	NR	NR	NR	NR	NR	NR	Thyroid dysfuncti on Dyslipid emia Obesity	NR
Martínez-Cruz C, 2021 Mexico	NR	NR	No	NR	NR	NR	NR	NR	NR	NR	NR	NR
McLean A, 2021 Australia	NR	NR	NR	NR	NR	NR	NR	NR	NR	NR	NR	No
Saquib S, 2021 United Arab Emirates	NR	NR	No	NR	NR	No (nephropathy)	NR	NR	NR	NR	Retinopa thy DKA in pregnanc y	GDM, multiples, incomplete data, deliveries outside hospital excluded

Seah JM, 2021 Australia	No	NR	No	NR	Yes	NR	NR	NR	NR	NR	NR	GDM excluded; non-singleton excluded; incomplete data excluded
Britten F, 2022, Australia	NR	No	No	NR	NR	NR	NR	NR	NR	NR	No – asthma, hypothyroidism, depression	NR
Rao C, 2022 China	NR	NR	NR	NR	NR	NR	NR	NR	NR	NR	NR	NR
Jacobsen D, 2022 Norway	No	NR	No	NR	NR	NR	NR	NR	NR	NR	NR	Chronic HTA, autoimmune/inflammatory diseases, multiple pregnancy, preterm sampling < week 20
Kapustin R, 2022 Russia	NR	NR	NR	NR	NR	NR	NR	NR	NR	NR	NR	Symptomatic DM, severe comorbidities, malignancy, multiple pregnancy, refusal
Kapustin R, 2022a Russia	No	NR	No (gestational hypertension)	No (diabetic vasculopathy)	NR	No (renal vasculopathy)	NR	NR	NR	NR	Obesity	Severe preexisting comorbidities, severe acute condition 3 months before or during pregnancy

Cordero L, 2022 USA	No	NR	No	NR	Yes	NR	NR	NR	NR	NR	No	Multiple gestation, major congenital anomalies, delivery < 34 weeks, no intention to breastfeed, neonatal death before discharge, missing outcome data
Cordero L, 2022 USA	No	NR	No	NR	Yes	NR	NR	NR	NR	NR	NR	Identical for both groups (multifetal gestation, anomalies, <34 GA, incomplete data, neonatal death)
Malaza N, 2023 South Africa	NR	NR	No	NR	NR	NR	NR	NR	NR	NR	NR	NR
Koyama M, 2023 Japan	NR	NR	No	NR	NR	NR	NR	NR	NR	NR	NR	Twin pregnancy; pregnancy loss <22 w
Powers Carson J, 2024 USA	NR	NR	NR	NR	NR	NR	NR	NR	NR	NR	NR	
Kapustin R, 2024 Russia	NR	NR	No	NR	NR	No	NR	NR	NR	NR	Retinopathy neuropathy	Multiple pregnancy; Chromosomal abnormalities
Ballesteros M, 2024 Spain	No	NR	No	NR	NR	NR	NR	NR	NR	NR	NR	NR

Grazia Dalfrà M, 2024 Italy	NR	NR	No	NR	NR	NR	NR	NR	NR	NR	Retinopathy, Neuropathy	NR
Suzuki T, 2024 Japan	No	NR	No	NR	No	No	NR	NR	NR	NR	NR	NR
Dias S, 2025 South Africa	NR	NR	No	NR	NR	NR	NR	NR	NR	NR	NR	NR
Gherbon A, 2025 Romania	NR	NR	No	NR	NR	NR	NR	NR	NR	NR	NR	Mental disorder, insufficient data, rejection for participation, IVF
Hillick D, 2025 Ireland	NR	NR	No	No	Yes	NR	NR	NR	NR	NR	NR	Prematurity <34 w, stillbirth, incomplete data
Manga J, 2025 South Africa	NR	NR	No	NR	NR	NR	NR	NR	NR	NR	NR	NR
Zhou X, 2025 China	NR	NR	NR	NR	NR	NR	NR	NR	NR	NR	NR	NR

Abbreviations: PCOS – polycystic ovary syndrome, HTA – hypertension, CVD – cardiovascular diseases, T1DM – type 1 diabetes mellitus, T2DM – type 2 diabetes mellitus, GDM – gestational diabetes mellitus, PE – preeclampsia, IUFD – intrauterine fetal death, BMI – body mass index, MODY - Maturity-Onset Diabetes of the Young, DKA – diabetic ketoacidosis, GA – gestational age, IVF – in vitro fertilisation
