# Supplementary material for: Body Weight-Related Parameters in Pregnancies Complicated by Type 2 Diabetes Mellitus: A Systematic Review and Meta-Analysis with Maternal and Perinatal Outcome Mapping
Source: J Clin Med. 2026 Jul 6;15(13):5260. doi: 10.3390/jcm15135260 (PMC13362816; doi:10.3390/jcm15135260)
Supplement: Supplementary file 1 [file jcm-15-05260-s001.zip › Supplementary Table S4. Metabolic parameters in cases and controls.pdf]

Supplement material. Table S4. Metabolic parameters

Author, year Country Study design	CASES										CONTROLS							
	Glycemia value (time of measurement)	HbA1c (time of measurement)	HOMA-IR (time of measurement)	Insulin value µIU/mL or mIU/L (time of measurement)	Triglycerides (TG)	Total cholesterol (TC)	LDL	HDL	VLDL	Tg/HDL ratio	Glycemia value (time of measurement)	HbA1c (time of measurement)	HOMA-IR (time of measurement)	Insulin value µIU/mL or mIU/L (time of measurement)	LDL	HDL	VLDL	Tg/HDL ratio
Barnard R, 1997 Australia, Not clear	NR	NR	NR	NR	NR	NR	NR	NR	NR	NR	NR	NR	NR	NR	NR	NR	NR	NR
Ben Slama C, 1997 Tunis Retrospective study	NR	NR	NR	NR	NR	NR	NR	NR	NR	NR	NR	NR	NR	NR	NR	NR	NR	NR
Colatrella A, 2009 Italy Not clear	NR	Pre-pregnancy 6.3 ± 2.1% at conception 6.2 ± 1.7% 3rd trimester 5.3 ± 1.2%	NR	NR	NR	NR	NR	NR	NR	NR	NR	NR	NR	NR	NR	NR	NR	NR
Contreras-Soto J, 1991 Mexico NR	NR	NR	NR	NR	NR	NR	NR	NR	NR	NR	NR	NR	NR	NR	NR	NR	NR	NR
Cundy T, 2000 New Zealand Not clear	NR	7.6 ± 1.8% 1st trim 6.5 ± 1.1% 3rd trim	NR	NR	NR	NR	NR	NR	NR	NR	NR	7.9 ± 1.7% 1st trim 6.8 ± 1.2% 3rd trim	NR	NR	NR	NR	NR	NR
Cundy T, 2002 New Zealand NR	NR	7.8 ± 1.6 2nd trim	NR	NR	NR	NR	NR	NR	NR	NR	NR	7.8 ± 3.9% 2nd trim	NR	NR	NR	NR	NR	NR
Cundy T, 2007 New Zealand Not clear	NR	7.6 ± 1.7% 1st trim 6.1 ± 0.9% 3rd trim	NR	NR	NR	NR	NR	NR	NR	NR	NR	7.6 ± 1.6% 1st trim 6.1 ± 0.9% 3rd trim	NR	NR	NR	NR	NR	NR
Falhammar H, 2010 Australia Not clear	Fasting 5.3 ± 0.3 OGTT 2nd trim 1 h 10.8 ± 0.8,	NR	NR	NR	NR	NR	NR	NR	NR	NR	Fasting 4.4 ± 0.1, OGTT 2nd trim 1 h 7.5 ± 0.3,	NR	NR	NR	NR	NR	NR	NR

	OGTT 2nd trim 2 h 8.1 ± 0.6 OGTT 2nd trim										OGTT 2nd trim 2 h 5.8 ± 0.2 OGTT 2nd trim							
Lapolla A, 2008 Italy Prospective study	NR	Pre-pregnancy 6.4 ± 1.7; 3rd trimester ≈ 6.3 %	NR	NR	NR	NR	NR	NR	NR	NR	NR	Pre-pregnancy 7.2 ± 1.6; 3rd trimester ≈ 7.0 %	NR	NR	NR	NR	NR	NR
Marin J 2010, Romania Retrospective study	NR	NR	NR	NR	NR	NR	NR	NR	NR	NR	NR	NR	NR	NR	NR	NR	NR	NR
Min Y, 2005 UK NR	1st trimester 7.7 ± 3.8; 3rd trimester 6.3 ± 3.8	1st trimester 7.0 ± 1.7; 3rd trimester 6.6 ± 0.9	NR	NR	NR	NR	NR	NR	NR	NR	NR	NR	NR	NR	NR	NR	NR	NR
Abrão Szylit N, 2007 Brazil Cross-sectional study	NR	NR	NR	NR	NR	NR	NR	NR	NR	NR	NR	NR	NR	NR	NR	NR	NR	NR
Gonzalez-Gonzalez NL., 2008, Spain retrospective study	NR	1st trimester: 6.6 ± 1.5% 3rd trimester: 5.5 ± 0.55%	NR	NR	NR	NR	NR	NR	NR	NR	NR	1st trimester: 7.19 ± 1.22% 3rd trimester: 6.09 ± 0.82%	NR	NR	NR	NR	NR	NR
Olmos PR, 2009, Chile NR	NR	2nd trimester: 5.86 ± 0.25% 3rd trimester: 5.87 ± 0.19% Antepartum: 5.93 ± 0.20%	NR	NR	1.89 mmol/L	5.34 mmol/L	2.93 mmol/L	1.40 mmol/L	NR	≈ 3.10	NR	2nd trimester: 6.26 ± 0.20% 3rd trimester: 6.37 ± 0.47% Antepartum: 6.11 ± 0.41%	NR	NR	3.27 mmol/L	1.80 mmol/L	NR	1.82
Roland JM , 2005 UK	NR	1st trimester: 7.22 ± 1.95%	NR	NR	NR	NR	NR	NR	NR	NR	NR	1st trimester: 7.35 ±	NR	NR	NR	NR	NR	NR

prospective study		2nd trimester: 6.09 ± 1.88%<br><br>3rd trimester: 6.58 ± 1.36%										1.79<br><br>2nd trimester: 6.49 ± 1.49<br><br>3rd trimester: 6.51 ± 1.53						
Westgate JA, 2006, New Zealand Not clear	5.1 ± 1.4 1st trim	Week 30: 6.5 ± 1.3%<br><br>End of pregnancy: 6.4 ± 1.3%	NR	77 pmol/L (42–143) 1st trim	NR	NR	NR	NR	NR	NR	5.2 ± 0.9 1st trim	NR	NR	33 pmol/L (18–62) 1st trim	NR	NR	NR	NR
Yang X, 2002 China Prospective study	NR	NR	NR	NR	NR	NR	NR	NR	NR	NR	NR	NR	NR	NR	NR	NR	NR	NR
Cyganek K, 2011 Poland NR	NR	First trimester (“at admission”): 6.2% (95% CI 5.8–6.7)<br><br>Second trimester: 5.6% (95% CI 5.4–5.9)<br><br>Third trimester: 5.6% (95% CI 5.4–5.8)	NR	NR	NR	NR	NR	NR	NR	NR	NR	First trimester: 7.0% (95% CI 6.8–7.2)<br><br>Second trimester: 5.8% (95% CI 5.7–5.9)<br><br>Third trimester: 5.7% (95% CI 5.6–5.8)	NR	NR	NR	NR	NR	NR
Handisurya A, 2011 Austria Not clear	NR	Admission: 6.90 ± 1.44%<br><br>1st trimester: 6.73 ± 1.06%<br><br>2nd trimester: 6.24 ± 0.91%<br><br>3rd trimester: 6.45 ± 1.27%	NR	NR	NR	NR	NR	NR	NR	NR	NR	Admission: 7.44 ± 1.73%<br><br>1st trimester: 6.72 ± 1.13%<br><br>2nd trimester:	NR	NR	NR	NR	NR	NR

												6.02 ± 0.99%<br><br>3rd trimester: 6.07 ± 0.79%						
Knight K, 2012 USA Retrospective cohort study	NR	6.9% in 1st trimester	NR	NR	NR	NR	NR	NR	NR	NR	NR	NR	NR	NR	NR	NR	NR	NR
Murphy H, 2011 UK Prospective cohort study	NR	Pre-pregnancy median: (6.9%)<br><br>1st trimester: (6.8%)<br><br>2nd trimester: (6.0%)<br><br>3rd trimester: (6.2%)	NR	NR	NR	NR	NR	NR	NR	NR	NR	Pre-pregnancy: (7.9%)<br><br>1st trimester: (7.4%)<br><br>2nd trimester: (6.7%)<br><br>3rd trimester: (6.7%)	NR	NR	NR	NR	NR	NR
de Oliveira Baraldi C, 2012 Brazil not clear	5.33 mmol/L	Median 5.8% (third trimester)	NR	NR	NR	NR	NR	NR	NR	NR	4.66 mmol/L.	NR	NR	NR	NR	NR	NR	NR
Higgins M, 2013 Ireland Prospective cohort study	NR	HbA1c early pregnancy: 6.3% (4.9–7.3)<br><br>HbA1c at 36 weeks: 5.8% (4.4–6.3)		NR	NR	NR	NR	NR	NR	NR	NR	NR	NR	NR	NR	NR	NR	NR
Min Y, 2014 United Kingdom RCT	NR	7.0% at 1st trim	NR	NR	NR	NR	NR	NR	NR	NR	NR	NR	NR	NR	NR	NR	NR	NR
Sato T, 2014 Japan Retrospect	NR	6.9 ± 1.7% 1st trim	NR	NR	NR	NR	NR	NR	NR	NR	NR	6.8 ± 1.3 1st trim	NR	NR	NR	NR	NR	NR

ive study																		
Hall D,<br>2015,<br>South<br>Africa<br>Not clear	NR	HbA1c at booking: 7.5% (5.0–13.5)<br><br>HbA1c pre-delivery: 6.6% (4.4–10.4)	NR	NR	NR	NR	NR	NR	NR	NR	NR	NR	NR	NR	NR	NR	NR	NR
Huynh J,<br>2015<br>USA<br>not clear	NR	1st trimester: 7.4 ± 1.6%<br><br>2nd trimester: 6.3 ± 0.9%<br><br>3rd trimester: 6.4 ± 0.9%	NR	NR	NR	NR	NR	NR	NR	NR	NR	NR	NR	NR	NR	NR	NR	NR
Owens L,<br>2015<br>Ireland<br>case-control<br>study	NR	Overall pregnancy 5.8%<br>Trimester 1 6.9 ± 1.7%<br>Trimester 2 6.1 ± 1.1%<br>Trimester 3 5.9 ± 0.67%<br>Term 5.7 ± 0.45%	NR	NR	NR	NR	NR	NR	NR	NR	NR	Overall 6.6%<br>T1 7.7 ± 1.6%<br>T2 6.7 ± 1.1%<br>T3 6.4 ± 0.95%<br>Term 6.4 ± 0.88%	NR	NR	NR	NR	NR	NR
Park S,<br>2015<br>Korea<br>not clear	FBG 7.29 mmol/L<br>50 g GCT: 11.80 mmol/L	At screening: 6.75 ± 0.89 %<br><br>At delivery: 6.07 ± 0.58 %	6.23 ± 3.25	Fasting: 19.2 ± 9.0 mU/L	NR	NR	NR	NR	NR	NR	FPG: 4.39 mmol/L<br><br>50 g GCT 6.16 mmol/L	5.24 ± 0.34 %	2.2 ± 1.1	Fasting: 11.2 ± 5.3 mU/L	NR	NR	NR	NR
Wright L<br>2015<br>USA<br>not clear	NR	6.57 ± 0.82 mean throuh preg	NR	NR	NR	NR	NR	NR	NR	NR	NR	6.49 ± 0.79 throught whole preg	NR	NR	NR	NR	NR	NR
Abell S,<br>2016<br>Australia<br>retrospective cohort<br>study	NR	Median 1st trimester: 6.8%<br><br>Median 2nd trimester: 6.2%<br><br>Median 3rd trimester: 6.1%<br><br>Mean pregnancy HbA1c median: 6.4% (IQR 5.9–7.3)	NR	NR	NR	NR	NR	NR	NR	NR	NR	NR	NR	NR	NR	NR	NR	NR
Cade WT ,	6.1 ± 1.7	NR	NR	Basal	NR	NR	NR	NR	NR	NR	Basal: 81.2 ±	NR	NR	NR	NR	NR	NR	NR

2016 USA NR	mmol/L 2nd trim			32.3 ± 30.5 µU/mL<br><br>Hyperinsulinemia<br><br>61.4 ± 26.3 µU/mL (infused insulin).							10.5 mg/dL → 4.5 mmol/L Clamp: 90 mg/dL → 5.0 mmol/L							
Hammoud N, 2016 Netherlands NR	NR	preconceptional 6.7 ± 1.0 pregnancy 6.1 ± 0.8	NR	NR	NR	NR	NR	NR	NR	NR	NR	Preconceptional HbA1c (%) 7.1 ± 0.9 Pregnancy HbA1c (%) 6.3 ± 1.0	NR	NR	NR	NR	NR	NR
Villarroel C, 2016, Chile prospective study	Visit 1: 5.6 (4.0–12.3) Visit 2: 5.0 (3.6–8.0) Visit 3: 4.6 (3.6–8.0)	Visit 1: 5.9 (5.0–13.7) Visit 3: 6.3 (5.3–10.7) (No Visit 2 measurement.)	Visit 1: 4.2 (0.9–62.9) Visit 2: 4.5 (0.9–32) Visit 3: 4.5 (1.2–8.6)	Visit 1: 16.9 (4–196) Visit 2: 18.7 (5.2–144.4) Visit 3: 19.2 (4.8–42)	NR	NR	NR	NR	NR	NR	Visit 1: 4.0 (3.6–4.9) Visit 2: 4.3 (3.8–5.5) Visit 3: 4.2 (3.4–5.3)	Visit 1: 5.2 (4.8–6.0) Visit 3: 5.1 (4.3–5.7) (No Visit 2 measurement.)	Visit 1: 3.3 (0.2–17.5) Visit 2: 2.2 (0.7–3.3) Visit 3: 1.5 (0.6–5.5)	Visit 1: 17.8 (10.3–90.8) Visit 2: 11 (4.9–19.8) Visit 3: 8.5 (3.0–24.9)	NR	NR	NR	NR
Billionnet C , 2017 France cross-sectional study	NR	NR	NR	NR	NR	NR	NR	NR	NR	NR	NR	NR	NR	NR	NR	NR	NR	NR
Cade WT , 2017 USA Prospective study	NR	≤ 8 % before pregnancy	6.0 ± 6.2 (cord) at 32–36 wk	27.8 ± 24.1 at 32–36 wk	182 ± 94 mg/dL	178 ± 38 mg/dL	86 ± 34 mg/dL	57 ± 16 mg/dL	NR	3.2 ± 1.0	NR	NR	2.2 ± 1.7 2.8 ± 2.0 32–36 weeks	10.9 ± 6.9 11.8 ± 7.4 (cord) 32–36 weeks	89 ± 32 86 ± 29 32–36 weeks	57.7 ± 13.6 71.3 ± 17.7 32–36 weeks	NR	NR
Cnattingius S, 2017	NR	NR	NR	NR	NR	NR	NR	NR	NR	NR	NR	NR	NR	NR	NR	NR	NR	NR

Sweden<br>Not clear																		
Joshi T, 2017<br>Australia<br>not clear	6.5 mmol/L intrapartum	8.0 ± 2.7 %<br>pre-pregnancy<br>6.9 ± 1.4 %<br>1st trimester<br>6.2 ± 1.2 %.<br>2nd trimester<br>6.2 ± 1.3 %<br>3ed trimester	NR	NR	NR	NR	NR	NR	NR	NR	6.5 mmol/L intrapartum	8.5 ± 2.3 %<br>prepregnancy<br>7.8 ± 1.8 %<br>1st trimester<br>6.7 ± 1.2 %<br>2nd trimester<br>6.6 ± 1.1 %<br>3ed trimester	NR	NR	NR	NR	NR	NR
Ladfors L, 2017<br>Sweden<br>not clear	NR	44 ± 13 mmol/mol<br>1st trimester<br>36 ± 9 mmol/L<br>2nd trimester<br>36 ± 8 mmol/L<br>3ed trimester	NR	NR	NR	NR	NR	NR	NR	NR	NR	50 ± 13 mmol/mol<br>1st trimester<br>39 ± 12 mmol/L<br>2nd trimester<br>41 ± 10 mmol/L<br>3ed trimester	NR	NR	NR	NR	NR	NR
Saikia DM , 2017<br>Assam<br>prospective study	NR	6.34 ± 0.47% (3. trimes.)	NR	NR	3.69	6.76	3.73	1.15	NR	NR	NR	6.41 ± 0.42% (3. trimes.)	NR	NR	3.55	1.3	NR	NR
Villarroel C, 2017<br>Chile<br>prospective study	5.59 (4.04–12.28) fasting<br>2nd trimester	5.9 (5–13.7)<br>2nd trimester	4.2 (0.9–62.9)<br>2nd trimester	16.9 (4–196)<br>2nd trimester	NR	NR	NR	NR	NR	NR	4.49 (3.62–8.50) 2nd trimester<br>4.00 (3.60–4.92) 2nd trim	5.3 (4.7–6.3)<br>5.2 (4.8–6.0) 2nd trim	2.1 (0.4–20.4)<br>3.3 (0.2–17.5)<br>2nd trim	10.0 (2.2–86.9)<br>17.8 (10.3–90.8)<br>2nd trim	NR	NR	NR	NR
Alessi J, 2018<br>Brazil<br>Retrospective study	NR	7.6 ± 1.6<br><28 wk<br>6.4 ± 1.0<br>≥28 wk	NR	NR	NR	NR	NR	NR	NR	NR	NR	8.0 ± 1.8 %<br>< 28 weeks<br>7.2 ± 1.3 %<br>≥ 28 weeks	NR	NR	NR	NR	NR	NR
Endo S, 2018	NR	7.1 ± 1.6<br>prepregnancy	NR	NR	NR	NR	NR	NR	NR	NR	NR	7.6 ± 1.8<br>prepregna	NR	NR	NR	NR	NR	NR

Japan not clear		6.0 ± 0.5 (with complications) / 6.4 ± 1.7 (without) mean during pregnancy										ncy 7.2 ± 0.9 (with complications) / 6.0 ± 0.6 (without) mean during pregnancy						
Jang HJ , 2018 South Korea case–control study	NR	1st tri: 6.9 ± 1.5; 2nd: 5.7 ± 1.0; 3rd: 6.1 ± 0.8	NR	NR	NR	NR	NR	NR	NR	NR	NR	NR	NR	NR	NR	NR	NR	NR
Maple-Brown LJ , 2018 Australia prospective cohort study	FPG 6.1–6.4 mmol/L	6.5–6.6 % (early pregnancy)	NR	NR	NR	NR	NR	NR	NR	NR	FPG 6.5 ± 1.9 mmol/L FPG 4.6–4.9 mmol/L	7.9 ± 2.0 % 5.4–5.6 %	NR	NR	NR	NR	NR	NR
Scherneck S, 2018 Germany Prospective cohort study	NR	NR	NR	NR	NR	NR	NR	NR	NR	NR	NR	NR	NR	NR	NR	NR	NR	NR
Shimizu I, 2018 Japan Prospective study	NR	NR	NR	NR	NR	NR	NR	NR	NR	NR		NR	NR	NR	NR	NR	NR	NR
Agha-Jaffar R , 2019 United Kingdom case–control study	NR	6.8 (6.1–7.8)	NR	NR	NR	NR	NR	NR	NR	NR	FPG ≥ 6.1 mmol/L and/or 2 h ≥ 7.8 mmol/L at 24–28 weeks	5.6 (5.3 – 5.8) at diagnosis (24–28 weeks)	NR	NR	NR	NR	NR	NR
Bashir M, 2019 Qatar retrospective cohot study	NR	1st trimester: 7.3 ± 1.5 %; 3rd trimester: 6.7 ± 1.3 %	NR	NR	NR	NR	NR	NR	NR	NR	FPG < 5.1 mmol/L and 2-hour < 7.8 mmol/L	1st trimester 5.3 ± 0.4 %; 3rd trimester 5.4 ± 0.5 %.	NR	NR	NR	NR	NR	NR

												1st trimester: 6.3 ± 1.1 %; 3rd trimester: 5.9 ± 0.9 %						
Ásbjörnsdóttir B, 2019 Denmark Retrospective cohort study	NR	6.6 ± 1.2 %. Early pregnancy (first visit):	NR	NR	NR	NR	NR	NR	NR	NR	NR	6.6 ± 0.8 %. Early pregnancy:	NR	NR	NR	NR	NR	NR
Egan A, 2019 Republic of Ireland Retrospective study	NR	1st trimester: 44 (38–54) mmol/mol; 3rd trimester: 40 (37–44) mmol/mol.	NR	NR	NR	NR	NR	NR	NR	NR	NR	1st trimester: 58 (48–66) mmol/mol; 3rd trimester: 46 (41–51) mmol/mol.	NR	NR	NR	NR	NR	NR
Kong L, 2019 Finland not clear	NR	NR	NR	NR	NR	NR	NR	NR	NR	NR	NR	NR	NR	NR	NR	NR	NR	NR
Da Rocha Oppermann ML, 2019 Brazil Retrospective cohort study	NR	mean 7.3 ± 1.7% Early pregnancy	NR	NR	NR	NR	NR	NR	NR	NR	NR	mean 7.7 ± 1.4% Early pregnancy	NR	NR	NR	NR	NR	NR
Mackin S, 2019 United Kingdom retrospective cohort study	NR	First trimester: 6.9 ± 1.4%, third trimester: 6.2 ± 1.1%	NR	NR	NR	NR	NR	NR	NR	NR	NR	First trimester: 7.6 ± 1.5%, third trimester: 6.6 ± 1.2%	NR	NR	NR	NR	NR	NR
Stogianni A, 2019 Greece	NR	1st trimester: 6.9 ± 1.0%, 3rd trimester: 6.2 ± 0.7%	NR	NR	NR	NR	NR	NR	NR	NR	NR	1st trimester: 7.3 ± 1.1%, 3rd	NR	NR	NR	NR	NR	NR

Retroscpetive study												trimester: 6.3 ± 0.8%						
Wang X, 2019 USA<br><br>Retrospective cohort study	NR	NR	NR	NR	NR	NR	NR	NR	NR	NR	NR	NR	NR	NR	NR	NR	NR	NR
Wernimont ,S 2019 USA<br><br>Prospective cohort study	NR	7.6 ± 0.27% at enrollment; 6.3 ± 0.18% at delivery.	NR	NR	NR	NR	NR	NR	NR	NR	NR	5.3 ± 0.09% at enrollment; 5.1% at delivery. 5.3 ± 0.34% at enrollment; 5.4 ± 0.07% at delivery.	NR	NR	NR	NR	NR	NR
Ali D, 2020 Ireland<br><br>Retrospective study	NR	1st Trim 44.5 ± 9.4 mmol/mol (6.2%); 2nd 35.1 ± 5.8 mmol/mol (5.4%); 3rd 37.5 ± 6.4 mmol/mol (5.6%)	NR	NR	NR	NR	NR	NR	NR	NR	NR	1st Trim 56.3 ± 15.2 mmol/mol (7.3%); 2nd 42.4 ± 8.9 mmol/mol (6.0%); 3rd 44.2 ± 8.0 mmol/mol (6.2%)	NR	NR	NR	NR	NR	NR
López-de-Andrés A, 2020 Spain<br><br>Retrospective cohort study	NR	NR	NR	NR	NR	NR	NR	NR	NR	NR	NR	NR	NR	NR	NR	NR	NR	NR
Ásbjörnsdóttir B, 2020 Denmark Prospective cohort study	NR	6.9 ± 0.7% (52 ± 14 mmol/mol) 6.1 ± 0.6% (43 ± 8 mmol/mol)	NR	NR	NR	NR	NR	NR	NR	NR	NR	NR	NR	NR	NR	NR	NR	NR
Hauffe F,	NR	HbA1c 1st	NR	NR	NR	NR	NR	NR	NR	NR	NR	HbA1c	NR	NR	NR	NR	NR	NR

2020 German Retrospective cohort study		trimester (%) 6.3 (5.7, 7.2) HbA1c 3rd trimester (%) 6.1 (5.6, 6.6)										1st trimester (%) 6.5 (5.9, 7.2) HbA1c 3rd trimester (%) 6.1 (5.6, 6.6)						
Kattini R, 2020 Canada Retrospective study	NR	NR	NR	NR	NR	NR	NR	NR	NR	NR	NR	NR	NR	NR	NR	NR	NR	NR
Longmore D, 2020 Australia not clear	NR	NR	NR	NR	NR	NR	NR	NR	NR	NR	NR	NR	NR	NR	LDL	HDL	VLDL	Tg/HDL ratio
Morikawa M, 2020 Japan Retrospective cohort study	NR	First trimester: 7.30 ± 1.56%<br><br>At delivery: 6.19 ± 0.88%	NR	NR	NR	NR	NR	NR	NR	NR	NR	First trimester: 7.54 ± 1.16%<br><br>At delivery: 6.42 ± 0.76%	NR	NR	NR	NR	NR	NR
Starikov R, 2020 USA Retrospective cohort study	NR	(T2DM SGA): 7.7% (IQR 8.7–6.5) (T2DM AGA): 7.1% (IQR 8.7–6.3) 1st trimester	NR	NR	NR	NR	NR	NR	NR	NR	NR	T1DM SGA): 9.0% (10.7–7.8) (T1DM AGA): 8.1% (9.0–6.8)	NR	NR	NR	NR	NR	NR
Gualdani E, 2021 Italy not clear	NR	NR	NR	NR	NR	NR	NR	NR	NR	NR	NR	NR	NR	NR	NR	NR	NR	NR
Guarnotta V, 2021 Italy Retrospective cohort study	fasting 5.3 ± 0.8 mmol/L postprandial: 7.2 ± 1.1 mmol/L third trimester	6.8 ± 0.7% First trimester 6.3 ± 0.6% Second trimester 6.2 ± 0.5% Third trimester	NR	NR	NR	NR	NR	NR	NR	NR	fasting: 5.7 ± 1.1 mmol/L postprandial: 8.1 ± 1.6 mmol/L third trimester	7.6 ± 1.2% First trimester 6.9 ± 0.9% Second trimester 6.8 ± 0.8% Third	NR	NR	NR	NR	NR	NR

												trimester						
Martínez-Cruz C, 2021 Mexico Prospective study	NR	NR	NR	NR	NR	NR	NR	NR	NR	NR	NR	NR	NR	NR	NR	NR	NR	NR
McLean A, 2021 Australia not clear	NR	NR	NR	NR	NR	NR	NR	NR	NR	NR	NR	NR	NR	NR	NR	NR	NR	NR
Saquib S, 2021 United Arab Emirates Retrospective study	NR	Prepregnancy: 6.8 ± 1.5<br><br>1st trimester: 6.4 ± 1.3<br><br>2nd trimester: 5.9 ± 0.9<br><br>3rd trimester: 6.2 ± 1.1	NR	NR	NR	NR	NR	NR	NR	NR	NR	Prepregnancy: 7.6 ± 1.6<br><br>1st trimester: 6.6 ± 1.1<br><br>2nd trimester: 6.1 ± 0.9<br><br>3rd trimester: 5.9 ± 1.1	NR	NR	NR	NR	NR	NR
Seah JM, 2021 Australia Retrospective study	NR	6.8 ± 1.2%<br>1st trimester	NR	NR	NR	NR	NR	NR	NR	NR	NR	NR<br>7.3 ± 1.3%<br>1st trimester	NR	NR	NR	NR	NR	NR
Britten F, 2022, Australia Not clear	NR	5.5 ± 0.4 %, treći trimestrar	NR	NR	NR	NR	NR	NR	NR	NR	NR	NR	NR	NR	NR	NR	NR	NR
Rao C, 2022 China Prospective cohort study	NR	6.46 ± 1.11 1st trimester<br>5.53 ± 0.45 2nd and 3rd trim	NR	NR	NR	NR	NR	NR	NR	NR	NR	6.93 ± 1.82 1st trim<br>5.30 ± 0.76 2nd and 3rd trim<br>GDM<br>5.37 ± 0.37<br>5.34 ± 0.56	NR	NR	NR	NR	NR	NR
Jacobsen D, 2022 Norway	NR	6.3<br>3rd trimester	NR	NR	NR	NR	NR	NR	NR	NR	NR	5.2<br>6.4<br>5.8	NR	NR	NR	NR	NR	NR

not clear												3rd trimester						
Kapustin R, 2022 Russia Retrospective cohort study	NR	NR	NR	NR	NR	NR	NR	NR	NR	NR	NR	NR	NR	NR	NR	NR	NR	NR
Kapustin R, 2022a Russia Prospective cohort study	NR	1st trimester Diet 6,0 (5.7–6.3) Insulin 6.8 (6.4–7.3) 2nd trimester Diet 5.8 (5.1–6.4) Insulin 6.5 (6.0–7.1) 3rd trimester Diet 5.4 (5.2–5.7) Insulin 5.9 (5.2–6.5)	NR	NR	NR	NR	NR	NR	NR	NR	NR							
Cordero L, 2022 USA Retrospective cohort study	NR	NR	NR	NR	NR	NR	NR	NR	NR	NR	NR	NR	NR	NR	NR	NR	NR	NR
Cordero L, 2022 USA Retrospective cohort study	NR	NR 2–3. trimestrar	NR	NR	NR	NR	NR	NR	NR	NR	NR	NR 2–3. trimester	NR	NR	NR	NR	NR	NR
Malaza N, 2023 South Africa Prospective study	NR	7.6 (6.3–9.3) 2nd trimester	NR	NR	NR	NR	NR	NR	NR	NR	NR	NR 9.7 (9.0–11.0) 5.7 (5.4–6.1)	NR	NR	NR	NR	NR	NR
Koyama M, 2023 Japan Not clear	NR	6.7% (5.8–8.6) pre trudnoće; 6.2% (5.5–7.9) u 3. trimestru	NR	NR	NR	NR	NR	NR	NR	NR	NR	7.2% (6.1–10.0) pre-pregnancy; 6.7% (6.3–8.4) 3rd trimester	NR	NR	NR	NR	NR	NR
Powers Carson J,	NR	NR	NR	NR	NR	NR	NR	NR	NR	NR	NR	NR	NR	NR	NR	NR	NR	NR

2024<br>USA<br>case-control study																		
Kapustin R, 2024<br>Russia<br>retrospective cohort study	NR	Preconception: 7.5%<br>1st trimester: 7.3%<br>2nd: 6.8%<br>3rd: 6.8%	NR	NR	NR	NR	NR	NR	NR	NR	NR	Preconception 8.1<br>1st tri 7.8<br>2nd tri 6.9<br>3rd tri 6.9<br>GDM - 5.6 1st trimester	NR	NR	NR	NR	NR	NR
Ballesteros M, 2024<br>Spain<br>prospective cohort study	NR	Pregestational: 7.2 ± 1.7<br><br>1st trimester: 6.6 ± 1.3<br><br>2nd trimester: 5.7 ± 0.6<br><br>3rd trimester: 5.8 ± 0.7	NR	NR	NR	NR	NR	NR	NR	NR	NR	Pregestational: 7.1 ± 1.3<br><br>1st trimester: 6.6 ± 1.1<br><br>2nd trimester: 5.9 ± 0.6<br><br>3rd trimester: 6.1 ± 0.6	NR	NR	NR	NR	NR	NR
Grazia Dalfrà M, 2024<br>Italy<br>retrospective study	NR	7.3 ± 2.2<br>at 1st trimester	NR	NR	NR	NR	NR	NR	NR	NR	NR	9.7 ± 3.1<br>1st trimester	NR	NR	NR	NR	NR	NR
Suzuki T, 2024<br>Japan<br>retroscpetcive study	NR	Mean HbA1c 1st trimester (%) 6.6 ± 1.0<br>Mean HbA1c 2nd trimester (%) 6.2 ± 0.8<br>Mean HbA1c 3rd trimester (%) 6.3 ± 0.8		NR	1st tri: 101 ± 53 mg/dL<br>3rd tri: 259 ± 130 mg/dL	1st tri: 169 ± 34<br>3rd tri: 249 ± 46	NR	NR	NR	NR	NR	Mean HbA1c 1st trimester (%) 7.1 ± 0.9<br>Mean HbA1c 2nd trimester (%) 6.4 ± 0.8<br>Mean HbA1c 3rd trimester		NR	NR	NR	NR	NR

												(%) 6.5 ± 0.9						
Dias S, 2025 South Africa Not clear	T2DM - NR New T2DM 0-h OGTT (mmol/L) 7.5 (6.7–9.9) 1-h OGTT (mmol/L) 12.8 (12.1–14.9) 2-h OGTT (mmol/L) 12.6 (11.2–15.9) 2nd trimester	7.7 (6.5–9.3) T2DM 6.9 (5.9–8.7) New T2DM 2nd trimester	NR	T2DM (preexisting) 19.3 (3.4–40.4) New T2DM in pregnancy 8.9 (3.5–25.0) 2nd trimester	327.3 (233.0–698.5) T2DM 416.5 (284.9–554.9) New T2DM	NR	NR	NR	NR	NR	T1DM - NR Controls 0-h OGTT (mmol/L) 3.9 (3.7–4.3) 1-h OGTT (mmol/L) 5.6 (4.4–6.8) 2-h OGTT (mmol/L) 4.5 (5.1–6.5) 2nd trimester GDM 0-h OGTT (mmol/L) 5.4 (5.1–6) 1-h OGTT (mmol/L) 9.9 (8.2–11) 2-h OGTT (mmol/L) 8.8 (6.7–9.5)	5.2 (5.0–5.4) Controls 9.3 (7.6–10.1) T1DM 5.7 (5.4–6.1) GDM	NR	Controls 6.9 (1.4–29.1) T1DM 22.1 (5.8–37.9) GDM 21.7 (4.8–54.6) 2nd trimester	NR	NR	NR	NR
Gherbon A, 2025 Romania Retrospective study	8.14 mmol/L 2nd trimester	7.18 ± 2.00 2nd trimester	NR	NR	NR	NR	NR	NR	NR	NR	CTRL: 4.27 mmol/L GDM: 5.50 mmol/L T1DM: 10.03 mmol/L 2nd trimester	4.57 ± 0.33 5.45 ± 0.54 8.23 ± 2.44 2nd trimester	NR	NR	NR	NR	NR	NR
Hillick D, 2025 Ireland Retrospective cohort study	NR	NR	NR	NR	NR	NR	NR	NR	NR	NR	NR	NR	NR	NR	NR	NR	NR	NR
Manga J, 2025 South Africa Retrospective study	NR	7.8% 2nd trimester	NR	NR	NR	NR	NR	NR	NR	NR	NR	8.8% 5.7% 7.7% 2nd trimester	NR	NR	NR	NR	NR	NR
Zhou X, 2025 China retrospective cohort study	6.61 2nd trimester	5.69 ± 0.63 2nd trimester	7.24 2nd trimester	fasting 16.75 ± 7.40 2h 110.62 ± 64.69 2nd trimester	2.79 ± 2.10	4.44 ± 1.33	2.62 ± 0.75	1.38 ± 0.36	NR	NR	T1DM: 7.23 ODM: 6.79 GDMA1: 4.46 GDMA2: 4.53 2nd trimester	T1DM: 6.07 ± 0.85 ODM: 5.80 ± 0.78	T1DM: 2.47 ODM: 7.24 GDMA1: 1.01 GDMA2: 2.32 2nd trimester	fasting T1DM: 7.55 ± 1.96 ODM: 24.18 ± 9.97 GDMA1: 5.07	T1DM: 2.47 ± 0.9	T1DM: 1.33 ± 0.3	NR	NR

												GDMA1: 5.04 ± 0.38<br>GDMA2: 5.36 ± 0.37<br>2nd trimester		± 1.72<br>GDMA2: 11.43 ± 3.62<br>2h insulin<br>T1DM: 6.73 ± 1.59<br>ODM: 202.76 ± 59.02<br>GDMA1: 54.09 ± 29.96<br>GDMA2: 91.62 ± 46.00<br>2nd trimester	1<br>ODM: 2.49 ± 0.77<br>GDMA1: 2.80 ± 0.95<br>GDMA2: 2.90 ± 0.95	1<br>ODM: 1.36 ± 0.37<br>GDMA1: 1.63 ± 0.34<br>GDMA2: 1.60 ± 0.32		
