# Supplementary material for: Body Weight-Related Parameters in Pregnancies Complicated by Type 2 Diabetes Mellitus: A Systematic Review and Meta-Analysis with Maternal and Perinatal Outcome Mapping
Source: J Clin Med. 2026 Jul 6;15(13):5260. doi: 10.3390/jcm15135260 (PMC13362816; doi:10.3390/jcm15135260)
Supplement: Supplementary file 1 [file jcm-15-05260-s001.zip › Supplementary Table S5a. Neonatal adverse outcomes.pdf]

Supplement material. Table S5a. Neonatal adverse outcomes

Author, year Country Study design	NEONATAL OUTCOMES CASES																	
	Premature delivery Yes/No (weeks of gestation)	Fetal/Neonatal loss Yes/No	Congenital abnormalities Yes/No (which one)	Small for gestational age SGA Yes/No	Large for gestational age LGA Yes/No	Macrosomia Yes/No	IUFD	Injury (Cranial, brachial, clavicule)	Sepsis	Intubation	Death	Hypertrophic miocardiopathy Yes/No	NICU admission	Respiratory distress syndrome Yes/No	Neonatal hypoglycemia Yes/No	Hypocalcemia Yes/No	Hyperbilirubinemia Yes/No	Polycytemia Yes/No
Barnard R, 1997 Australia, Not clear	Yes, mean 35.5 ± 1.7 wk	No	NR	1 (13%)	2 (25%)	2 (25%)	1 (13%)	NR	NR	NR	NR	NR	NR	NR	NR	NR	NR	NR
Ben Slama C, 1997 Tunis Retrospective study	Yes	Yes	Yes 2/45 (≈4.4%)	NR	NR	Yes	Yes	NR	Yes	NR	Yes	NR	Yes	Yes	Yes	NR	NR	NR
Colatrella A, 2009 Italy Not clear	Yes — 18.4%	Yes, 1	Yes — 7.9% (6/76)	NR	NR	NR	NR	NR	NR	NR	NR	NR	NR	NR	NR	NR	NR	NR
Contreras-Soto J, 1991 Mexico NR	Yes 5.93% (7/118)	2.54% (3/118)	5.08% (6/118)	2.54% (3/118)	29.66% (35/118)	NR	NR	NR	0.86% (1/118)	NR	NR	NR	NR	4.24% (5/118)	5.08% (6/118)	NR	4.24% (5/118)	NR
Cundy T, 2000 New Zealand Not clear	16 (23%)	2 (2.8%)	4 (5.6%)	2 (2.8%)	18 (25%)	9 (12.7%)	NR	NR	NR	NR	NR	NR	21 (30%)	5 (7%)	14 (19.7%)	NR	NR	NR
Cundy T, 2002 New Zealand NR	9.8% (n=4)	NR	NR	14.6% (n=6)	29.3% (n=12)	NR	NR	NR	NR	NR	NR	NR	47.5% (n=19)	NR	NR	NR	NR	NR
Cundy T, 2007 New Zealand Not clear	NR	19 (3.4%)	NR	NR	NR	NR	NR	NR	NR	NR	NR	NR	NR	NR	NR	NR	NR	NR
Falhammar H, 2010 Australia Not clear	NR	No	No	NR	NR	NR	NR	18%	NR	NR	NR	NR	NR	22%	NR	NR	NR	NR
Lapolla A, 2008 Italy Prospective study	33.6 %	Stillbirth 1.9 % (3); Neonatal 1.9 % (3)	2.0 % (3)	NR	11.8 % (17)	NR	NR	1.3 % (2)	NR	NR	3 neonatal (1.9 %)	NR	NR	3.5 % (5)	19.5 % (28)	6.3 % (9)	15.3 % (22)	NR
Marin J 2010, Romania Retrospective study	Yes, lower mean GA 36.97 wks	NR	NR	NR	NR	NR	NR	Cranial hematoma 10% Clavicule fracture 6% / 0.8%	NR	NR	NR	NR	NR	9%	NR	71%	16%	NR
Min Y, 2005 UK NR	Yes – 1/17 (5.9 %)	NR	NR	No	NR	Yes – 1 case	NR	NR	NR	NR	NR	NR	NR	NR	NR	NR	NR	NR
Abrão Szylit N, 2007 Brazil Cross-sectional study	Yes	Yes → 3/48 (6.3%)	Yes → 5/48 (10.4%)	NR	NR	NR	Yes	NR	NR	NR	NR	Yes	NR	NR	NR	NR	NR	NR
Gonzalez-Gonzalez NL., 2008, Spain retrospective study	18.6% (27/145) • Mean GA 38 w	4 cases total = 2.75% (2 fetal, 2 neonatal)	6.8% (10/145) • Types NR	2.2% (3/145)	28.8% (40/145)	13.8% (20/145)	2 neonatal deaths (part of perinatal mortality)	NR	NR	NR	NR	NR	15.2% (22/145)	NR	NR	NR	NR	NR
Olmos PR, 2009, Chile NR	Yes — 11/51 (21.56%); mean GA 37.33 wks overall	2/51 (3.92%)	2/51 (Fallot's tetralogy, fatal complex heart malformation)	2/51 (3.92%)	19/51 (37.25%)	7/51 (13.72 %)	2/51 (3.92%)	NR	NR	NR	NR	NR	10/51 (19.6%)	NR	4/51 (7.84%)	NR	5/51 (9.8%)	NR
Roland JM, 2005 UK prospective study	NR	6.20%	12.3% (18/146)	9.60%	46.90%	NR	NR	NR	NR	NR	NR	NR	NR	NR	NR	NR	NR	NR
Westgate JA, 2006, New Zealand Not clear	NR	NR	NR	NR	NR	13/39 = 33%	NR	NR	NR	NR	NR	NR	NR	NR	NR	NR	NR	NR
Yang X, 2002 China Prospective study	Yes: 7.84% (8/102)	1.96% (2/102)	NR	NR	21.6% (22/102)	12.8% (13/102)	NR	NR	1.96% (2/102)	NR	NR	NR	NR	NR	0.98% (1/102)	NR	NR	NR
Cyganek K, 2011 Poland	Preterm deliveries: 12.7%	1.6% (1/63)	7.9% (5/63)	NR	NR	7.9% (5/63)	NR	NR	NR	NR	NR	NR	NR	NR	NR	NR	NR	NR

Author, year Country Study design	NEONATAL OUTCOMES CONTROLS																	
	Premature delivery Yes/No (weeks of gestation)	Fetal/Neonatal loss Yes/No	Congenital abnormalities Yes/No (which one)	Small for gestational age Yes/No	Large for gestational age Yes/No	Macrosomia Yes/No	IUFD	Injury (Cranial, brachial, clavicule)	Sepsis	Intubation	Death	Hypertrophic miocardiopathy Yes/No	NICU admission	Respiratory distress syndrome Yes/No	Neonatal hypoglycemia Yes/No	Hypocalcemia Yes/No	Hyperbilirubinemia Yes/No	Polycytemia Yes/No
Barnard R, 1997 Australia, Not clear	Yes, mean 36.9 ± 0.4 wk	No	NR	2 (5%)	4 (11%)	No	No	NR	NR	NR	NR	NR	NR	NR	NR	NR	NR	NR
Ben Slama C, 1997 Tunis Retrospective study	Yes	Yes	Yes	NR	NR	Yes	Yes	NR	Yes	NR	Yes	NR	Yes	Yes	Yes	NR	NR	NR
Colatrella A, 2009 Italy Not clear	Yes 1.6%	NR	NR	NR	NR	NR	NR	NR	NR	NR	NR	NR	NR	NR	NR	NR	NR	NR
Contreras-Soto J, 1991 Mexico NR	5 (5.43%)	5 (5.43%)	7 (7.60%)	1 (1.11%)	31 (33.78%)	NR	NR	NR	NR	NR	NR	NR	NR	3 (3.26%)	NR	NR	1 (1.09 %)	NR
Cundy T, 2000 New Zealand Not clear	40 (40%)	3 (3%)	6 (6%)	6 (6%)	30 (30%)	14 (14%)	NR	NR	NR	NR	NR	NR	44 (44%)	10 (10%)	35 (35%)	NR	NR	NR
Cundy T, 2002 New Zealand NR	22.2% (n=10)	NR	NR	No	44.4% (n=20)	NR	NR	NR	NR	NR	NR	NR	75.6% (n=34)	NR	NR	NR	NR	NR
Cundy T, 2007 New Zealand Not clear	NR	9 (2.7%)	NR	NR	NR	NR	NR	NR	NR	NR	NR	NR	NR	NR	NR	NR	NR	NR
Falhammar H, 2010 Australia Not clear	NR	No	No	NR	NR	NR	NR	18%	NR	NR	NR	NR	NR	4%	4%	NR	26%	NR
Lapolla A, 2008 Italy Prospective study	37.5 %	Stillbirth 1.06 % (5); Neonatal 0.21 % (1)	5.9 % (28)	NR	13.2 % (62)	NR	NR	Obstetric trauma 1.8 % (8)	NR	NR	1 neonatal (0.21 %)	NR	NR	1.5 % (7)	16.4 % (77)	8.1 % (38)	22.4 % (105)	NR
Marin J 2010, Romania Retrospective study	NR	NR	NR	NR	NR	NR	NR	NR	NR	NR	NR	NR	NR	NR	NR	47%	20%	NR
Min Y, 2005 UK NR	NR	NR	NR	NR	NR	NR	NR	NR	NR	NR	NR	NR	NR	NR	NR	NR	NR	NR
Abrão Szylit N, 2007 Brazil Cross-sectional study	Yes<br>NR	Yes → 3/125 (2.4%)<br>No	Yes → 7/125 (5.6%)<br>NR	NR<br>NR	NR<br>NR	NR<br>NR	Yes<br>No	NR<br>NR	NR<br>NR	NR<br>NR	NR<br>NR	Yes<br>NR	NR<br>NR	NR<br>NR	NR<br>NR	NR<br>NR	NR<br>NR	NR<br>NR
Gonzalez-Gonzalez NL., 2008, Spain retrospective study	24.2% (64/253) • Mean GA 37.4 w	1.97% (3 fetal, 2 neonatal)	6.22% (16/257)	2.9% (7/253)	35.1% (84/253)	20.6% (52/253)	5 total (1.97%)	NR	NR	NR	NR	NR	10.7% (27/253)	NR	NR	NR	NR	NR
Olmos PR, 2009, Chile NR	Yes 9/23 (39.13%)	2/51 (3.92%)	2 Down syndrome cases	0%	6/23 (26.08%)	1/23 (4.34 %)	NR	NR	NR	NR	NR	NR	9/23 (39.1%)	NR	3/23 (13.04 %)	NR	4/23 (17.39 %)	NR
Roland JM, 2005 UK prospective study	NR	2.80%	4.4% (17/389)	3.60%	46.50%	NR	NR	NR	NR	NR	NR	NR	NR	NR	NR	NR	NR	NR
Westgate JA, 2006, New Zealand Not clear	NR	NR	NR	NR	NR	NR	NR	NR	NR	NR	NR	NR	NR	NR	NR	NR	NR	NR
Yang X, 2002 China Prospective study	1.32% (4/302)	0.66% (2/302)	NR	1.66% (5/302)	13.3% (40/302)	5.96% (18/302)	NR	NR	NR	NR	NR	NR	NR	NR	0.33% (1/302)	NR	NR	NR
Cyganek K, 2011 Poland	Preterm deliveries: 17.8%	2.2% (7/320)	6.6% (21/320)	7.5% (24/320)	NR	17.5% (56/320)	NR	NR	NR	NR	NR	NR	NR	NR	NR	NR	NR	NR

NR	(8/63)<br>Early preterm (<34 weeks): 4.7% (3/63)<br>Mean gestational age overall: 38.1 weeks.																		(57/320).<br><br>Early preterm (<34 weeks): 4.1% (13/320).																	
Handisurya A, 2011<br>Austria<br>Not clear	15/60 = 25.4%	7/66 = 10.6%	Major cardiac: 2 (3.4%)<br>Major non-cardiac: 1 (1.6%)	7/60 = 11.9%	16/60 = 27.1%	13/60 = 21.7%	NR	1 (3.5%)	NR	NR	1 (1.5%)	NR	15/60 = 25.0%	NR	4/60 = 7.3%	NR	22/60 = 37.9%	NR	14/59 = 23.7%	17/75 = 22.7%	Major cardiac: 3 (5.0%)<br>Major non-cardiac: 1 (1.7%)	4/59 = 6.8%	10/59 = 17.0%	5/59 = 8.5%	NR	NR	NR	NR	NR	NR	15/59 = 25.4%	NR	4/59 = 7.4%	NR	15/59 = 26.8%	NR
Knight K, 2012<br>USA<br>Retrospective cohort study	51<br>23.9%	NR	20<br>9.4%	15<br>7.1%	73<br>34.4%	NR	4<br>1.9%	4<br>1.9%	9<br>4.2%	18<br>8.5%	3<br>1.4%	NR	176<br>86.3%	15<br>7.0%	33<br>15.5%	NR	64<br>30.0%	NR	21<br>9.9%	NR	9<br>4.2%	21<br>9.9%	23<br>10.8%	NR	0<br>0%	NR	NR	NR	3<br>1.4%	NR	36<br>17.3%	6<br>2.8%	3<br>1.4%	NR	28<br>13.1%	NR
Murphy H, 2011<br>UK<br>Prospective cohort study	<37 weeks: 17.5% (38/217)<br><br><34 weeks: 3.7% (8/217)	Stillbirth: 2/222 (9/1000 births)	9/ (41 per 1000 births)	11.4% (24/210)	37.6% (79/210)	NR	NR	NR	NR	NR	NR	NR	11 (5.0%)	NR	NR	NR	NR	NR	<37 weeks: 37.1% (120/322)<br><br><34 weeks: 7.1% (23/322)	Stillbirth: 5/330<br><br>Neonatal death: 3/330	14 (42/1000 births)	4.9% (15/308)	52.9% (163/308)	NR	NR	NR	NR	NR	NR	NR	31 (9.8%)	NR	NR	NR	NR	NR
de Oliveira Baraldi C, 2012<br>Brazil<br>not clear	NR	NR	NR	NR	NR	NR	NR	NR	NR	NR	NR	NR	NR	NR	NR	NR	NR	NR	NR	NR	NR	NR	NR	NR	NR	NR	NR	NR	NR	NR	NR	NR	NR	NR	NR	NR
Higgins M, 2013<br>Ireland<br>Prospective cohort study	No	NR	NR	NR	NR	3/10 (30%)	NR	NR	NR	NR	NR	NR	1/10 (10%)	NR	NR	NR	NR	NR	No	NR	NR	NR	NR	5/30 (16.7 %)	NR	NR	NR	NR	NR	NR	1/30 (3.3%)	NR	NR	NR	NR	NR
Min Y, 2014<br>United Kingdom<br>RCT	12/30 (40.0%)	0%	NR	NR	NR	NR	NR	NR	NR	NR	NR	NR	NR	NR	NR	NR	NR	NR	3/27 (11.1%)	1	NR	NR	NR	NR	NR	NR	NR	NR	NR	NR	NR	NR	NR	NR	NR	NR
Sato T, 2014<br>Japan<br>Retrospective study	102<br>17.6%	3<br>0.52%	24<br>4.1%	91<br>15.7%	190<br>32.8%	29<br>5.0%	2<br>0.34%	NR	NR	NR	NR	NR	229<br>39.6%	62<br>10.7%	78<br>13.5%	13<br>2.2%	89<br>15.4%	12<br>2.1%	55<br>14.9%	2<br>0.54%	17<br>4.6%	43<br>11.7%	112<br>30.2%	17<br>4.6%	2<br>0.54%	NR	NR	NR	NR	NR	123<br>33.3%	34<br>9.2%	45<br>12.2%	3<br>0.8%	56<br>15.2%	7<br>1.9%
Hall D, 2015,<br>South Africa<br>Not clear	NR	NR	NR	NR	NR	9.50%	5		NR	NR	NR	NR	1	NR	NR	NR	NR	NR	NR	NR	NR	NR	NR	NR	NR	NR	NR	NR	NR	NR	NR	NR	NR	NR	NR	NR
Huynh J, 2015<br>USA<br>not clear	NR	NR	NR	NR	NR	NR	NR	NR	NR	NR	NR	NR	NR	NR	NR	NR	NR	NR	NR	NR	NR	NR	NR	NR	NR	NR	NR	NR	NR	NR	NR	NR	NR	NR	NR	NR
Owens L, 2015<br>Ireland<br>case-control study	24<br>22%	NR	3<br>2.8%	5.60%	20%	NR	NR	NR	NR	NR	2<br>1.9%	NR	42<br>39%	NR	7<br>6.5%	NR	9<br>8.3%	1<br>0.9%	60<br>28%	NR	9<br>4.2%	5.60%	24%	30%	NR	NR	NR	NR	6<br>2.8%	NR	55%	NR	43<br>20%	NR	13<br>6%	2<br>0.9%
Park S, 2015<br>Korea<br>not clear	6/71 (8.2%)	NR	NR	2/71 (3.39%)	11/71 (15.3%)	NR	NR	NR	NR	NR	NR	NR	NR	NR	NR	NR	NR	NR	6/71 (8.2%)	NR	NR	28/463 (5.95%)	26/463 (5.71%)	NR	NR	NR	NR	NR	NR	NR	NR	NR	NR	NR	NR	NR
Wright L 2015<br>USA<br>not clear	NR	NR	8.10%	NR	19%	NR	NR	NR	NR	NR	NR	NR	NR	NR	51%	NR	NR	NR	NR	NR	8.30%	NR	33%	NR	NR	NR	NR	NR	NR	NR	NR	NR	58%	NR	NR	NR
Abell S, 2016<br>Australia<br>retrospective cohort study	22.5% (adjusted OR 2.74)	2.2% (not significantly ↑)	4.4%, no ↑ vs controls	15.90%	20.30%	14.5% (NS)	NR	shoulder dystocia 7.8% of vaginal births	NR	NR	2.20%	NR	7.40%	7.3% (NS)	22.50%	NR	20.30%	NR	8.10%	NR	NR	14.70%	7.70%	NR	NR	shoulder dystocia 2.5%	NR	NR	NR	NR	2.70%	3.80%	4.00%	NR	6.40%	NR
Cade WT , 2016<br>USA<br>NR	Yes; mean GA = 37.0 ± 1.9 weeks	NR	NR	NR	NR	NR	NR	NR	NR	NR	NR	NR	8% NICU	NR	NR	NR	NR	NR	NR	NR	NR	NR	NR	NR	NR	NR	NR	NR	NR	NR	NR	NR	NR	NR	NR	NR
Hammoud N, 2016<br>Netherlands<br>NR	NR	NR	NR	NR	43%	NR	NR	NR	NR	NR	NR	NR	48%	NR	NR	NR	NR	NR	NR	NR	NR	NR	47%	NR	NR	NR	NR	NR	NR	NR	78%	NR	NR	NR	NR	NR
Villarroel C, 2016,<br>Chile<br>prospective study	NR	NR	NR	NR	NR	NR	NR	NR	NR	NR	NR	NR	NR	NR	NR	NR	NR	NR	NR	NR	NR	NR	NR	NR	NR	NR	NR	NR	NR	NR	NR	NR	NR	NR	NR	NR
Billionnet C , 2017<br>France<br>cross-sectional study	19 % (OR 3.1 [2.7–3.4])	Yes	2.8 % T2DM Cardiac malformations<br>0.42 % T2DM Nervous system malformations	NR	LGA included under macrosomia	Yes<br>28.9 %	Yes	1.5 % Erb's palsy/clavicle fracture	NR	NR	2.4 %	NR	NR	7.3 %	NR	NR	NR	NR	No DM 7.0 %<br>GDM 8.4 % (OR 1.2 [1.2–1.3])	Yes	0.7 % no DM<br>0.9 % GDM<br>Cardiac malformations<br>0.15 % no DM<br>0.12 % GDM<br>Nervous system malformations	NR	LGA included under macrosomia	Yes<br>No DM 9.2 %<br>GDM 15.7 % (OR 1.8 [1.7–1.8])	Yes	No DM 0.5 %<br>GDM 0.7 %<br>Erb's palsy/clavicle fracture	NR	NR	0.6 % no DM<br>0.5 % GDM	NR	NR	2.9 % no DM<br>3.6 % GDM	NR	NR	NR	NR
Cade WT , 2017<br>USA<br>Prospective study	Yes	No	No	No	Yes	Yes	No	NR	NR	NR	NR	No	NR	No	NR	NR	NR	NR	No<br>No	No<br>No	No<br>No	No<br>No	No<br>No	No<br>No	No<br>No	NR<br>NR	NR<br>NR	NR<br>NR	No<br>No	No<br>No	NR<br>NR	No<br>No	NR<br>NR	NR<br>NR	NR<br>NR	NR<br>NR
Cnattingius	Yes	NR	No	Yes	Yes	29.00%	NR	NR	NR	NR	NR	No	NR	No	NR	NR	NR	NR	Yes	NR	No	6.2%	35.4%	29.0%	NR	NR	NR	NR	NR	No	NR	No	NR	NR	NR	NR

S, 2017 Sweden Not clear	13.0 %			4.5%	35.4%														4.5 % 19.7 %			2.8%	51.7%	36.1%												
Joshi T, 2017 Australia not clear	≈ 50 %	NR	NR	NR	median birthweight centile 80 [46–96]	NR	NR	NR	NR	NR	NR	NR	89 %	NR	40 %	NR	NR	NR	≈ 45 %	NR	NR	NR	median birthweight centile 90 [68–97]	NR	NR	NR	NR	NR	NR	NR	90 %	NR	50 %	NR	NR	NR
Ladfors L, 2017 Sweden not clear	Yes 13%	No	No	NR	Yes 20 (23%)	Yes 19 (22%)	No	Yes 1 (1%)	NR	NR	No	NR	NR	NR	Yes 11 (13%)	NR	NR	NR	Yes 29%	No	No	NR	Yes 111 (50%)	Yes 87 (39%)	No	Yes 4 (2%)	NR	NR	No	NR	NR	NR	Yes 61 (28%)	NR	NR	NR
Saikia DM , 2017 Assam prospective study	NR	NR	NR	NR	NR	NR	NR	NR	NR	NR	NR	NR	NR	NR	NR	NR	NR	NR	NR	NR	NR	NR	NR	NR	NR	NR	NR	NR	NR	NR	NR	NR	NR	NR	NR	NR
Villarroel C, 2017 Chile prospective study	NR	NR	NR	NR	NR	NR	NR	NR	NR	NR	NR	NR	NR	NR	NR	NR	NR	NR	NR	NR	NR	NR	NR	NR	NR	NR	NR	NR	NR	NR	NR	NR	NR	NR	NR	NR
Alessi J, 2018 Brazil Retrospective study	Yes 29%	Yes 6%	Yes – 6%	NR	Yes	Yes 17%	Yes – 17%	NR	NR	NR	Yes 0.8%	NR	Yes 41%	NR	Yes 17%	NR	Yes 12%	NR	Yes 53%	Yes 16%	Yes 7%	NR	Yes	Yes 19%	Yes	NR	NR	NR	Yes	NR	Yes 77%	NR	Yes 33%	NR	Yes 26%	NR
Endo S, 2018 Japan not clear	NR	No	Yes 1	Yes 1	Yes 1	NR	NR	NR	NR	NR	NR	NR	NR	NR	Yes 2	NR	NR	NR	Yes 1	No	NR	NR	Yes 6	NR	NR	NR	NR	NR	NR	NR	NR	NR	Yes 5	NR	NR	NR
Jang HJ , 2018 South Korea case–control study	Yes 9 %	No	Yes 2 cases (2%) – both caudal regression syndrome	Yes 7 %	Yes 22 %	Yes 15 %	No	Yes 3% cranial injury; 2% clavicle fracture	NR	NR	No	NR	Yes 14 %	Yes 40 %	Yes 4 %	Yes 3 %	Yes 33 %	NR	Yes 10 %	No	No	Yes 5 %	Yes 9 %	Yes 5 %	No	Yes 1% cranial injury; 1% clavicle fracture	NR	NR	NR	NR	Yes 13 %	Yes 28 %	No	No	Yes 21 %	NR
Maple-Brown LJ , 2018 Australia prospective cohort study	Yes 35.5 % Indig / 17 % non-Indig	NR	NR	Yes 6.5 % Indig / 0 % non-Indig	Yes 37.5 % Indig / 21.7 % non-Indig	Yes	NR	NR	NR	NR	NR	NR	NR	NR	NR	NR	NR	NR	Yes 14.7 % Indig / 9 % non-Indig 16.8 % Indig / 9 % non-Indig	NR	NR	Yes 8.1–8.7 % 8–11 %	Yes 36 % Indig / 21.7 % non-Indig 19–20 %	Yes	NR	NR	NR	NR	NR	NR	NR	NR	NR	NR	NR	NR
Scherneck S, 2018 Germany Prospective cohort study	Yes 16.2 % (51/314)	Yes HR 1.05 (0.3–4.0)	Yes – 3.5 %	NR	NR	NR	NR	NR	NR	NR	NR	NR	NR	NR	NR	NR	NR	NR	Yes – 11.2 %	Yes	Yes – 4.2 %	NR	NR	NR	NR	NR	NR	NR	NR	NR	NR	NR	NR	NR	NR	NR
Shimizu I, 2018 Japan Prospective study	No	NR	NR	NR	NR	NR	NR	NR	NR	NR	NR	No	NR	Yes 9.5 %	Yes – 18.7 %	Yes – 2.5 %	Yes – 20 %	Yes – 6 %	No	NR	NR	NR	NR	NR	NR	NR	NR	NR	NR	No	NR	Yes 9.5 %	Yes – 18.7 %	Yes – 2.5 %	Yes – 20 %	Yes – 6 %
Agha-Jaffar R , 2019 United Kingdom case–control study	Yes 20 %	Yes – 3 stillbirths (3.8 %); no neonatal deaths	No	Yes 13.0%	Yes 27.3%	Yes 15.6%	Yes – 3 cases (3.8 %)	NR	NR	NR	Yes – 3 stillbirths; no neonatal deaths	NR	Yes	Yes	Yes	NR	Yes	NR	Yes – 3.8 %	Yes – 1 stillbirth (1.3 %); no neonatal deaths	No	Yes – 2.5 %	Yes – 17.7 %	Yes – 11.4 %	Yes – 1 case (1.3 %)	NR	NR	NR	Yes – 1 stillbirth (1.3 %); no neonatal deaths	NR	Yes	Yes	Yes	NR	Yes	NR
Bashir M, 2019 Qatar retrospective cohot study	Yes – 27.7 %	Yes – Stillbirth 1.3 %; no neonatal deaths reported.	No	Yes – 6.5 %	Yes – 23.2 %	Yes – 5.2 %..	Yes – 1.3 %.	NR	NR	NR	Yes – Stillbirth 1.3 %; no neonatal death	NR	Yes – 23.8 %.	Yes – 9.8 %.	Yes – 10.2 %	NR	Yes – 15.3 %	NR	Yes – 11.1 % Yes – 17.3 %	Yes – Stillbirth 0.8 %; no neonatal deaths Yes – Stillbirth 0.9 %; no neonatal deaths reported	No	Yes – 14.2 %. Yes – 8.1 %	Yes – 6.7 % Yes – 13.5 %.	Yes – 4.2 % Yes – 4.7 %.	Yes – 0.8 %. Yes – 0.9 %	NR	NR	NR	Yes – Stillbirth 0.8 %; no neonatal deaths. Yes – Stillbirth 0.9 %; no neonatal deaths	NR	Yes – 8.0 %. Yes – 12.7 %.	Yes – 4.7 %. Yes – 7.2 %.	Yes – 2.8 %. Yes – 6.5 %.	NR	Yes – 9.7 % Yes – 12.6 %..	NR
Ásbjörnsdóttir B, 2019 Denmark Retrospective cohort study	NR	NR	NR	NR	NR	NR	NR	NR	NR	NR	NR	NR	NR	NR	NR	NR	NR	NR	NR	NR	NR	NR	NR	NR	NR	NR	NR	NR	NR	NR	NR	NR	NR	NR	NR	NR
Egan A, 2019 Republic of Ireland Retrospective study	Yes – 18.6 % <37 weeks; median GA 38.0 (IQR 37.3–40.0).	Yes – 10 miscarriages (17.9 %); 0 stillbirths.	No – 0 %	Yes – 7.0 %.	Yes – 16.3 %.	Yes – 9.3 %	No	NR	NR	NR	No	NR	Yes – 34.9 %	NR	Yes	NR	NR	NR	Yes – 29.9 % <37 weeks; median GA at delivery 37.2 (IQR 36.0–38.7).	Yes – 13 miscarriages (10.6 %) and 3 stillbirths (2.4 %).	Yes – 0.9 % (1 case; ventricular septal defect).	Yes – 4.7 %.	Yes – 45.8 %	Yes – 19.6 % (>4000 g)	Yes – 3 cases (2.4 %)	NR	NR	NR	No	NR	Yes – 53.3 %.	NR	Yes	NR	NR	NR
Kong L, 2019 Finland not clear	Yes – 10.1 % < 37 weeks.	NR	NR	Yes – 2.9 %..	Yes – 12.8 %.	NR	NR	NR	NR	NR	NR	NR	NR	NR	NR	NR	NR	NR	Yes – 5.3 % < 37 weeks Yes – 37.1 % < 37 weeks.	NR	NR	Yes – 3.4 % Yes – 1.5 %	Yes – 1.9 % Yes – 39.6 %.	NR	NR	NR	NR	NR	NR	NR	NR	NR	NR	NR	NR	NR
Da Rocha Oppermann	Yes – 35.9%,	Yes – 33 per 1000 births	NR	Yes – 3.1%	NR	NR	Yes	NR	NR	NR	Yes – perinat	NR	Yes – 56%	NR	NR	NR	NR	NR	Yes – 42.3%,	Yes – 71 per 1000 births	NR	Yes – 13.3%	NR	NR	Yes	NR	NR	NR	Yes – perinat	NR	Yes – 36%	NR	NR	NR	NR	NR

ML, 2019 Brazil Retrospective cohort study	mean 36.1 ± 3.3 weeks										al mortality 3.3%								mean 35.5 ± 3.5 weeks										al mortality 7.1%							
Mackin S, 2019 United Kingdom retrospective cohort study	Yes – 31%, mean gestational age 37.0 ± 2.1 weeks	Yes – 6/373 (1.6%) stillbirths	Yes – 3.2%, mostly cardiac defects and neural tube defects	Yes – 8.2%	Yes – 36%	Yes – 20%	Yes – 1.6% (stillbirths)	NR	NR	NR	Yes – 1.6% (stillbirth)	NR	Yes – 28%	Yes – 10%	Yes – 34%	NR	Yes – 7%	NR	Yes – 38%, mean gestational age 36.4 ± 2.6 weeks	Yes – 16/3,474 (0.46%) stillbirths	Yes – 4.5%, mostly cardiac defects and neural tube defects	Yes – 9.6%	Yes – 48%	Yes – 26% (≥4 kg)	Yes – 0.46% (stillbirths)	NR	NR	NR	Yes – 0.46% (stillbirth)	NR	Yes – 42%	Yes – 14%	Yes – 55%	NR	Yes – 9%	NR
Stogianni A, 2019 Greece Retrospective study	Yes – 27.7%, mean GA 37.5 ± 2.3 weeks	Yes – 1.5% stillbirths	Yes – 3.1%, mostly cardiac and neural tube defects	Yes – 7.7%	Yes – 32.3%	Yes – 15.4% (≥4000 g)	Yes – 1.5% (included in stillbirths)	NR	NR	NR	Yes – 1.5% stillbirths	NR	Yes – 33.8%	Yes – 6.9%	Yes – 45.3%	NR	Yes – 9.2%	NR	Yes – 34.2%, mean GA 37.1 ± 2.4 weeks	Yes – 1.5% stillbirths	Yes – 4.7%, mostly cardiac and neural tube defects	Yes – 5.7%	Yes – 42.0%	Yes – 19.6% (≥4000 g)	Yes – 1.0% (included in stillbirths)	NR	NR	NR	Yes – 1.5% stillbirths	NR	Yes – 38.3%	Yes – 8.2%	Yes – 51.8%	NR	Yes – 11.4%	NR
Wang X, 2019 USA Retrospective cohort study	NR	NR	NR	NR	Yes	Yes	NR	NR	NR	NR	NR	NR	NR	NR	NR	NR	NR	NR	NR	NR	NR	NR	NR Yes T1DM	NR Yes	NR	NR	NR	NR	NR	NR	NR	NR	NR	NR	NR	NR
Wernimont , S 2019 USA Prospective cohort study	Yes – mean 36.9 ± 0.6 weeks	NR	NR	NR	Yes – 28 %	NR	NR	NR	NR	NR	NR	NR	NR	NR	Yes – 61 %	NR	NR	NR	No Yes – mean 37.5 ± 2.0 weeks	NR	NR	NR	NR	NR	NR	NR	NR	NR	NR	NR	NR	NR	Yes – 43 % Yes – 57 %	NR	NR	NR
Ali D, 2020 Ireland Retrospective study	NR	Yes	Yes 2.7%	NR	Yes 30%	No	Yes 8.1%	NR	NR	NR	NR	NR	Yes 32%	NR	Yes 31.3%	NR	NR	NR	NR	Yes	Yes 6.3%	NR	Yes 20.6%	Yes 7.2%	Yes 2.4%	NR	NR	NR	NR	NR	Yes 52.2%	NR	Yes 31.3%	NR	NR	NR
López-de-Andrés A, 2020 Spain Retrospective cohort study	Yes 660 (15.03%) T2DM 1,221 (21.96%) T1DM 10,298 (7.86%) GDM	NR	NR	NR	Yes	NR	NR	NR	NR	NR	NR	NR	NR	NR	NR	NR	NR	NR	Yes 140,101 (5.99%)	NR	NR	NR	Yes	NR	NR	NR	NR	NR	NR	NR	NR	NR	NR	NR	NR	NR
Ásbjörnsdóttir B, 2020 Denmark Prospective cohort study	Yes – 19 % T2DM	No	2 cases (≈4%) minor anomalies, not specified	Yes 7%	Yes 15%	NR	No	NR	NR	NR	No	NR	NR	Yes 7% transient tachypnea (CPAP >60 min)	22% (<2.2 mmol/L)	NR	Yes 17% (required phototherapy)	NR	No	NR	No	NR	NR	NR	No	NR	NR	NR	No	NR	NR	NR	NR	NR	NR	NR
Hauffe F, 2020 German Retrospective cohort study	Yes 17 (14.4) <37 weeks 2 (1.7) <34 weeks	NR	Yes 2 (1.7)	Yes 10 (8.5)	Yes 33 (28.0)	NR	Yes 1 (0.8)	NR	NR	NR	No	NR	Yes 32 of 117 (27.4)	NR	Yes 16 of 177 (13.7)	NR	NR	NR	Yes 44 (20.2) <37 weeks 15 (6.9) <34 weeks	NR	Yes 7 (3.2)	Yes 7 (3.2)	Yes 88 (40.4)	NR	Yes 4 (1.8)	NR	NR	NR	No	NR	Yes 90 of 214 (42.1)	NR	Yes 50 of 214 (23.4)	NR	No	NR
Kattini R, 2020 Canada Retrospective study	Yes 14%	No	NR	Yes 5%	Yes 28%	Yes 22%	No	NR	NR	NR	No	NR	Yes 22%	Yes 11%	Yes 28%	NR	Yes 15%	NR	Yes 8%	No	NR	Yes 4%	Yes 28%	Yes 22%	No	NR	NR	NR	No	NR	Yes 13%	Yes 6%	Yes 17%	NR	Yes 12%	NR
Longmore D, 2020 Australia not clear	Yes	NR	NR	NR	NR	NR	NR	NR	NR	NR	NR	NR	Yes 65% / 71%	NR	Yes 77% / 62%	NR	NR	NR	No	NR	NR	NR	NR	NR	NR	NR	NR	NR	NR	NR	Yes 33% / 24% GDM 17% / 18% noDM	NR	Yes GDM 48% / 50% noDM 5% / 5%	NR	NR	NR
Morikawa M, 2020 Japan Retrospective cohort study	Yes	NR	NR	Yes T2DM + HDP: 33.3% T2DM without CH: 5.3%	NR	NR	NR	NR	NR	NR	NR	NR	NR	NR	NR	NR	NR	NR	Yes	NR	NR	Yes T1DM + HDP: 0% T1DM bez HDP: 2.9%	NR	NR	NR	NR	NR	NR	NR	NR	NR	NR	NR	NR	NR	NR
Starikov R, 2020 USA Retrospective cohort study	Yes. Preterm <37w: 41.0% SGA (16/39), 22.2% AGA (20/90). GA at delivery: SGA 37 w (39–33), AGA 39 w (39–37).	Yes (fetal) – IUFD 1 case u SGA. Neonatal loss = NR	NR	NR	NR	NR	Yes – 1/39 (2.6%) u SGA grupi; 0 u AGA.	NR	NR	NR	NR	NR	Yes. SGA: 44.7% (17/39), AGA: 28.4% (25/90).	NR	NR	NR	NR	NR	Yes. Preterm <37w: 54.6% SGA (12/22), 36.5% AGA (23/63). GA at delivery: SGA 36 w (37–32), AGA 37 w (39–32).	Yes (fetal) – IUFD 1/22 u SGA. Neonatal loss = NR.	NR	NR	NR	NR	Yes – 1/22 u SGA; 0 u AGA.	NR	NR	NR	NR	NR	Yes. SGA 66.7% (14/21–22), AGA 42.9% (27/63).	NR	NR	NR	NR	NR
Gualdani E, 2021 Italy not clear	Yes 25.6%	NR	Yes 0.9%	NR	Yes 12.5%	Yes 7.1%	NR	NR	NR	NR	NR	NR	NR	NR	NR	NR	NR	NR	Yes T1DM - 33.2% noDM - 6.6%	NR	Yes T1DM 3.8% noDM 1.0%	NR	Yes T1DM 28.4% noDM 7.9%	Yes T1DM 16.4% noDM 6.6%	NR	NR	NR	NR	NR	NR	NR	NR	NR	NR	NR	NR
Guarnotta V, 2021 Italy Retrospectiv	Yes	NR	Yes	NR	Yes	Yes	NR	NR	NR	NR	NR	NR	NR	NR	Yes	NR	NR	NR	Yes	NR	Yes	NR	Yes	Yes	NR	NR	NR	NR	NR	NR	NR	NR	Yes	NR	NR	NR

e cohort study																																				
Martínez-Cruz C, 2021 Mexico Prospective study	Yes 84% (59/70)	NR	NR	NR	NR	NR	NR	NR	NR	NR	NR	NR	NR	NR	Yes 8 (2.5%)	Yes 6 (1.9%)	Yes 32 (9.9%)	NR	Yes 94% (89/95) T1DM 89% (90/101) GDM 47% (27/57) noDM	NR	NR	NR	NR	NR	NR	NR	NR	NR	NR	NR	NR	NR	Yes 7 (2.2%) T1DM 4 (1.4%) GDM 2 (0.6%) noDM	Yes 2 (0.6%) T1DM No GDM, noDM	Yes 51 (15.8 %) T1DM 52 (16.1 %) GDM 5 (1.5%) noDM	NR
McLean A, 2021 Australia not clear	NR	NR	NR	NR	NR	NR	NR	NR	NR	NR	NR	NR	Yes	Yes	Yes	NR	NR	NR	NR	NR	NR	NR	NR	NR	NR	NR	NR	NR	NR	NR	Yes	Yes	Yes	NR	NR	NR
Saquib S, 2021 United Arab Emirates Retrospective study	Yes (27%), GA 36.9 ± 1.9	NR	Yes (5%), which: NR	Yes (10%)	Yes (25%)	Yes (8%)	NR	NR	Yes (0.8%)	NR	NR	NR	Yes (31%)	Yes (5%)	Yes (52%)	NR	Yes (29%)	NR	Yes (50%), GA 35.8 ± 2.2	NR	Yes (7%), which: NR	Yes (10.7%)	Yes (21%)	Yes (7%)	NR	NR	Yes (3.6%)	NR	NR	NR	Yes (42%)	Yes (14%)	Yes (50%)	NR	Yes (25%)	NR
Seah JM, 2021 Australia Retrospective study	Yes (38%, GA 35.6 ± 3.5)	Yes 2% T2DM	Yes 12% T2DM	Yes 14% T2DM	Yes 40% T2DM	Yes 20% T2DM	Yes 2% T2DM	NR	Yes 5% T2DM	NR	No	NR	Yes 52% T2DM	Yes 16% T2DM	Yes 64% T2DM	NR	Yes 25% T2DM	NR	Yes (8%, GA 38.1 ± 1.6) Yes (35%, GA 36.3 ± 2.8)	No Yes 1% T1DM	Yes 1% 9% T1DM	Yes (7%) 11% T1DM	Yes (11%) 31% T1DM	Yes (5%) 15% T1DM	No Yes 1% T1DM	NR	Yes (2%) 4% T1DM	NR	No	NR	Yes (12%) 48% T1DM	Yes NoDM (4%) 12% T1DM	Yes noDM (7%) 58% T1DM	NR	Yes NoDM (11%) 20% T1DM	NR
Britten F, 2022, Australia Not clear	NR	NR	NR	NR	NR	NR	NR	NR	NR	NR	NR	NR	NR	NR	Yes	NR	NR	NR	NR	NR	NR	NR	NR	NR	NR	NR	NR	NR	NR	NR	NR	NR	NR	NR	NR	NR
Rao C, 2022 China Prospective cohort study	NR	NR	Yes	NR	NR	4 (9.3%)	NR	NR	NR	NR	NR	NR	NR	NR	NR	NR	NR	NR	NR	NR	Yes Yes	NR	NR	3 (12.5 %) 7 (13.0 %)	NR	NR	NR	NR	NR	NR	NR	NR	NR	NR	NR	NR
Jacobsen D, 2022 Norway not clear	NR	NR	NR	NR	NR	NR	NR	NR	NR	NR	NR	NR	NR	NR	NR	NR	NR	NR	NR	NR	NR	NR	NR	NR	NR	NR	NR	NR	NR	NR	NR	NR	NR	NR	NR	NR
Kapustin R, 2022 Russia Retrospective cohort study	NR	NR	NR	NR	NR	Yes 15.7–17.9%	NR	Yes, shoulder dystocia T2DM diet: 5.3% T2DM insulin: 3.0%	NR	NR	NR	NR	NR	NR	NR	NR	NR	NR	NR	NR	NR	NR	NR	Yes 8.6%	NR	No	NR	NR	NR	NR	NR	NR	NR	NR	NR	NR
Kapustin R, 2022a Russia Prospective cohort study	Yes 15%	NR	NR	Yes Insulin 10%	NR	Yes Diet 1 (6.7) Insulin 2 (10)	NR	NR	NR	NR	NR	NR	NR	NR	NR	NR	NR	NR	Yes No planned 2 (10%) Planned 1 (5%)	NR	NR	Yes No planned 5%	NR	Yes T1DM No planned 6 (30%) Planned 5 (25%)	NR	NR	NR	NR	NR	NR	NR	NR	NR	NR	NR	NR
Cordero L, 2022 USA Retrospective cohort study	Yes 13%	No	No	Yes 3%	Yes 3%8	Yes 22%	No	NR	NR	NR	No	NR	Yes 22%	NR	Yes 41%	NR	NR	NR	Yes 26%	No	No	Yes 2%	Yes 42%	Yes 18%	No	NR	NR	NR	No	NR	Yes 44%	NR	Yes 58%	NR	NR	NR
Cordero L, 2022 USA Retrospective cohort study	Yes (17%)	NR	No	Yes 3%	Yes 39%	Yes 22%	No	NR	NR	NR	No	NR	Yes 23%	NR	Yes 42%	NR	NR	NR	Yes (31%)	NR	No	Yes 2%	Yes 43%	Yes 21%	No	NR	NR	NR	No	NR	Yes 45%	NR	Yes 59%	NR	NR	NR
Malaza N, 2023 South Africa Prospective study	Yes T2DM: 54.4%	Yes T2DM (6.9%)	NR	YES T2DM (6.3%)	YES T2DM(15.6 %)	Yes T2DM 4 (6.9%)	Yes T2DM 4 (6.9%)	NR	NR	NR	NR	NR	NR	NR	NR	NR	NR	NR	Yes 17.1% T1DM: 66.7% GDM: 41.9%	No Yes T1DM (10%) GDM (3.1%)	NR	No 0% NoDM Zes T1DM (7.3%) NO GDM (0%)	Yes 33.3% T1DM (34.5%) NO GDM (0%)	Yes 2 (5.7%) No T1DM 0 GDM 0	No NoDM Yes T1DM 1 (10%) GDM 1 (3.1%)	NR	NR	NR	NR	NR	NR	NR	NR	NR	NR	NR
Koyama M, 2023 Japan Not clear	Yes – 5/15	No	No	NR	NR	NR	No	NR	NR	NR	No	NR	6/15 (40%)	NR	Yes	NR	NR	NR	Yes – 2/22	No	Yes – congenital heart anomaly	NR	NR	NR	No	NR	NR	NR	No	NR	10/22 (45.5%)	NR	Yes	NR	NR	NR
Powers Carson J, 2024 USA case-control study	NR	NR	NR	NR	NR	NR	No	NR	NR	NR	No	NR	NR	NR	NR	NR	NR	NR	NR	NR	NR	NR	NR	NR Yes	No	NR	NR	NR	No	NR	NR	NR	NR	NR	NR	NR
Kapustin R, 2024 Russia retrospective cohort study	YES – 25.2%	NR	NR	YES – 7.3%	YES – 32.2%	NR	NR	NR	NR	YES – 3.7%	NR	NR	YES – 17.8%	YES – 3.7%	YES – 21.5%	NR	NR	NR	YES – 40.2% YES – 12.1%	NR	NR	YES – 5.1% YES – 9.7%	YES – 20.8% YES – 16.9%	NR	NR	NR	NR	YES – 5.6% YES – 1.4%	NR	NR	YES – 17.9% YES – 6.2%	YES – 6.8% YES – 1.0%	YES – 33.5% YES – 4.4%	NR	NR	NR
Ballesteros M, 2024 Spain prospective cohort study	Yes 10.2%	Yes (2.6%)	Yes 10.8%	Yes 6.2%	Yes 36.1%	Yes 10.4%	Yes 2.6%	Yes 2%	NR	NR	NR	NR	Yes 19.4%	Yes 7.1%	Yes 8.9%	NR	NR	NR	Yes 9.1%	Yes 0.8%	Yes 3.5%	Yes 3.4%	Yes 45.3%	Yes 15.3%	Yes 0.8%	Yes 0.9%	NR	NR	NR	NR	Yes 21.5%	Yes 7.1%	Yes 24.6%	NR	NR	NR

Grazia Dalfrà M, 2024 Italy retrospective study	Yes<br>37.5 ± 2.5 nedelja	Yes<br>4.6% (2)	Yes<br>7.7% (5)	Yes<br>8.5% (5)	Yes<br>22% (13)	NR	NR	NR	NR	NR	Yes<br>4.6% (2)	NR	NR	Yes<br>6.1% (3)	Yes<br>1.7% (1)	Yes<br>1.7% (1)	Yes<br>10.2% (5)	NR	Yes<br>38.3 ± 1.17 nedelja	No	Yes<br>11.1% (2)	13.3% (2)	20% (3)	NR	NR	NR	NR	NR	No	NR	NR	No	No	No	No	NR
Suzuki T, 2024 Japan retroscpetcive study	No	NR	NR	Yes<br>5.8%	Yes<br>29.0%	NR	NR	NR	NR	NR	NR	NR	Yes<br>13.0%	NR	NR	NR	NR	NR	No	NR	NR	Yes<br>6.7%	Yes<br>17.8%	NR	NR	NR	NR	NR	NR	NR	Yes<br>22.2%	NR	NR	NR	NR	NR
Dias S, 2025 South Africa Not clear	Yes	NR	NR	NR	NR	NR	NR	NR	NR	NR	NR	NR	NR	NR	NR	NR	NR	NR	Yes	NR	NR	NR	NR	NR	NR	NR	NR	NR	NR	NR	NR	NR	NR	NR	NR	NR
Gherbon A, 2025 Romania Retrospective study	Yes<br>28 (26.17%)	Yes<br>1 (0.93%)	NR	Yes<br>4 (5.4%)	Yes<br>11 (14.8%)	Yes<br>10<br>(13.5%)	No	NR	NR	NR	No	NR	NR	NR	Yes<br>6 (8.10%)	NR	Yes<br>13<br>(17.5%)	NR	Yes<br>111<br>(11.59%)<br>43<br>(15.41%)<br>22<br>(29.73%)	Yes<br>Cont 7<br>(0.73%)<br>GDM 3<br>(1.07%)<br>No - T1DM	NR	Cont 32<br>(3.34%)<br>GDM 12<br>(4.3%)<br>T1DM 4<br>(5.4%)	Cont 47<br>(4.9%)<br>GDM 21<br>(7.5%)<br>T1DM 11<br>(14.8%)	Cont<br>42<br>(4.38<br>%)<br>GDM<br>24<br>(8.6%)<br>T1DM<br>10<br>(13.5<br>%)	Yes<br>Cont 2<br>(0.20%<br>)<br>GDM 1<br>(0.36%<br>)<br>No<br>T1DM	NR	NR	NR	Yes<br>Cont 5<br>(0.52%<br>)<br>GDM<br>2<br>(0.71%<br>)<br>No<br>T1DM	NR	NR	NR	Yes<br>Cont 3<br>(0.31%)<br>GDM 9<br>(3.23%)<br>T1DM<br>6<br>(8.10%)	NR	Yes<br>Cont<br>38<br>(3.97<br>%)<br>GDM<br>24<br>(8.6%)<br>T1DM<br>13<br>(17.5<br>%)	NR
Hillick D, 2025 Ireland Retrospective cohort study	Yes – 31.6%	NR	Yes- 10.5%	Yes<br>15.8%	Yes<br>10.5%	NR	NR	NR	No	31.60<br>%	No	NR	Yes<br>100%	21.10%	26.30%	NR	10.50%	NR	Yes –<br>53.3%<br>Yes –<br>23.4%<br>No	NR	Yes<br>17.9%<br>7.1%<br>33.3%	Yes<br>14.3%<br>27.0%<br>33.3%	Yes<br>17.9%<br>6.0%<br>No	NR	NR	NR	Yes<br>3.6%<br>4.7%<br>No	Yes<br>42.9%<br>25.3%<br>33.3%	No	NR	Yes<br>100%<br>100%<br>100%	Yes<br>39.3%<br>15.7%<br>33.3%	Yes<br>32.1%<br>19.5%<br>No	NR	Yes<br>14.3%<br>10.5%<br>No	NR
Manga J, 2025 South Africa Retrospective study	Yes<br>3 (18.8%)	Yes	No	NR	NR	Yes -<br>13	Yes<br>3	NR	NR	NR	NR	NR	NR	Yes<br>21	Yes<br>41	NR	NR	NR	Yes<br>2 (12.5%)<br>6 (37.5%)<br>5 (31.2%)	Yes	Yes<br>T1DM 1<br>GDM - 0<br>ODM - 1	NR	NR	Yes<br>4<br>6<br>4	Yes<br>2<br>4<br>No<br>ODM-<br>0	NR	NR	NR	NR	NR	NR	Yes<br>8<br>27<br>19	Yes<br>10<br>53<br>30	NR	NR	NR
Zhou X, 2025 China retrospective cohort study	NR	NR	NR	NR	NR	NR	NR	NR	NR	NR	NR	NR	NR	NR	NR	NR	NR	NR	NR	NR	NR	NR	NR	NR	NR	NR	NR	NR	NR	NR	NR	NR	NR	NR	NR	NR
