# Supplementary material for: Body Weight-Related Parameters in Pregnancies Complicated by Type 2 Diabetes Mellitus: A Systematic Review and Meta-Analysis with Maternal and Perinatal Outcome Mapping
Source: J Clin Med. 2026 Jul 6;15(13):5260. doi: 10.3390/jcm15135260 (PMC13362816; doi:10.3390/jcm15135260)
Supplement: Supplementary file 1 [file jcm-15-05260-s001.zip › Supplementary Table S5b. Maternal adverse outcomes.pdf]

Supplement material

Table S5b. Maternal adverse outcomes

	MATERNAL CASES OUTCOMES										MATERNAL CONTROLS OUTCOMES									
Author, year Country Study design	Misscarriage	Infections during pregnancy	PIH Yes/No	PE Yes/No	Hemolysis Yes/No	Elevated liver enzymes Yes/No	HELLP syndrome Yes/No	Polyhydramnion	Oligohydramnion	Postpartal hemorrhage	Misscarriage	Infections during pregnancy	PIH Yes/No	PE Yes/No	Hemolysis Yes/No	Elevated liver enzymes Yes/No	HELLP syndrome Yes/No	Polyhydramnion	Oligohydramnion	Postpartal hemorrhage
Barnard R, 1997 Australia, Not clear	NR	NR	NR	NR	NR	NR	NR	NR	NR	NR	NR	NR	NR	NR	NR	NR	NR	NR	NR	NR
Ben Slama C, 1997 Tunis Retrospective study	Yes	Yes	Yes	Yes	NR	NR	NR	Yes	NR	NR	Yes	Yes	Yes	Yes	NR	NR	NR	Yes	NR	NR
Colatrella A, 2009 Italy Not clear	NR	NR	Yes 17.1%	Yes 5.3%	NR	NR	NR	NR	NR	NR	NR	NR	Yes 8.3%	Yes 1.3%	NR	NR	NR	NR	NR	NR
Contreras-Soto J, 1991 Mexico NR	NR	16 (13.55%) urinary	17 (14.40%)	5 (4.24%)	NR	NR	NR	4 (3.39%)	NR	NR	NR	8 (8.88%) urinary	6 (6.66%)	3 (3.33%)	NR	NR	NR	1 (1.11%)	NR	NR
Cundy T, 2000 New Zealand Not clear	1 (1.4%)	3 (4.2%)	9 (12.7%)	3 (4.2%)	NR	NR	NR	2 (2.8%)	NR	1 (1.4%)	2 (2%)	4 (4%)	17 (17%)	9 (9%)	NR	NR	NR	5 (5%)	NR	2 (2%)
Cundy T, 2002 New Zealand NR	NR	NR	11%	7%	NR	NR	NR	NR	NR	NR	NR	NR	15%	6%	NR	NR	NR	NR	NR	NR
Cundy T, 2007 New	NR	NR	NR	NR	NR	NR	NR	NR	NR	NR	NR	NR	NR	NR	NR	NR	NR	NR	NR	NR

Zealand Not clear																				
Falhammar H, 2010 Australia Not clear	NR	NR	18%	NR	NR	NR	NR	NR	NR	NR	NR	NR	NR	NR	NR	NR	NR	NR	NR	NR
Lapolla A, 2008 Italy Prospective study	10.7 % (17)	NR	9.1 % (15)	3.1 % (5)	NR	NR	NR	NR	NR	NR	6.3 % (30)	NR	12.8 % (65)	4.3 % (22)	NR	NR	NR	NR	NR	NR
Marin J 2010, Romania Retrospective study	1.44 ± 2 NS	NR	NR	NR	NR	NR	NR	NR	NR	NR	1.44 ± 1.56	NR	NR	NR	NR	NR	NR	NR	NR	NR
Min Y, 2005 UK NR	NR	NR	1 case	NR	NR	NR	NR	NR	NR	NR	NR	NR	NR	NR	NR	NR	NR	NR	NR	NR
Abrão Szylit N, 2007 Brazil Cross-sectional study	NR	NR	NR	NR	NR	NR	NR	NR	NR	NR	NR	NR	NR	NR	NR	NR	NR	NR	NR	NR
Gonzalez-Gonzalez NL., 2008, Spain retrospective study	NR	NR	NR	NR	NR	NR	NR	NR	NR	NR	NR	NR	NR	NR	NR	NR	NR	NR	NR	NR
Olmos PR, 2009, Chile NR	NR	NR	NR	NR	NR	NR	NR	NR	NR	NR	NR	NR	NR	NR	NR	NR	NR	NR	NR	NR
Roland	NR	NR	NR	NR	NR	NR	NR	NR	NR	NR	NR	NR	NR	NR	NR	NR	NR	NR	NR	NR

JM , 2005 UK prospective study																				
Westgate JA, 2006, New Zealand Not clear	NR	NR	NR	NR	NR	NR	NR	NR	NR	NR	NR	NR	NR	NR	NR	NR	NR	NR	NR	NR
Yang X, 2002 China Prospective study	NR	NR	16.70%	2.94%	NR	NR	NR	NR	NR	NR	NR	NR	6.62%	NR	NR	NR	NR	NR	NR	NR
Cyganek K, 2011 Poland NR	10.0% (7/70	NR	NR	NR	NR	NR	NR	NR	NR	NR	7.3% (25/345	NR	NR	NR	NR	NR	NR	NR	NR	NR
Handisurya A, 2011 Austria Not clear	<12 weeks: 4 (6.1%)	NR	13 (19.7%)	7 (11.7%)	NR	NR	NR	NR	NR	NR	<12 weeks: 11 (14.7%)	NR	14 (19.2%)	11 (17.7%)	NR	NR	NR	NR	NR	NR
Knight K, 2012 USA Retrospective cohort study	NR	NR	10 4.7%	31 14.6%	NR	NR	NR	20 9.4%	16 7.5%	34 16.0%	NR	NR	19 8.9%	11 5.2%	NR	NR	NR	4 1.9%	6 2.8%	16 7.5%
Murphy H, 2011 UK Prospective cohort study	17.00%	NR	NR	5.20%	NR	NR	NR	NR	NR	NR	13.30%	NR	NR	7.80%	NR	NR	NR	NR	NR	NR
de Oliveira Baraldi C, 2012 Brazil not clear	NR	NR	NR	NR	NR	NR	NR	NR	NR	NR	NR	NR	NR	NR	NR	NR	NR	NR	NR	NR

Higgins M, 2013 Ireland Prospective cohort study	NR	NR	NR	NR	NR	NR	NR	NR	NR	NR	NR	NR	NR	NR	NR	NR	NR	NR	NR	NR
Min Y, 2014 United Kingdom RCT	NR	NR	NR	NR	NR	NR	NR	NR	NR	NR	NR	NR	NR	NR	NR	NR	NR	NR	NR	NR
Sato T, 2014 Japan Retrospective study	NR	NR	77<br>13.3%	70<br>12.1%	NR	NR	NR	NR	NR	NR	NR	NR	41<br>11.1%	32<br>8.7%	NR	NR	NR	NR	NR	NR
Hall D, 2015, South Africa Not clear	NR	NR	NR	NR	NR	NR	NR	NR	NR	NR	NR	NR	NR	NR	NR	NR	NR	NR	NR	NR
Huynh J, 2015 USA not clear	NR	NR	NR	NR	NR	NR	NR	NR	NR	NR	NR	NR	NR	NR	NR	NR	NR	NR	NR	NR
Owens L, 2015 Ireland case-control study	8.30%	NR	22%	8.30%	NR	NR	NR	6.50%	NR	3.70%	11.00%	NR	20%	12.00%	NR	NR	NR	10.00%	NR	2.80%
Park S, 2015 Korea not clear	NR	NR	NR	NR	NR	NR	NR	NR	NR	NR	NR	NR	NR	NR	NR	NR	NR	NR	NR	NR
Wright L 2015 USA not clear	NR	NR	NR	NR	NR	NR	NR	NR	NR	NR	NR	NR	NR	NR	NR	NR	NR	NR	NR	NR
Abell S, 2016 Australia retrospective cohort	NR	NR	5.10%	8.70%	NR	NR	NR	NR	NR	NR	NR	NR	2.00%	2.40%	NR	NR	NR	NR	NR	NR

study																				
Cade WT , 2016 USA NR	NR	NR	NR	NR	NR	NR	NR	NR	NR	NR	NR	NR	NR	NR	NR	NR	NR	NR	NR	NR
Hammoud N, 2016 Netherlands NR	NR	NR	NR	NR	NR	NR	NR	NR	NR	NR	NR	NR	NR	NR	NR	NR	NR	NR	NR	NR
Villarroel C, 2016, Chile prospective study	NR	NR	14.3% (3/21)	NR	NR	NR	NR	NR	NR	NR	NR	NR	NR	NR	NR	NR	NR	NR	NR	NR
Billionnet C , 2017 France cross-sectional study	NR	NR	NR	6.4 %	NR	NR	NR	NR	NR	NR	NR	NR	NR	No DM 1.6 % GDM 2.6 % (OR 1.6 [1.5–1.7])	NR	NR	NR	NR	NR	NR
Cade WT , 2017 USA Prospective study	NR	NR	NR	NR	NR	NR	NR	NR	NR	NR	NR	NR	NR	NR	NR	NR	NR	NR	NR	NR
Cnattingius S, 2017 Sweden Not clear	NR	NR	NR	7.60%	NR	NR	NR	NR	NR	NR	NR	NR	NR	2.7 % 14.2%	NR	NR	NR	NR	NR	NR
Joshi T, 2017 Australia not clear	NR	NR	NR	Yes 16 %	NR	NR	NR	NR	NR	NR	NR	NR	NR	Yes 13 %	NR	NR	NR	NR	NR	NR
Ladfors L, 2017 Sweden not clear	No	NR	Yes 9 (10%)	Yes 9 (10%)	NR	NR	NR	NR	NR	NR	No	NR	22 (10%)	22 (10%)	NR	NR	NR	NR	NR	NR
Saikia DM ,	No	NR	No	No	NR	NR	No	NR	NR	NR	No	NR	No	No	NR	NR	No	NR	NR	NR

2017 Assam prospective study																				
Villarroel C, 2017 Chile prospective study	NR	NR	NR	NR	NR	NR	NR	NR	NR	NR	NR	NR	NR	NR	NR	NR	NR	NR	NR	NR
Alessi J, 2018 Brazil Retrospective study	Yes	NR	Yes	Yes	NR	NR	NR	NR	NR	NR	Yes	NR	Yes	Yes	NR	NR	NR	NR	NR	NR
Endo S, 2018 Japan not clear	No	NR	Yes	Yes	NR	NR	NR	NR	NR	NR	No	NR	Yes	Yes 2	NR	NR	NR	NR	NR	NR
Jang HJ , 2018 South Korea case–control study	No	Yes 26 %	NR	Yes – 10 %	NR	NR	NR	NR	NR	NR	No	Yes 2 %	NR	Yes – 2 %	NR	NR	NR	NR	NR	NR
Maple-Brown LJ , 2018 Australia prospective cohort study	NR	NR	NR	NR	NR	NR	NR	NR	NR	NR	NR	NR	NR	NR	NR	NR	NR	NR	NR	NR
Scherneck S, 2018 Germany Prospective cohort study	Yes – 9.5 %	NR	NR	NR	NR	NR	NR	NR	NR	NR	Yes	NR	NR	NR	NR	NR	NR	NR	NR	NR
Shimizu	NR	NR	NR	NR	NR	NR	NR	NR	NR	NR	NR	NR	NR	NR	NR	NR	NR	NR	NR	NR

I, 2018 Japan Prospective study																				
Agha-Jaffar R , 2019 United Kingdom case–control study	NR	NR	Yes	Yes	NR	NR	NR	NR	NR	Yes – Moderate (500–1000 ml): 32.5 %; Severe (≥ 1000 ml): 8.8 %	NR	NR	Yes	Yes	NR	NR	NR	NR	NR	Yes – Moderate (500–1000 ml): 31.3 %; Severe (≥ 1000 ml): 8.8 %
Bashir M, 2019 Qatar retrospective cohot study	NR	NR	Yes – 9.7 %	Yes – 8.4 %	NR	NR	NR	Yes – 12.6 %.	NR	NR	NR	NR	Yes – 2.5 %<br>Yes – 6.4 %.	Yes – 2.5 %<br>Yes – 5.5 %	NR	NR	NR	Yes – 2.0 %<br>Yes – 9.0 %	NR	NR
Ásbjörnsdóttir B, 2019 Denmark Retrospective cohort study	NR	NR	NR	NR	NR	NR	NR	NR	NR	NR	NR	NR	NR	NR	NR	NR	NR	NR	NR	NR
Egan A, 2019 Republic of Ireland Retrospective study	Yes – 10 cases (17.9 %)	NR	NR	NR	NR	NR	NR	NR	NR	NR	Yes – 13 cases (10.6 %)	NR	NR	NR	NR	NR	NR	NR	NR	NR
Kong L, 2019 Finland not clear	NR	NR	NR	NR	NR	NR	NR	NR	NR	NR	NR	NR	NR	NR	NR	NR	NR	NR	NR	NR
Da Rocha Oppermann ML, 2019 Brazil<br><br>Retrosp	NR	NR	NR	Yes – 25%	NR	NR	NR	NR	NR	NR	NR	NR	NR	Yes – 38.5%	NR	NR	NR	NR	NR	NR

ective cohort study																				
Mackin S, 2019 United Kingdom<br><br>retrospective cohort study	NR	NR	NR	Yes – 12%	NR	NR	NR	NR	NR	NR	NR	NR	NR	Yes – 18%	NR	NR	NR	NR	NR	NR
Stogianni A, 2019 Greece<br><br>Retroscpetive study	NR	NR	Yes – 8.4%	Yes – 7.7%	NR	NR	NR	NR	NR	NR	NR	NR	Yes – 6.7%	Yes – 5.7%	NR	NR	NR	NR	NR	NR
Wang X, 2019 USA<br><br>Retrospective cohort study	NR	NR	Yes	NR	NR	NR	NR	NR	NR	NR	NR	NR	No<br>Yes<br>T1DM	NR	NR	NR	NR	NR	NR	NR
Wernimont ,S 2019 USA<br><br>Prospective cohort study	NR	NR	Yes – 18 %	NR	NR	NR	NR	NR	NR	NR	NR	NR	Yes – 11 %<br>Yes – 23 %	NR	NR	NR	NR	NR	NR	NR
Ali D, 2020 Ireland<br><br>Retrospective study	Yes 18.3%	NR	Yes 13.5%	Yes 5.4%	NR	NR	NR	NR	NR	NR	Yes 20.8%	NR	Yes 21.4%	Yes 10.5%	NR	NR	NR	NR	NR	NR
López-de-Andrés A, 2020 Spain	NR	NR	Yes	Yes	NR	NR	Yes	Yes	Yes	Yes	NR	NR	Yes	Yes	NR	NR	Yes	Yes	Yes	Yes

Retrospective cohort study																				
Ásbjörnsdóttir B, 2020 Denmark Prospective cohort study	NR	NR	NR	NR	NR	NR	NR	NR	NR	NR	NR	NR	NR	NR	NR	NR	NR	NR	NR	NR
Hauffe F, 2020 German Retrospective cohort study	NR	NR	NR	Yes 6 (5.1)	NR	NR	NR	NR	NR	NR	NR	NR	Yes 21 (9.6)	NR	NR	NR	NR	NR	NR	NR
Kattini R, 2020 Canada Retrospective study	NR	NR	NR 21%	Yes 13%	NR	NR	NR	NR	NR	NR	NR	NR	Yes 8%	Yes 3%	NR	NR	NR	NR	NR	NR
Longmore D, 2020 Australia not clear	NR	NR	NR	NR	NR	NR	NR	NR	NR	NR	NR	NR	NR	NR	NR	NR	NR	NR	NR	NR
Morikawa M, 2020 Japan Retrospective cohort study	NR	NR	Yes T2DM + HDP = 15/109 (13.8%)	Yes T2DM + HDP = 15/109 (13.8%)	NR	NR	NR	NR	NR	NR	NR	NR	Yes T1DM + HDP = 10/45 (22.2%)	Yes T1DM + HDP = 10/45 (22.2%)	NR	NR	NR	NR	NR	NR
Starikov R, 2020 USA Retrospective cohort study	NR	NR	NR	YES • SGA placenta: 10/39 (25.6%) • AGA placenta: 13/90 (14.4%)	NR	NR	NR	NR	NR	NR	NR	NR	NR	YES • SGA placenta: 6/22 (27.3%) • AGA placenta: 17/63 (27.0%)	NR	NR	NR	NR	NR	NR

Gualdani E, 2021 Italy not clear	Yes 19.8%	NR	NR	NR	NR	NR	NR	NR	NR	NR	Yes T1DM 19.8% nonDM 16.9%	NR	NR	NR	NR	NR	NR	NR	NR	NR
Guarnotta V, 2021 Italy Retrospective cohort study	NR	NR	Yes	Yes	NR	NR	NR	NR	NR	NR	NR	NR	Yes	Yes	NR	NR	NR	NR	NR	NR
Martínez-Cruz C, 2021 Mexico Prospective study	NR	NR	NR	Yes – 11.3%	NR	NR	NR	NR	NR	NR	NR	NR	NR	Yes – 7.1% T1DM Yes – 9.9% GDM No - noDM	NR	NR	NR	NR	NR	NR
McLean A, 2021 Australia not clear	NR	NR	NR	NR	NR	NR	NR	NR	NR	NR	NR	NR	NR	NR	NR	NR	NR	NR	NR	NR
Saquib S, 2021 United Arab Emirates Retrospective study	NR	NR	Yes (9%)	Yes (11%)	NR	NR	NR	Yes (12%)	Yes (9%)	Yes (8%)	NR	NR	Yes (7%)	Yes (14%)	NR	NR	NR	Yes (10%)	Yes (10%)	Yes (10%)
Seah JM, 2021 Australia Retrospective study	NR	NR	Yes (24%)	Yes (22%)	NR	NR	NR	Yes (26%)	Yes (6%)	Yes (8%)	NR	NR	Yes (6%) Yes (10%)	Yes (6%) Yes (14%)	NR	NR	NR	Yes (3%) Yes (20%)	Yes (3%) Yes (5%)	Yes (4%) Yes (11%)
Britten F, 2022, Australia Not clear	NR	NR	NR	NR	NR	NR	NR	NR	NR	NR	NR	NR	NR	NR	NR	NR	NR	NR	NR	NR
Rao C,	NR	NR	NR	NR	NR	NR	NR	NR	NR	NR	NR	NR	NR	NR	NR	NR	NR	NR	NR	NR

2022 China Prospective cohort study																				
Jacobsen D, 2022 Norway not clear	NR	NR	NR	NR	NR	NR	NR	NR	NR	NR	NR	NR	NR	NR	NR	NR	NR	NR	NR	NR
Kapustin R, 2022 Russia Retrospective cohort study	NR	NR	NR	NR	NR	NR	NR	NR	NR	NR	NR	NR	NR	NR	NR	NR	NR	NR	NR	NR
Kapustin R, 2022a Prospective cohort study	NR	NR	Yes Diet 13.3% Insulin 5 25%	Moderate PE Diet 20% Insulin 30% Severe PE Diet 6.7% Insulin 20% Early onset Diet 0% Insulin 10% Late onset Diet 26,7% Insulin 40%	NR	NR	NR	NR	NR	NR	NR	NR	Yes T1DM No planned 15% Planned 20% Healthy controls 0%	Moderate PE T1DM No planned 25% Planned 30% Severe PE T1DM No planned 15% Planned 0% Early onset T1DM No planned 5% Planned 0% Late onset T1DM No planned 35% Planned 30% Healthy controls 0% for all	NR	NR	NR	NR	NR	NR

Cordero L, 2022 USA Retrospective cohort study	No	NR	NR	Yes 5%	NR	NR	NR	NR	NR	NR	No	NR	NR	Yes 9%	NR	NR	NR	NR	NR	NR
Cordero L, 2022 USA Retrospective cohort study	No	NR	NR	Yes 5%	NR	NR	NR	NR	NR	NR	No	NR	NR	Yes 20%	NR	NR	NR	NR	NR	NR
Malaza N, 2023 South Africa Prospective study	NR	NR	Yes 23/65 (35.9%)	NR	NR	NR	NR	NR	NR	NR	NR	NR	Yes 4/66 (6.1%) 2/13 (15.4%) 12/39 (30.8%)	NR	NR	NR	NR	NR	NR	NR
Koyama M, 2023 Japan Not clear	No	NR	NR	NR	NR	NR	NR	NR	NR	NR	No	NR	NR	NR	NR	NR	NR	NR	NR	NR
Powers Carson J, 2024 USA case-control study	NR	NR	NR	NR	NR	NR	NR	NR	NR	NR	NR	NR	NR	NR	NR	NR	NR	NR	NR	NR
Kapustin R, 2024 Russia retrospective cohort study	NR	NR	NR	YES – 12.6%	NR	NR	NR	NR	NR	NR	NR	NR	YES – 14.1% YES – 4.0%	NR	NR	NR	NR	NR	NR	NR
Ballesteros M, 2024 Spain prospective cohort study	Yes 7.3%	NR	Yes 4%	Yes 4.8%	NR	NR	NR	NR	NR	NR	Yes 12.1%	NR	Yes 0.7%	Yes 4.3%	NR	NR	NR	NR	NR	NR
Grazia	Yes	NR	NR	NR	NR	NR	NR	NR	NR	NR	Yes	NR	NR	NR	NR	NR	NR	NR	NR	NR

Dalfrà M, 2024 Italy retrospective study	15.4%<br>(10)										16.6%<br>(3)									
Suzuki T, 2024 Japan retroscpetcive study	NR	NR	NR	NR	NR	NR	NR	NR	NR	NR	NR	NR	NR	NR	NR	NR	NR	NR	NR	NR
Dias S, 2025 South Africa Not clear	NR	NR	NR	NR	NR	NR	NR	NR	NR	NR	NR	NR	NR	NR	NR	NR	NR	NR	NR	NR
Gherbon A, 2025 Romania Retrospective study	Yes<br>5<br>(4.67 %)	Yes<br>7 (6.54%)	Yes9<br>(8.41%)	Yes<br>8<br>(7.47%)	NR	NR	NR	Yes<br>6<br>(5.60 %)	Yes<br>7<br>(6.54 %)	Yes<br>4 (3.73%)	Yes<br>Cont 35<br>(3.65%)<br>GDM<br>13<br>(4.66%)<br>T1DM<br>4<br>(5.40%)	Yes<br>19<br>(1.98%)<br>11<br>(3.94%)<br>5 (6.76%)	Yes<br>12<br>(1.25%)<br>16<br>(5.73%)<br>6<br>(8.10%)	Yes<br>6 (0.63%)<br>14<br>(5.02%)<br>7 (9.45%)	NR	NR	NR	Yes<br>18<br>(1.88%)<br>13<br>(4.66%)<br>4<br>(5.40%)	Yes<br>21<br>(2.19%)<br>16<br>(5.73%)<br>5 (6.76%)	Yes<br>11 (1.15%)<br>9 (3.23%)<br>3 (4.05%)
Hillick D, 2025 Ireland Retrospective cohort study	NR	NR	Yes<br>31.6%	Yes<br>31.6%	NR	NR	NR	NR	NR	NR	NR	NR	Yes<br>42.9%<br>13.7%<br>33.3%	Yes<br>42.9%<br>13.7%<br>33.3%	NR	NR	NR	NR	NR	NR
Manga J, 2025 South Africa Retrospective study	Yes<br>3	NR	NR	Yes<br>4	NR	NR	NR	NR	Yes<br>2	NR	Yes<br>1<br>1<br>2	NR	NR	Yes<br>1<br>3<br>No -<br>ODM	NR	NR	NR	Yes<br>3<br>8<br>6	NR	NR
Zhou X, 2025 China retrospective cohort study	NR	NR	NR	NR	NR	NR	NR	NR	NR	NR	NR	NR	NR	NR	NR	NR	NR	NR	NR	NR
