# Supplementary figures and images for: Body Weight-Related Parameters in Pregnancies Complicated by Type 2 Diabetes Mellitus: A Systematic Review and Meta-Analysis with Maternal and Perinatal Outcome Mapping
Source: J Clin Med. 2026 Jul 6;15(13):5260. doi: 10.3390/jcm15135260 (PMC13362816; doi:10.3390/jcm15135260)

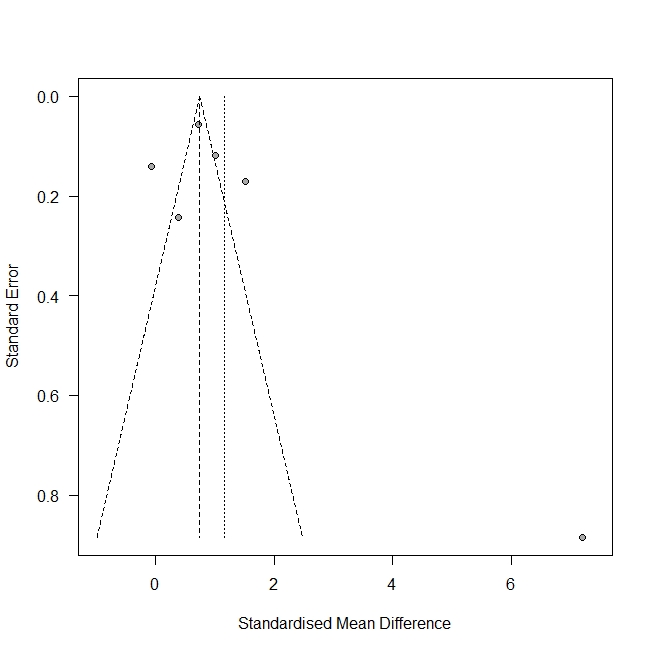

Supplement: Supplementary file 1 [file jcm-15-05260-s001.zip › Figure S1. Funnel plot - pre-pregnancy body weight T2DM vs normoglycemic controls.jpeg]

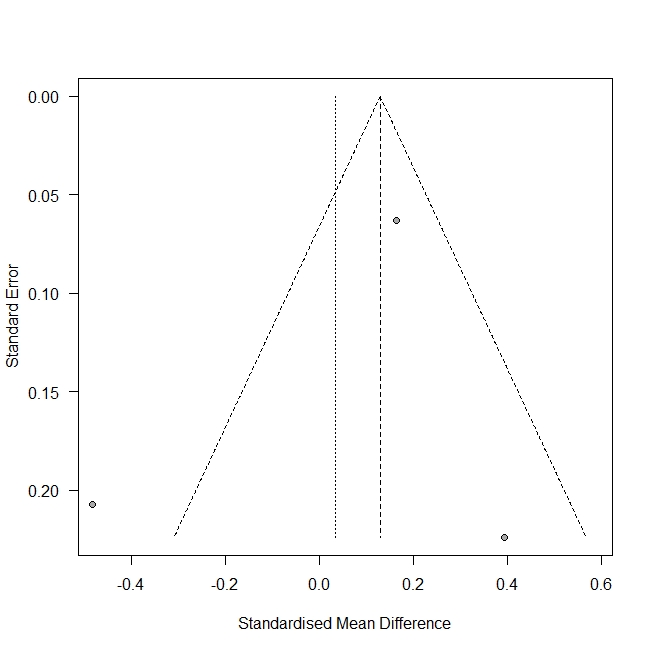

Supplement: Supplementary file 1 [file jcm-15-05260-s001.zip › Figure S10. Funnel plot - GWG T2DM vs GDM.jpeg]

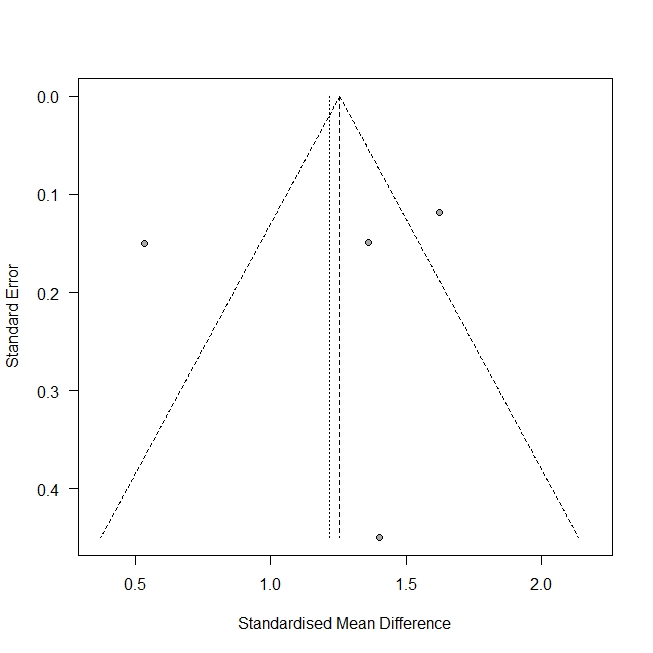

Supplement: Supplementary file 1 [file jcm-15-05260-s001.zip › Figure S2. Funnel plot - pre-pregnancy body weight T2DM vs T1DM.jpeg]

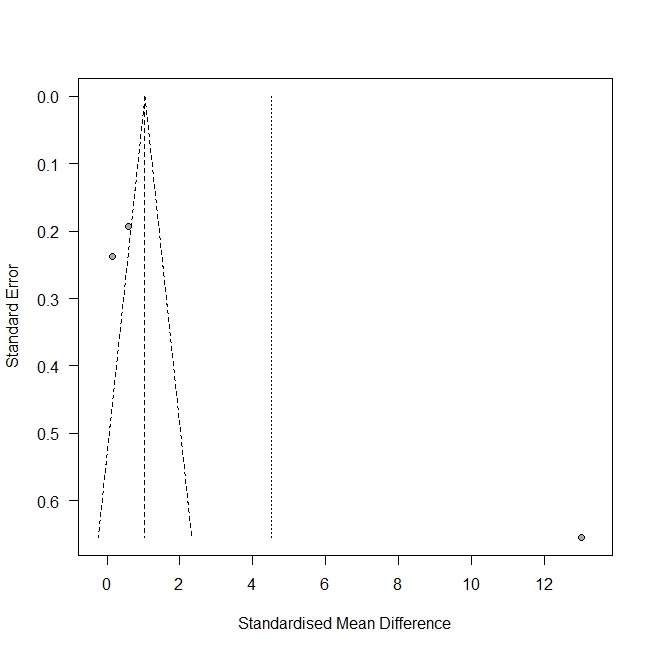

Supplement: Supplementary file 1 [file jcm-15-05260-s001.zip › Figure S3. Funnel plot - first-trimester body weight T2DM vs normoglycemic controls.jpeg]

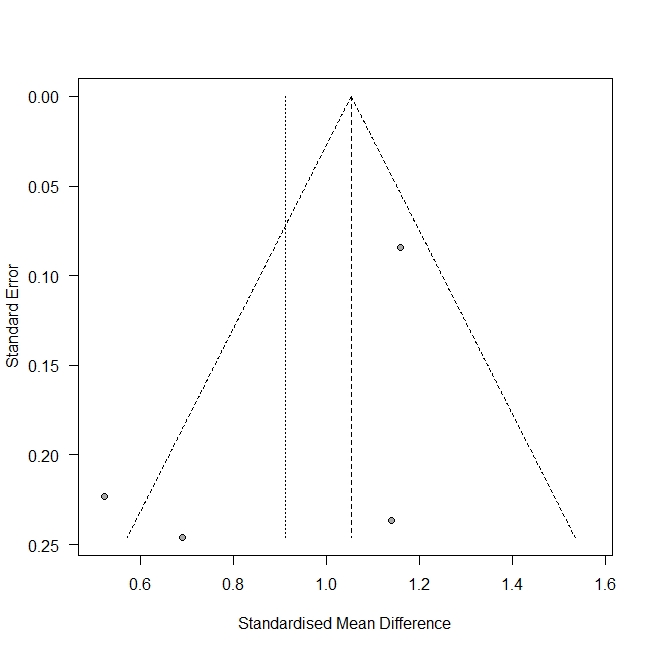

Supplement: Supplementary file 1 [file jcm-15-05260-s001.zip › Figure S4. Funnel plot - first-trimester body weight T2DM vs T1DM.jpeg]

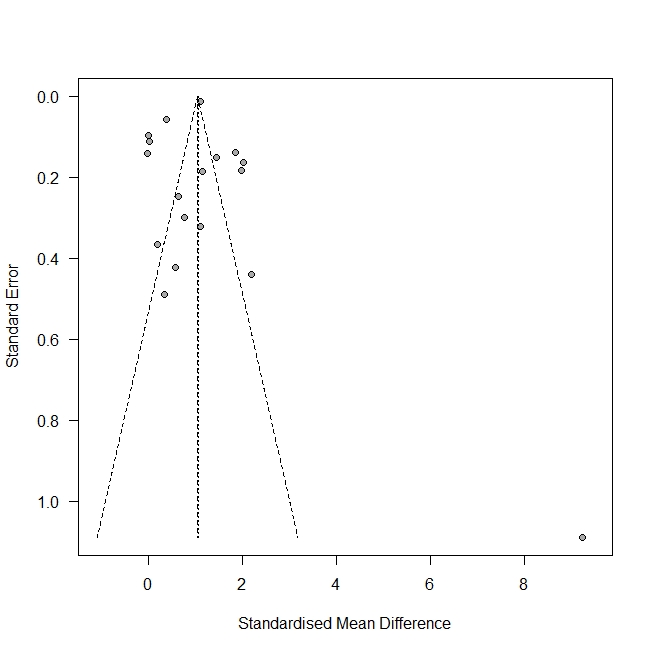

Supplement: Supplementary file 1 [file jcm-15-05260-s001.zip › Figure S5. Funnel plot - pre-pregnancy BMI T2DM vs normoglycemic controls.jpeg]

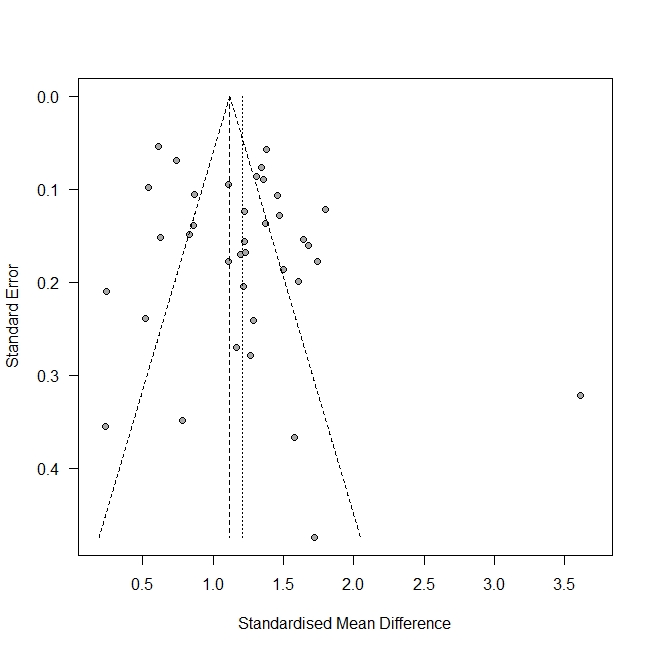

Supplement: Supplementary file 1 [file jcm-15-05260-s001.zip › Figure S6. Funnel plot - pre-pregnancy BMI T2DM vs T1DM.jpeg]

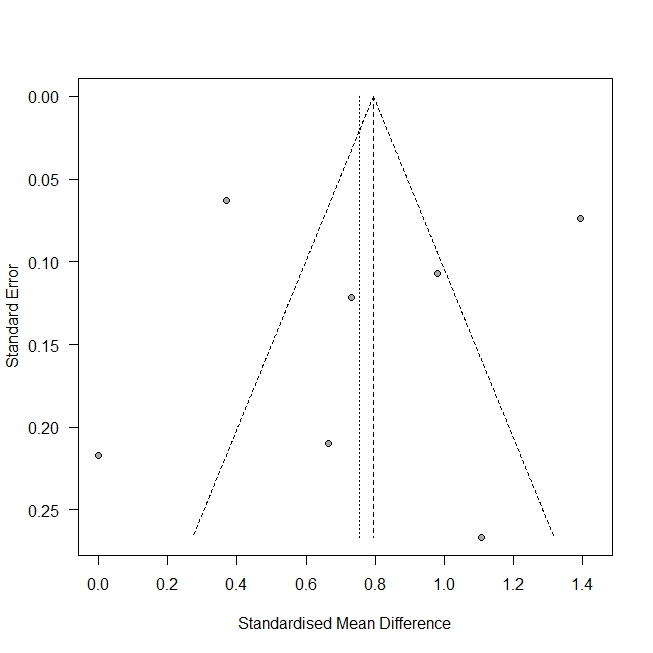

Supplement: Supplementary file 1 [file jcm-15-05260-s001.zip › Figure S7. Funnel plot - pre-pregnancy BMI T2DM vs GDM.jpeg]

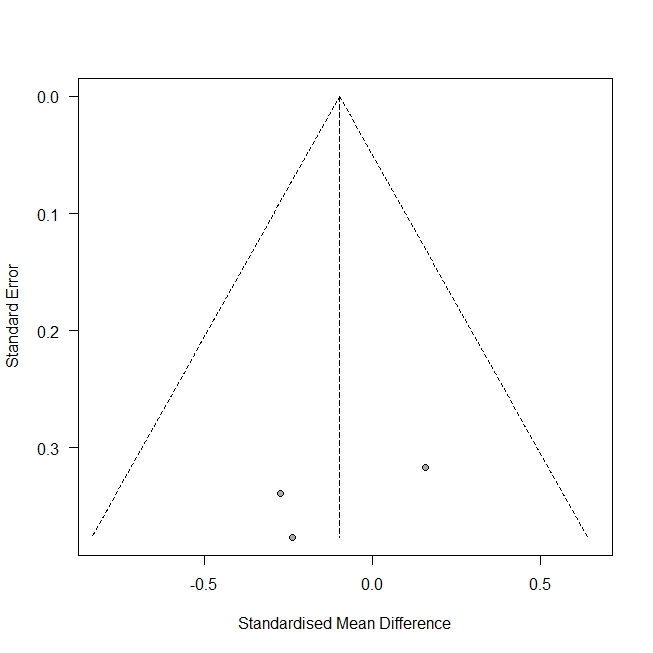

Supplement: Supplementary file 1 [file jcm-15-05260-s001.zip › Figure S8. Funnel plot - GWG T2DM vs normoglycemic controls.jpeg]

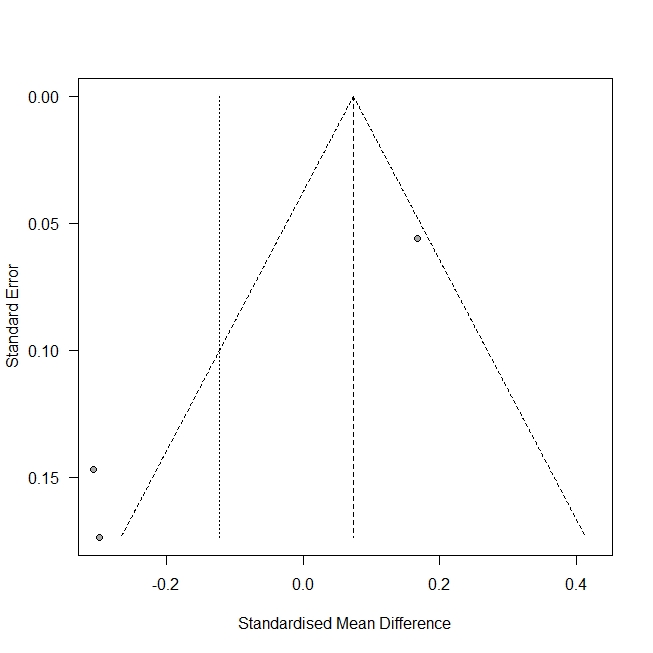

Supplement: Supplementary file 1 [file jcm-15-05260-s001.zip › Figure S9. Funnel plot - GWG T2DM vs T1DM.jpeg]
